# Metabarcoding versus mapping unassembled shotgun reads for identification of prey consumed by arthropod epigeal predators

**DOI:** 10.1093/gigascience/giac020

**Published:** 2022-03-24

**Authors:** Débora Pires Paula, Suellen Karina Albertoni Barros, Rafael Major Pitta, Marliton Rocha Barreto, Roberto Coiti Togawa, David A Andow

**Affiliations:** Embrapa Genetic Resources and Biotechnology, Brasília-DF, 70770-917, Brazil; Universidade Federal de Mato Grosso, Sinop-MT, 78550-728, Brazil; Embrapa Agrosilvopastoril, Sinop-MT, 78550-970, Brazil; Universidade Federal de Mato Grosso, Sinop-MT, 78550-728, Brazil; Embrapa Genetic Resources and Biotechnology, Brasília-DF, 70770-917, Brazil; Department of Entomology, University of Minnesota, MN, 55108, St. Paul, USA

**Keywords:** diet analysis, environmental DNA, generalist predators, gut content analysis

## Abstract

**Background:**

A central challenge of DNA gut content analysis is to identify prey in a highly degraded DNA community. In this study, we evaluated prey detection using metabarcoding and a method of mapping unassembled shotgun reads (Lazaro).

**Results:**

In a mock prey community, metabarcoding did not detect any prey, probably owing to primer choice and/or preferential predator DNA amplification, while Lazaro detected prey with accuracy 43–71%. Gut content analysis of field-collected arthropod epigeal predators (3 ants, 1 dermapteran, and 1 carabid) from agricultural habitats in Brazil (27 samples, 46–273 individuals per sample) revealed that 64% of the prey species detections by either method were not confirmed by melting curve analysis and 87% of the true prey were detected in common. We hypothesized that Lazaro would detect fewer true- and false-positive and more false-negative prey with greater taxonomic resolution than metabarcoding but found that the methods were similar in sensitivity, specificity, false discovery rate, false omission rate, and accuracy. There was a positive correlation between the relative prey DNA concentration in the samples and the number of prey reads detected by Lazaro, while this was inconsistent for metabarcoding.

**Conclusions:**

Metabarcoding and Lazaro had similar, but partially complementary, detection of prey in arthropod predator guts. However, while Lazaro was almost 2× more expensive, the number of reads was related to the amount of prey DNA, suggesting that Lazaro may provide quantitative prey information while metabarcoding did not.

## Data Description

### Background

The use of high-throughput DNA sequencing for studying species composition or diversity in environmental samples has been widely adopted, and metabarcoding has become the most commonly used method to study environmental DNA (eDNA) [1–3]. In metabarcoding, target barcode regions are enriched through PCR and sequenced for taxonomic identification (specific taxa or operational taxonomic units) through a bioinformatic workflow [4–8] by similarity of query sequences with taxonomically identified barcode sequences in a reference database [9].

The main limitations of metabarcoding are related to bias in primer amplification efficiency during the target barcode enrichment process and PCR amplification errors (e.g., point mutations, chimeras and heteroduplexes formation) [10–13]. Optimal primer pair(s) would amplify the barcode region(s) of a broad taxonomic range with equivalent efficiency across taxa, avoid formation of chimeras among closely related or abundant sequences, and provide the desired taxonomic resolution without missing any taxon [9,10,12,14]. Such a primer pair has yet to be found, so most recent metabarcoding studies have focused on amplifying barcodes of specific taxonomic groups and/or using multiple primer pairs for the same or different barcodes. In addition, for predator gut content analysis, the more common predator barcode sequences could mask amplification of closely related species.

Alternative methods for species identification that have no sample DNA enrichment have been developed, and include those that assemble or do not assemble the reads prior to mapping them to a reference database. Methods with no sample DNA enrichment in which reads are assembled include mitochondrial metagenomics [15–21], metagenome skimming [22], and enrichment of a barcode sequence by hybridization capture followed by high-throughput sequencing [23]. Mitochondrial metagenomics and metagenome skimming are promising methods for general biodiversity surveys but not for predator gut content analysis because they rely on assembling genomes (organelles or nuclear genetic material) to function as a “superbarcode” to identify species. Because the DNA community in predator guts is degraded by digestion, satisfactory assembly of prey mitochondria with sufficient coverage is difficult, and, therefore, the application of mitochondrial metagenomics and metagenome skimming is compromised. Hybridization capture replaces PCR to enrich the barcode sequences in a sample and is suitable for gut content analysis. However, because it still depends on an intermediate step of enrichment of a particular barcode, it might also be subject to bias related to probe design and fidelity/efficiency of the hybridization.

Assembly-free methods have been more recently proposed [24, 25], but just a few were tested for gut content analysis [26–29]. These methods basically comprise direct eDNA sequencing and mapping the unassembled reads to a reference database for taxa identification using a threshold of high similarity (>95%) with a minimum predefined overlap length for the matches. No barcode primer pair or probe is required, hypothetically minimizing bias and favoring quantitative estimates of prey content. Without amplification, however, the detection of rare eDNA is likely reduced. Suitable sequences of candidate prey (e.g., species co-occurring with the predators) may be missing from the database, and they need to be elucidated and added to the reference database, otherwise the prey cannot be detected. A major limitation is that different samples cannot be multiplexed in a library because there is no sample DNA enrichment step where individual tags are assigned to each sample. Consequently, every sample has to have its own library, increasing the total cost in library construction. On the other hand, they can use reference databases that can contain a variety of sequences from any part of a genome, not just metabarcodes, such as organellar or nuclear genome fragments [29].

Despite great advances and several options, at present there is no consensus on a “best practice” method for eDNA study of gut contents [9]. To improve the applicability of large-scale DNA-based methods for prey detection, we aimed to test the sensitivity, specificity, and accuracy of metabarcoding compared to a method of detection by mapping unassembled shotgun reads called Lazaro (so-called because it “resuscitates” species identifications from highly degraded DNA) to identify prey in the guts of several epigeal agricultural predators. We used melt curve analysis (MCA) to verify detections by the 2 methods and examined the number of true and false prey species detections, the number of true and false non-detections, the taxonomic resolution of true detections, and the relation between the number of reads for a detection and the relative prey DNA concentration.

## Materials and Methods

### Mock prey community

Newly emerged (48 h) unfed harlequin ladybird *Harmonia axyridis* (Coleoptera: Coccinellidae) adults (n = 10, sex ratio of 1:1) were individually supplied simultaneously with 7 species of prey, which were visually confirmed to be consumed within 1 hour. These were 1 adult aptera of the aphids (Hemiptera: Aphididae) *Aphis glycines, Aphis gossypii, Aphis craccivora, Acyrthosiphon pisum*, and *Myzus persicae*, and 1 egg each of the diamondback moth *Plutella xylostella* (Lepidoptera: Plutellidae) and *Cycloneda munda* (Coleoptera: Coccinellidae). Immediately before feeding (negative control) and after feeding, 5 beetles per sex were placed in 95% ethanol and stored at −80°C.

### Arthropod field sampling

Epigeal arthropod predators were sampled twice a month (Brazilian authorization SISBIO 33683–1) in 2014/2015 (July through September) in Sinop-MT/Brazil for a 24-h period [30] in pitfall traps buried level with the soil in 4 replicated agricultural experimental plots: soybean/maize (*Glycines max*/*Zea mays*), palisade grass (*Brachiaria brizantha*), eucalyptus plantation (hybrid of *Eucalyptus grandis* and *Eucalyptus urophylla*), and an additive mixture of all 3. Pitfall traps contained 750 mL of water and 2 drops of detergent to break surface tension and preserve the captured specimens [31]. We obtained 12 samples for each of the 2 more abundant ant species (Hymenoptera: Formicidae), *Pheidole flavens* (n = 200 specimens/sample) and *Dorymyrmex brunneus* (n = 100 specimens/sample), and 1 sample of *Solenopsis substituta* (n = 273), 1 sample of the earwig *Euborellia annulipes* (Dermaptera: Anisolabididae) (n = 46), and 1 sample of the tiger beetle *Tetracha* sp. (Coleoptera: Carabidae) (n = 49). These species were the most abundant predator species sampled, and all the specimens were used for DNA extraction.

### DNA extractions

To clean external DNA from the specimens, all the specimens from the feeding bioassay controls and from the field, before DNA extraction, were soaked individually for 40 min in 2.5% commercial bleach in 1.5 microtubes, followed by orbital rotation at 2*g* at 4°C for 40 min, discarding the washing solution and rinsing the specimens for 5× in ultrapure water [32]. For the ants, the gaster was separated and collected, and for the other species, guts were dissected under a microscope (30× magnification) immediately before DNA extraction using sterilized entomological dissecting tools. Sterilization was performed by soaking the dissection tools in 0.5% sodium hypochlorite for 10 min and autoclaving (121°C at 1 atm for 20 min), followed by rinsing abundantly with ultrapure water (MilliQ, Burlington, MA, USA) to minimize cross-contamination. Dissected guts or gasters from the same sample were pooled in a lysis buffer from the kit DNeasy Blood & Tissue (Qiagen, Germantown, MD, USA), placed on ice, and macerated with sterilized glass pestles separately for each sample. Cross-contamination was minimized by sanitizing surfaces and sterilizing all equipment and materials between specimen dissections, and filter tips were used to handle all liquids containing DNA. Total DNA extraction was performed using DNeasy Blood & Tissue kit (Qiagen). DNA purity and concentration were assessed by the NanoDrop spectrophotometer (Thermo Fisher Scientific, Wilmington, DE, USA). DNA quantity was normalized to 1 mg/mL across samples and split into 3 parts, 1 for Lazaro, 1 for metabarcoding, and 1 for MCA in qPCR Roche LightCycler® 480 Real-Time PCR System II (Roche Life Science, Penzberg, Bay., Germany).

### Preparation of the DNA samples for metabarcoding and Lazaro analyses

For Lazaro, the pertinent aliquots obtained from the previous step were normalized to 150 ng DNA/sample. For metabarcoding, a region of the 16S mitochondrial gene was amplified using the primer pair Ins16S_1short (forward 5′-TRRGACGAGAAGACCCTATA-3′ and reverse 5′-ACGCTGTTATCCCTAAGGTA-3′), which generates an amplicon of ∼190 bp [11], following the recommendation of using primer pairs that generate amplicons <200 bp for more degraded environmental samples, such as gut contents [33]. 16S barcode was chosen over COI for several reasons. Although the amount of arthropod 16S sequences in GenBank was considerably smaller (133,899 sequences, 2,630 families, 5,829 genera, and 48,711 species obtained from GenBank using the following search: arthropod[organism] AND 16S, release date 31 December 2018) than the COI sequences (2,570,787 sequences from 1,395 families, 10,394 genera, and 26,024 species obtained from GenBank using the following search: coi[Gene Name] AND arthropoda[Organism] AND “1900”[Publication Date] : “2018/12/31”[Publication Date]), it had higher taxonomic coverage. In addition, Clarke et al. [11] demonstrated better taxonomic coverage of 16S than COI and bias of COI to amplify more lepidopterans and dipterans, while failing to amplify other insect orders (e.g., hymenopterans). Last, according to Deagle et al. [10], Elbrecht et al. [34], and Sousa et al. [35], 16S has been preferentially used because 16S has some regions of more conserved sites across taxonomic groups, spanning sufficiently variable regions among taxa, resulting in more universal primers with equivalent taxonomic resolution than COI. Primers were not tagged to eliminate bias related to the tagging process [36], so an independent library was produced for each epigeal predator DNA gut sample. PCR reactions (0.2 μM primer pair) were performed in triplicate using Qiagen Multiplex PCR Master Mix and adding 1.28 μg/μL of bovine serum albumin to prevent PCR inhibition [37]. Cycling conditions were as follows: initial heat activation 15 min at 95°C, 40 cycles of 3-step cycling (denaturation 30 s at 94°C, annealing 90 s at 60°C, extension 90 s at 72°C), and final extension for 10 min at 72°C. Triplicates were pooled and purified using QIAquick PCR Purification Kit (Qiagen). Amplicons were quantified by NanoDrop (Thermo Fisher Scientific) and normalized in equimolar ratios across all the metabarcoding samples. We opted not to multiplex the 27 metabarcoding samples to keep the same coverage between metabarcoding and Lazaro methods.

### DNA sequencing

All Lazaro and enriched barcode samples were dried in a speed vacuum centrifuge. For the feeding bioassay samples, 20 samples were dried, which comprised 5 treatments (without feeding and 4 times after feeding) × 2 sexes × 2 methods (metabarcoding and Lazaro). For the field samples, 54 samples were dried, which comprised 27 for metabarcoding and 27 for Lazaro. The dried feeding bioassay and field samples were shipped simultaneously to the Roy J. Carver Biotechnology Center (University of Illinois at Urbana-Champaign, IL, USA) to construct KAPA Hyper libraries (Kapa Biosystems, Wilmington, MA, USA) with insert size 200–600 bp using unique dual indexes. Quality-checked samples were sequenced by Illumina HiSeq4000 (Illumina HiSeq 3000/HiSeq 4000 System, RRID:SCR_016386) (150 bp paired-end, 151 cycles, HiSeq 4000 sequencing kit version 1) in a single lane. The Brazilian license to access the genetic heritage was provided by CGEN/SISGEN A8E3D94. Sequence SRA access codes are presented in Supporting Information 1.

### Reference DNA databases and bioinformatic analysis

For metabarcoding, the reference database was constructed by extracting invertebrate 16S barcode regions from the European Nucleotide Sequence database (EMBL) (release 132; inv: invertebrate database/division; std: standard) using the ecoPCR program [38]. The EMBL is shared daily with GenBank (from USA) and DNA Data Bank of Japan databases [39]. In addition, 16S sequences for several species that were collected in the pitfall traps were determined and added to the other arthropod 16S sequences obtained from GenBank, resulting, after *in silico* PCR using ecoPCR and the Ins16S_1short primer, in a 16S amplicon database composed of 63,618 sequences for 39,397 species from 2,172 families. Prey detection analysis was performed using OBITools as in [26, 40,41]. The metabarcoding threshold for taxonomic assignment was 98% identity, and reads with count <100 were removed. Only “head” and “singleton” identifications were considered.

For the Lazaro reference database [28,29], we constructed a comprehensive arthropod mitochondrial DNA database by obtaining all sequences (partial or complete, Fasta format) available at the time at GenBank (n = 3,381, distributed in 2,779 species from 1,850 genera in 598 families). In addition, following the mitochondrial elucidation method described in Paula et al. [28,29] and briefly presented in Supporting Information 1, we provided mitochondrial sequences of 29 taxa (Supplementary Table S1) corresponding to the main potential prey co-occurring with the sampled epigeal predators in the experimental plots, including the predators under analysis (taxa and taxonomic determinations in Supplementary Table S2). For taxonomic prey identification, we used the Lazaro method [42], which is designed to detect and quantify species from degraded eDNA samples. Briefly, this method takes raw BLASTn (BLASTn, RRID:SCR_001598) output of hit matches, identifies the mismatches (or single-nucleotide polymorphisms) between the query and reference sequence, removes false mismatches (e.g., degenerate IUPAC nucleotide codes, e.g., R = A or G; Y = T or C; S = C or G), reanalyzes overlap length and percent identity, filters the best-hit matches with an overlap-identity threshold, eliminates singleton reads, and filters the reads mapping to coding regions of their respective reference mitogenome. The scripts are available in the GitHub repository [43]. The best overlap-identity threshold was determined using previous experimental data [42], and determined to be 100% identity in an overlap length of ≥130 bp (Supporting Information 1). Fastq files were generated and demultiplexed with the bcl2fastq (bcl2fastq, RRID:SCR_015058) v2.17.1.14 Conversion Software (Illumina). The quality assessment for each dataset was done using FastQC (FastQC, RRID:SCR_014583) (v.0.11.3) [44]. Low-quality sequences (Phred <30) and library index adaptors were trimmed by Fastqc-mcf (v.1.04.807) [45] and Cutadapt (cutadapt, RRID:SCR_011841) (v.1.9.1) [46]. Retained high-quality Fastq reads were converted to Fasta format by SeqTK (Seqtk, RRID:SCR_018927) (v1.2) [47].

### MCA confirmation of the field-detected prey

For the field samples, we performed MCA in Roche LightCycler® 480 Real-Time PCR System II to confirm the presence of the prey DNA detected by metabarcoding and Lazaro. The principle is based on the estimation of the melting temperature (Tm), which is the temperature at which 50% of the 2 strands of DNA dissociate, a property dependent on nucleotide composition and product length [48,49]. By monitoring denaturation of the PCR products with SYBR Green and fluorescence levels over a temperature gradient, it is possible to construct the melting curve [50,51]. The DNA source was the original DNA extracted from the gut contents of the predators. For 25 of the 30 species potentially detected as prey, we obtained specimens with confirmed taxonomy to determine a positive control reference Tm to distinguish TP and false-positive (FP) detections. Their DNA was extracted using the DNeasy Blood & Tissue kit (Qiagen). Species-specific primer pairs were designed as in Paula and Andow [52], nearly all in regions of the mitogenome (primer sequences at Supplementary Table S3), for all prey species detected by metabarcoding and Lazaro, using the program Primer 3 at Geneious v7.1.9 [53] and checked in NCBI/Primer-BLAST [54]. The cross-reactivity of these primers with related detected species is presented in Supplementary Figs S1–S4. The qPCR reactions (13 μL) were prepared using Maxima SYBR Green/ROX qPCR Master Mix (2×) (Thermo Fisher Scientific), 1.28 μg/μL of bovine serum albumin, and 10 ng of DNA per reaction and each specific primer pair at 0.3 μM. The amplifications were performed in 384-well plates with a Roche LightCycler® 480 Real-Time PCR System II using a 2-step cycling protocol (initial denaturation at 95°C for 10 min, ramp 4.4°C/s), and 40 cycles of denaturation at 95°C for 15 s (ramp 4.4°C/s) and annealing/extension at 60°C for 60 s (ramp 2.2°C/s), and a melt curve from 60°C to 95°C continuous (ramp 1°C/s) with 6 readings/°C. qPCR for each sample was performed in ≥3 technical replicates. No-template controls were included for every primer pair. Melt curves were constructed using the raw fluorescence data and diffQ in the library MBmca in *R* [55]. Positive prey detection and identification were considered if ≥2 technical replicates had -dF/dT more than 0.1 above background or if 1 technical replicate had -dF/dT more than 0.2 above background at the Tm expected for the prey. When there was no positive control, a sample was considered a TP if the 3 amplicon replicates had similar melting curves with the same sharp Tm. The presence of multiple peaks suggests that the PCR amplicons were heterogeneous and/or possibly mixed with chimeras or primer dimers.

### Statistical analysis

For each metabarcoding and Lazaro library, we have the number of species detected, the number of reads for each detected species, and for the field samples, independent confirmation of each detection by MCA. We used MCA to classify true positive (TP) and false positive (FP or type I error) detections and true negative (TN) and false-negative (FN or type II error) non-detections. We are considering the following categories: TP is detected prey DNA confirmed by MCA; FP is detected prey DNA not confirmed by MCA; TN is prey DNA not detected by MCA when the species was not detected by metabarcoding or Lazaro; and FN is prey DNA detected by MCA when not detected by metabarcoding or Lazaro. Prey species that were detected by MCA but were not detected by Lazaro, because they did not have a sequence in the Lazaro DNA reference database, were not considered FNs. For the mock community, we did not need MCA to determine TP, FP, and FN detections because the predator feeding history was known.

We calculated the theoretical limit of detection (LOD) of MCA for all of the species with positive controls by estimating the amount of whole organism template that could be detected at a *C_q_* = 40, and calculating the upper 95% confidence interval of the geometric mean of the estimates. In addition, we estimated the amplification efficiency of the MCA primers to ensure that it was high enough to amplify rare template sufficiently to detect. The limit of detection of MCA is quite low, but if there is only a small amount of prey DNA left in the gut, it is also possible that the prey sequence targeted by the species-specific primer pairs of MCA is absent, while other sequences are detected by metabarcoding and or Lazaro. If this were occurring, then for a given prey there should be a lower read count in both metabarcoding and Lazaro when the MCA is negative than when it is positive. We tested this with the prey species for which there were >3 TP and FP detections in the sample libraries. Number of reads were ln-transformed and analyzed by the Welch *t*-test for unequal variance using the Welch-Satterthwaite equation to calculate degrees of freedom.

To compare the performance of metabarcoding and Lazaro, for each library, we also estimated [56,57]:

- Sensitivity (or TP rate), which is the probability that a positive is detected: Sensitivity = TP/(TP + FN);
- Specificity (or TN rate), which is the probability that a negative is not detected: Specificity = TN/(TN + FP);
- False discovery rate (FDR), which is the probability that a detection is an FP: FDR = FP/(FP + TP);
- False omission rate (FOR), which is the probability that a non-detection is an FN: FOR = FN/(FN + TN);
- Accuracy, which is the probability that detections and non-detections are correct: Accuracy = (TP + TN)/(TP + TN + FP + FN).

Higher sensitivity, specificity, and accuracy and lower FDR and FOR are indicative of a better method. Using the aforementioned definitions, we tested the following hypotheses:

H_1_: Metabarcoding detects a higher number of TP prey species than Lazaro because the reference database is larger. We tested this by comparing the number of initial detections in a library and the proportion of TP detections after confirmation by MCA using a paired *t*-test with the 27 samples as independent observations, predicting that metabarcoding would have more TPs and a higher proportion of true detections;

H_2_: Metabarcoding is more prone to FP prey detections because of amplification bias and the larger reference database. We tested this by comparing the FDR and specificity, predicting that metabarcoding would have a higher FDR and a lower specificity;

H_3_: Lazaro is more prone to generate FNs because, lacking the amplification of rare prey DNA fragments, it would be less sensitive. We tested this by comparing the FOR and sensitivity, predicting that Lazaro would have a higher FOR and lower sensitivity;

H_4_: Lazaro enables prey detection with finer taxonomic resolution because of the larger reference targets (e.g., mitogenomes) and coverage, which would reduce ambiguity in species identifications. We tested this by comparing the taxonomic resolution of the final prey identifications.

H_5_: Number of reads for both metabarcoding and Lazaro are positively related to the probability of a TP across all prey species and to the relative qPCR template concentration for TP detections within prey species. We tested the first part of this hypothesis using logistic regression of the ln-transform of the number of reads on the binomial variate indicating TP detections by MCA versus FPs (logit link, binomial error) with Anova in the package car (type II Wald χ^2^) and glm in Base R. There were 109 observations for metabarcoding and 116 observations for Lazaro, and no significant overdispersion for either regression. We tested the second part of this hypothesis, i.e., the number of reads is related to the amount of prey DNA in a sample, using the estimated relative template concentration from the qPCR for prey species with ≥5 TP detections and variation in both variables of ≥0.5 order of magnitude. This was tested within prey species because amplification efficiency, baselines, and thresholds would be constant for the qPCR. There were 3 and 2 species tested for metabarcoding and Lazaro, respectively. We calculated the relative initial template concentration from the qPCR amplification curves using LinRegPCR (version 2017.1) with the estimated mean PCR efficiency for each primer pair [58]. Relative initial template concentrations were log_10_ transformed, number of reads was ln-transformed, and data were analyzed with Pearson correlation coefficients using the Fisher transformation to estimate *P*-values.

## Results and Discussion

### Prey detections from the mock community

After quality control, the 10 metabarcoding samples (predator guts or libraries) had a mean of 2,728,294 reads (8% coefficient of variation [CV]) and the 10 Lazaro samples had a mean of 5,355,167 reads (10% CV). None of the samples of the unfed control predators had any prey detected for either males or females for either metabarcoding or Lazaro. This indicates that extraneous DNA was unlikely to have contaminated the samples during and after the extraction process.

Only Lazaro detected prey species in the mock community (Table 1; Accuracy_female_ = 0.71, Accuracy_male_ = 0.43). For some prey species, only 1 sex of the predator detected prey and with few reads (n = 2). Although ecoPCR [38] theoretically returned amplicons for the target prey species (maximum number of mismatches allowed per primer: −e = 2 and using the # feature to ensure perfect matches in the last 2 nucleotides at the 3′-end), and the metabarcoding reference database contained their 16S sequences, none of the prey were detected (Accuracy_female_ = Accuracy_male_ = 0), possibly owing to the mismatches in at least 1 primer of the pair (Supplementary Fig. S5) and preferential amplification of the more abundant predator DNA in the samples. Only the predator was detected by metabarcoding. This illustrates that the insufficient primer universality among taxa can preclude the detection (reduced sensitivity) of expected prey species and that the use of multiple barcodes may be preferable.

**Table 1:** : Number of reads detected for the mock community by metabarcoding (16S barcode) and Lazaro using the harlequin *Harmonia axyridis* (unfed for 48 h after adult emergence) as predator and 7 prey species, consumed within 1 hour. Gut contents of the predators were analyzed within 6 hours after feeding on all 7 prey species. The threshold used for Lazaro was 100% identity in a minimum overlap of 130 bp and for metabarcoding was 98% identity for an amplicon between 180-230 bp. Mb: metabarcoding; L: Lazaro. The number of reads detected for the control predators: female 114,033 by metabarcoding and 18,984 by Lazaro; male 130,134 by metabarcoding and 35,340 by Lazaro

		**Predator**								**Prey**							
**Predator sex**	**Time (h) after feeding**	***Harmonia axyridis***		***Acrythosiphon pisum***		***Aphis craccivora***		***Aphis glycines***		***Aphis gossypii***		***Myzus persicae***		***Cycloneda munda***		***Plutella xylostella***	
		**Mb**	**L**	**Mb**	**L**	**Mb**	**L**	**Mb**	**L**	**Mb**	**L**	**Mb**	**L**	**Mb**	**L**	**Mb**	**L**
Female	6	128,773±11,715	40,498±9,557	0	16	0	26	0	2	0	2	0	0	0	6	0	0
Male	6	451,571±371,434	43,452±9,444	0	10	0	4	0	0	0	0	0	10	0	0	0	0

Neither metabarcoding nor Lazaro made any FP detections, despite the use of comprehensive reference databases, which increases the likelihood of FP detections. In summary, both metabarcoding and Lazaro generated FNs, with more FN and fewer TP detections by metabarcoding than Lazaro. These results suggest that neither method on its own will detect all of the prey in a predator gut sample, but that Lazaro may sometimes be more accurate.

### Prey detections from field sampled predators

**Figure 1:**
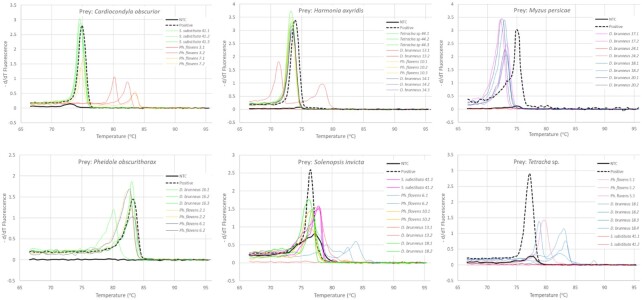
: Confirmation of prey detection by Melting Curve Analysis (MCA) in qPCR with positive controls and NTCs (no template controls). The graphs represent a melting curve for some prey detected by metabarcoding, Lazaro or both. Predator samples are informed in the side legend. Green, yellow, and gray curves indicate positive identification of the respective prey, and red, magenta, and blue curves indicate negative identifications. Sample numbers designate library and technical replicate.

After quality control, the 27 metabarcoding samples (predator guts or libraries) had a mean of 2,964,430 reads (12% CV) and the 27 Lazaro samples had a mean of 5,504,578 reads (14% CV). The list of the prey species detected for each method before confirmation by MCA is in Table 2. The presence of the DNA of each prey species in a predator gut sample was confirmed by MCA. Examples of the various positive and negative prey confirmations by MCA are illustrated in Fig. 1. The differentiation of a true and false prey detection was performed by observing the presence or absence of the sample Tm peak corresponding to the Tm of the positive control. For example, *Solenopsis invicta* TP control had a Tm of 76.5°C, and FP detections had Tm at 77.9, 79.2, 82.6, and 84.0°C. The theoretical LODs for detection by qPCR amplification were <1 pg of whole-organism DNA/technical replicate for 24 of the 30 detected prey species, and for 21 of these species it was <0.1 pg of whole-organism DNA/technical replicate (Supplementary Table S4). As the DNA templates for MCA are only a small proportion of the whole-organism DNA, the LODs indicate that MCA was very sensitive and unlikely to return an FN for the majority of prey species examined. The amplification efficiency varied from 1.893 to 1.997 for all of the species-specific primer pairs, which should result in sufficient amplicons for detection by MCA even when the template is rare. Nevertheless, 3 of the 30 detected prey species had higher LODs, which might have resulted in some FNs: *Selenophorus alternans* (LOD = 1.652 pg/technical replicate), *Euschistus heros* (LOD = 3.245 pg/technical replicate), and *Cardiocondyla obscurior* (LOD = 22.59 pg/technical replicate). Although unlikely, MCA could also give an FN when the prey DNA was present but so scarce that there was no MCA template in the sample. In this case, we reasoned that if MCA returned FN detections then the number of reads associated with MCA negatives should be smaller than the number of reads for MCA positives within a prey species. However, only 1 species (*Pheidole tristis* detected by Lazaro) had fewer reads associated with negative than with positive MCA detections (Supplementary Fig. S6), indicating that FN MCA detections were generally not a problem.

**Table 2:** : Species detected as prey of epigeal arthropod predators by metabarcoding, Lazaro or both in at least 1 of 27 libraries before confirmation by Melting Curve Analysis (MCA) in qPCR

Order	Species (Family)	Detection method(s)	No. reads	No. libraries	Predator
Annelida	*Phascolosoma esculenta* (Phascolosomatidae)	Metabarcoding	21,938	13	*Dorymyrmex brunneus, Tetracha* sp.
Coleoptera	*Anthonomus grandis* (Curculionidae)^1^	Both	946,283	27	*Pheidole flavens, Dorymyrmex brunneus, Solenopsis substituta, Tetracha* sp., *Euborellia annulipes*
	*Eriopis connexa* (Coccinellidae)	Both	2,706	5	*Dorymyrmex brunneus, Solenopsis substituta, Tetracha* sp., *Euborellia annulipes*
	*Harmonia axyridis* (Coccinellidae)^1^	Both	12,800,160	27	*Pheidole flavens, Dorymyrmex brunneus, Solenopsis substituta, Tetracha* sp., *Euborellia annulipes*
	*Selenophorus alternans* (Carabidae)	Both	1,639,746	9	*Pheidole flavens, Dorymyrmex brunneus, Solenopsis substituta, Tetracha* sp.
	*Tetracha brasiliensis* (Carabidae)	Lazaro	2	1	*Solenopsis substituta*
Dermaptera	*Doru luteipes* (Forficulidae)^1^	Both	1,808	7	*Pheidole flavens, Dorymyrmex brunneus, Euborellia annulipes*
	*Euborellia annulipes* (Anisolabididae)^1^	Both	41,492	12	*Pheidole flavens, Dorymyrmex brunneus, Solenopsis substituta, Tetracha* sp.
Diptera	*Strongygaster triangulifera* (Tachinidae)	Both	62	2	*Tetracha* sp., *Euborellia annulipes*
Hemiptera	*Chinavia impicticornes* (Pentatomidae)^1^	Both	24,621	10	*Dorymyrmex brunneus, Solenopsis substituta, Tetracha* sp., *Euborellia annulipes*
	*Euschistus heros* (Pentatomidae)^1^	Both	267,209	5	*Dorymyrmex brunneus, Tetracha* sp.
	*Mahanarva spectabilis* (Cercopidae)^1^	Lazaro	12	2	*Solenopsis substituta, Tetracha* sp.
	*Neomegalotomus parvus*	Metabarcoding	365	1	*Tetracha* sp.
	*Myzus persicae* (Aphididae)	Metabarcoding	1,696	2	*Dorymyrmex brunneus*
	*Planicephalus flavicosta* (Cicadellidae)	Metabarcoding	367	1	*Dorymyrmex brunneus*
Hymenoptera	*Atta sextans* (Formicidae)	Both	2,291	6	*Dorymyrmex brunneus*
	*Brachymyrmex patagonicus* (Formicidae)	Lazaro	24	11	*Pheidole flavens, Dorymyrmex brunneus, Tetracha* sp., *Euborellia annulipes*
	*Cardiocondyla obscurior* (Formicidae)^1^	Both	5,576	3	*Pheidole flavens, Solenopsis substituta*
	*Dorymyrmex brunneus* (Formicidae)^1^	Both	3,829	15	*Pheidole flavens, Solenopsis substituta, Tetracha* sp., *Euborellia annulipes*
	*Pheidole flavens* (Formicidae)^1^	Both	244,820	15	*Dorymyrmex brunneus, Solenopsis substituta, Tetracha* sp., *Euborellia annulipes*
	*Pheidole obscurithorax* (Formicidae)^1^	Both	36	4	*Pheidole flavens, Dorymyrmex brunneus*
	*Pheidole oxyops* (Formicidae)^1^	Both	9,015,220	27	*Pheidole flavens, Dorymyrmex brunneus, Solenopsis substituta, Tetracha* sp., *Euborellia annulipes*
	*Pheidole tristis* (Formicidae)^1^	Both	2,795,078	27	*Pheidole flavens, Dorymyrmex brunneus, Solenopsis substituta, Tetracha* sp., *Euborellia annulipes*
	*Solenopsis richteri* (Formicidae)	Metabarcoding	75,556	1	*Solenopsis substituta*
	*Solenopsis substituta* (Formicidae)	Both	608	9	*Pheidole flavens, Dorymyrmex brunneus*
Isoptera	*Syntermes spinosus* (Termitidae)^1^	Metabarcoding	897	1	*Dorymyrmex brunneus*
Lepidoptera	*Chrysodeixis includens* (Noctuidae)^1^	Both	45	1	*Tetracha* sp.
	*Glena unipennaria* (Geometridae)	Both	280	1	*Tetracha* sp.
	*Spodoptera frugiperda* (Noctuidae)^1^	Metabarcoding	8,448	11	*Dorymyrmex brunneus, Tetracha* sp.
Orthoptera	*Gryllus argentinus* (Gryllidae)	Metabarcoding	32	1	*Dorymyrmex brunneus*

1 Species with detection confirmed by MCA in ≥1 library. All these species are likely to occur in the sampling area/period.

Initially, 30 prey were identified, all to species level, by both methods prior to confirmation by MCA (Table 3 and Supplementary Table S5). They were 6 species of Heteroptera, 10 Hymenoptera (Formicidae), 5 Coleoptera, 3 Lepidoptera, 2 Dermaptera, and 1 species of Diptera, Orthoptera, Isoptera, and Annelida. Metabarcoding and Lazaro initially detected a similar number of prey species (26 [87%] and 21 [81%], respectively, with 16 species in common), but metabarcoding resulted in more species detections per sample than Lazaro (7.85 ± 0.63 vs 6.78 ± 0.42, respectively, *P* = 0.0479; Table 3). There were 212 prey detections (i.e., some prey species were detected in more than 1 sample) in the metabarcoding samples, with ln-number of reads averaging 7.66 (range 0–14.44), and 183 prey detections in the Lazaro samples, with ln-number of reads averaging 2.90 (range 0.69–8.39). Of 30 prey species initially detected, 16 (53%) were confirmed by MCA as prey of the 5 epigeal predators (14 species by metabarcoding and 13 by Lazaro, with 11 species in common, i.e., 69% of confirmed prey species; Supplementary Table S5). Ten of the 14 species not confirmed by MCA were FP detections and 3 species (*Atta sextans, Neomegalotomus parvus*, and *Strongygaster triangulifera*) were not tested (Supplementary Table S5). Of the 11 FP species, 6 were detected by Lazaro in 16 prey detection instances (8.7% of initial detections), and 7 were detected by metabarcoding in 21 prey detection instances (9.9% of initial detections). Most of these FPs were not amplified by MCA or the replicates did not have a consistent Tm. In a few cases the replicates had a consistent Tm but at the wrong temperature. For example, all of the MCA replicates for the false detections of *M. persicae* gave a consistent signal with a sharp Tm peak, but the peak was >2°C lower than the Tm of the TP control (Fig. 1). This kind of FP might have resulted from taxonomic overclassification (i.e., erroneous detection of related species when the true species is absent in the reference database) [59]. These would give FP species identifications because they were identified beyond the resolution limitation of a reference database. Indeed, the metabarcode amplicon for the false detections of *M. persicae* also had high similarity (≥98%) with several other species of Macrosiphonini, the aphidid tribe of *M. persicae* (BLASTn search, Supporting Information 1) . Hence, some FPs might be a TP prey with a false species determination.

**Table 3:** : Number of prey species identified by metabarcoding and Lazaro before confirmation, and proportion verified by Melting Curve Analysis (MCA) in qPCR

Predator Species	Original			Proportion verified by MCA		
	Mb	L	Both	Mb	L	Both
*Pheidole flavens*	6	6	5	0.40	0.33	0.40
*Pheidole flavens*	5	5	4	0.50	0.50	0.67
*Pheidole flavens*	6	6	5	0.20	0.25	0.25
*Pheidole flavens*	6	5	5	0.40	0.40	0.40
*Pheidole flavens*	5	6	4	0.33	0.33	0.33
*Pheidole flavens*	5	6	5	0.60	0.50	0.60
*Pheidole flavens*	4	6	4	0.33	0.25	0.33
*Pheidole flavens*	4	5	4	0.00	0.00	0.00
*Pheidole flavens*	5	7	4	0.67	0.50	0.67
*Pheidole flavens*	4	7	4	0.50	0.33	0.50
*Pheidole flavens*	4	6	4	0.33	0.20	0.33
*Pheidole flavens*	5	6	3	0.40	0.40	0.67
*Dolymyrmex brunneus*	9	6	6	0.29	0.25	0.33
*Dolymyrmex brunneus*	10	7	5	0.20	0.20	0.33
*Dolymyrmex brunneus*	8	4	4	0.20	0.33	0.33
*Dolymyrmex brunneus*	6	9	5	0.25	0.33	0.33
*Dolymyrmex brunneus*	10	5	5	0.25	0.25	0.25
*Dolymyrmex brunneus*	11	6	5	0.19	0.33	0.33
*Dolymyrmex brunneus*	12	8	6	0.33	0.50	0.67
*Dolymyrmex brunneus*	10	8	5	0.00	0.00	0.00
*Dolymyrmex brunneus*	10	6	5	0.43	0.60	0.75
*Dolymyrmex brunneus*	8	8	5	0.00	0.00	0.00
*Dolymyrmex brunneus*	8	6	5	0.40	0.50	0.67
*Dolymyrmex brunneus*	13	6	5	0.13	0.33	0.33
*Solenopsis substituta*	12	11	8	0.43	0.50	0.50
*Tetracha* sp.	16	15	10	0.45	0.55	0.50
*Euborellia annulipes*	10	7	6	0.33	0.33	0.33
**Mean**	7.85	6.78	5.04	0.32	0.33	0.40
**Standard Error**	0.63	0.42	0.26	0.03	0.03	0.04

L: Lazaro; Mb: metabarcoding.

Another possible reason for FP detections is contamination of the samples after DNA extraction. This could occur during any of the post-extraction procedures, including library preparation and sequencing. If such contamination had occurred, either or both metabarcoding and Lazaro might detect the contaminant with substantial number of reads, but MCA would not because the contaminant would not be in the original DNA extracted sample. For example, many of the FP detections of the coleopterans *Anthonomus grandis* (19 samples, 47,626 metabarcoding reads, 142 Lazaro reads; Supplementary Table S5) and *H. axyridis* (8,919,830 metabarcoding reads, 18 samples, 1,190 Lazaro reads; Supplementary Table S5) had these characteristics and may be post-extraction contaminants. The presence of *A. grandis* at the experimental site was unlikely because it is a specific cotton herbivore and none of the experimental plots had cotton; however, this species is mass-reared in a nearby laboratory, which may have been the source of contamination. FP detections can also occur when the prey species is closely related to the predator. An example was the false detection by metabarcoding of *Solenopsis richteri* in the gut content of the predator *Solenopsis substituta* (Supplementary Table S5).

Regarding FN prey detection, there were 11 FNs for metabarcoding and 11 FNs for Lazaro (Table 4). For example, *Mahanarva spectabilis* was not detected as prey by metabarcoding in 3 samples, and *Spodoptera frugiperda* was not detected as prey by Lazaro in 2 samples (Supplementary Table S5), but they were detected by MCA. FNs could have been generated for rare prey in the samples with a large number of pooled individual predators (Table 5). For example, for the Lazaro samples, coverage ranged from 20,000 to 120,000 reads/predator, and the number of detected prey reads was only 1.3–29.2/predator. Thus, rare prey may be missed (FN) in the Lazaro samples. The metabarcoding samples had coverage ranging from 11,000 to 64,000 amplicons/predator, and the number of detected prey amplicons ranged from 114 to 40,000/predator. Thus, rare prey may have been missed because of the large number of individuals in a sample. However, because the 3 ant species are known to recruit large number of individuals to harvest prey, rare prey are likely to occur in multiple individuals and may be unlikely to be missed. Moreover, if FNs were primarily related to missing rare prey, then the FOR should be negatively correlated with coverage or prey detection. This was not observed (Table 5 and Supplementary Table S6); hence, while some rare prey may have been missed, they were equally likely to have been missed by both metabarcoding and Lazaro. Finally, it is also possible that FP and FN detections can occur because the taxonomy of the reference genetic material at GenBank was incorrect.

**Table 4:** : False negative (FN) and false positive (FP) species detected by metabarcoding, Lazaro, or both

	**Metabarcoding**	**Lazaro**	**Both**
False negatives	*Pheidole obscurithorax*	*Syntermes spinosus* ^1^	*Cardiocondyla obscurior*
			*Chrysodeixis includens*
			*Mahanarva spectabilis*
			*Solenopsis richteri*
			*Spodoptera frugiperda*
False positives	*Euschistus heros*	*Brachymyrmex patagonicus*	*Anthonomus grandis*
	*Gryllus argentinus* ^1^	*Pheidole obscurithorax*	*Cardiocondyla obscurior*
	*Myzus persicae*	*Solenopsis substituta*	*Doru luteipes*
	*Phascolosoma esculenta* ^1^	*Tetracha* sp.	*Dorymyrmex brunneus*
	*Planicephalus flavicosta* ^1^		*Eriopis connexa*
	*Solenopsis richteri*		*Glena unipennaria*
	*Spodoptera frugiperda*		*Harmonia axyridis*
			*Pheidole oxyops*
			*Pheidole tristis*
			*Selenophorus alternans*

1 Species that did not have a mitogenome deposited at the GenBank.

**Table 5:** : Number of reads detected per predator in the total sample and for detected prey, and false omission rate for each predator species

			Total sample		Detected prey		False omission rate	
Predator species	No. of samples	Predators/sample	Metabarcoding reads/predator	Lazaro reads/predator	Metabarcoding reads/predator	Lazaro reads/predator	Metabarcoding	Lazaro
*Dorymyrmex brunneus*	12	100	29,644	55,046	11,797	4.2	0.058	0.047
*Euborellia annulipes*	1	46	64,444	119,665	114	9.1	0.250	0.250
*Pheidole flavens*	12	200	14,822	27,523	4,867	10.2	0.044	0.058
*Solenopsis substituta*	1	273	10,859	20,163	317	1.3	0.286	0.167
*Tetracha* sp.	1	49	60,499	112,338	39,600	29.2	0.200	0.167

**Figure 2:**
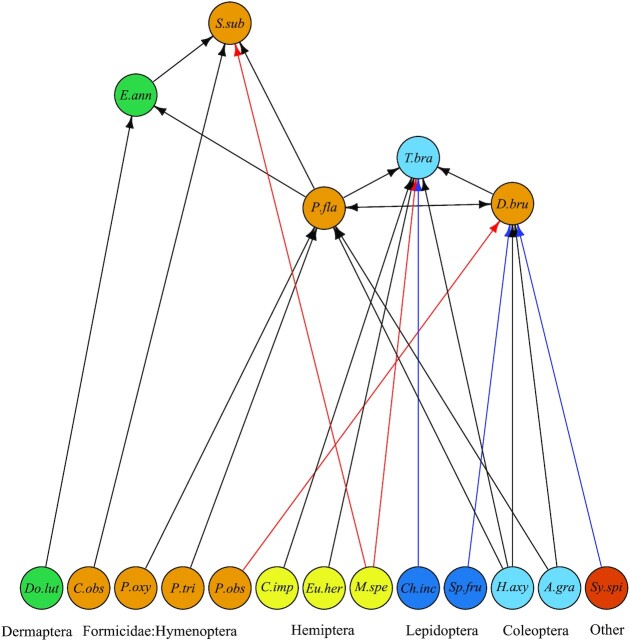
: Qualitative food web of the 5 epigeal predators (top of figure) detected by metabarcoding only (blue links), Lazaro only (red links), or both (black links) and confirmed by melting curve analysis (MCA) in qPCR. Predation is indicated by the arrow direction. Height of a species indicates its relative trophic level . Predator species are *E.ann* = *Euborellia annulipes; S.sub* = *Solenopsis substituta; P.fla* = *Pheidole flavens; T.bra* = *Tetracha brasilensis; D.bru* = *Dorymyrmex brunneus*. Extra- or intraguild prey are *Do.lut* = *Doru luteipes; C.obs* = *Cardiocondyla obscurior; P.oxy* = *Pheidole oxyops; P.tri* = *Pheidole tristis; P.obs* = *Pheidole obscurithorax; C.imp* = *Chinavia impicticornes; Eu.her* = *Euschistus heros; M.spe* = *Mahanarva spectabilis; Ch.inc* = *Chrysodeixis includens; Sp.fru* = *Spodoptera frugiperda; H.axy* = *Harmonia axyridis; A.gra* = *Anthonomus grandis; Sy.spi* = *Syntermes spinosus*.

Species identifications have been demonstrated to differ when using different DNA extraction protocols, DNA polymerases, amplification parameters, reference databases or barcodes, and even when using different primers from the same barcode [9,13, 60–63]. The results from our mock community also showed that different DNA-based detection methods differed in the species identified. So, the incongruent prey species detection between metabarcoding and Lazaro may not be unusual and additional possible reasons are discussed below. Nonetheless, the underlying consequence is that the ecological inferences are likely to be affected by the prey detection method used as the predator food webs would have different structures (Fig. 2). Specifically, the food webs of 3 of the predators (*Ph. flavens, T. brasiliensis*and*D. brunneus*) would be different using only metabarcoding or Lazaro. These results highlight the need for precaution when comparing the data between eDNA studies to enable robust ecological comparisons [64,65]. Indeed, because the 2 methods appear to be partially complementary, using both may provide more robust results.

Operational taxonomic unit analysis could be conducted on the reads that did not match satisfactorily with any species in the DNA reference databases to complement the prey diversity analysis. The number of “unassigned” reads was fairly high in both methods but, not surprisingly, more prominent in Lazaro. The percent of reads of confirmed prey detections across all 27 predator samples was 45% for metabarcoding and <1% for Lazaro. The majority of the unassigned reads were related to the predator DNA (e.g., nuclear DNA), even though we reduced the amount of predator biomass by gut dissection or gaster removal (for ants). Another part of the unassigned reads could be related to predator symbionts or parasites or other exogenous species that were not present in the reference databases used in this work. Although high, the 99% of unassigned reads for Lazaro is not unexpected for 2 reasons: we only worked with arthropod mitochondrial reads and the predator reads were not counted as assigned. It is known that the proportion of mitochondrial reads obtained in next-generation sequencing of whole macerated organisms or tissues without *in vitro* mitochondrial enrichment is only ∼1% [19,29]. The predator mitochondrial reads should not be considered “unassigned” reads in the strict sense; nevertheless they were not included in the assigned reads because there is no means to differentiate the predator reads from reads of a cannibalized conspecific. In a similar way, the prevalence of 55% of unassigned reads for the metabarcoding data was not unexpected because no specific predator-blocking primers were used to preclude or minimize amplification of the predator template. Similar to Lazaro, most of the unassigned reads were from predator 16S amplicons. Our choice to not use specific predator-blocking primers was based on Piñol et al. [66], who demonstrated that predator-blocking primers may co-block the amplification of prey species closely related to the predator, which is critical when analyzing diet composition of arthropod generalist predators.

### Metabarcoding versus Lazaro

To compare which method, metabarcoding or Lazaro, resulted in better prey determination, we evaluated the sensitivity, specificity, FDR, FOR, and accuracy of prey determination for the field-sampled predators and accuracy of prey determination for the mock community. In addition, for the field-sampled predators we determined the relation between the number of reads for a prey and the amount of prey DNA in the samples (feeding bioassay controls were not analyzed this way because metabarcoding did not detect prey reads). Specifically, we tested the 5 hypotheses discussed below.

*H_1_: Metabarcoding detects more TP prey species than Lazaro*. Contrary to this hypothesis, in the field-sampled predators, metabarcoding and Lazaro had a similar number of confirmed prey per sample (1.81 ± 0.24 and 1.85 ± 0.23, respectively, *P* = 0.6632) and of the initial prey tested by MCA, the same FDR (0.68 ± 0.033 vs 0.67 ± 0.031, respectively; *P* = 0.3929, Table 6). The rejection of H_1_ was corroborated by the results from the mock community for which no TPs were detected by metabarcoding.

**Table 6:** : Sensitivity, specificity, false discovery rate, false omission rate, and accuracy for prey determinations in field-collected predators by metabarcoding and Lazaro, with paired *t*-test and *P*-value

Parameter	Sensitivity	Specificity	False discovery rate	False omission rate	Accuracy
Metabarcoding (95% CI)	0.806 (0.059)	0.577 (0.026)	0.683 (0.033)	0.073 (0.019)	0.622 (0.024)
Lazaro (95% CI)	0.814 (0.059)	0.618 (0.022)	0.666 (0.031)	0.068 (0.019)	0.663 (0.020)
*t*_26_	0.2726	1.1514	−0.8689	−0.4596	1.3881
*P*-value	0.7874	0.2601	0.3929	0.6496	0.1769

TP detections might be increased for metabarcoding by sequencing PCR replicates separately for each barcode per sample [67], using a multi-level assignment approach [68] or pre-testing the metabarcode primer to ensure amplification of expected prey and reduced amplification of the predator [69]. This last approach may hamper the detection of prey species closely related to the predator. While there are a number of publications showing that the use of multiple metabarcode primers increases the diversity of prey detection (e.g., [40]), they typically do not evaluate whether false prey detection also increases, as might be expected. In addition, there is no guarantee that the use of multiple primers will always improve metabarcoding performance. To illustrate this point, we show the putative mismatches of the 16S barcode primer pair that we used and 3 commonly used COI barcode primer pairs (LCO1490 and HCO2198 [70]; mICOIintF and jgHCO2198 [6]; UniMiniBar [60]) with the prey species used in the mock community and detected in the field samples (Supplementary Fig. S5). LCO1490/HCO2198 and UniMiniBar had a higher number of template mismatches than the 16S primers and probably would have detected fewer prey species than the 16S primer pair. The forward primer of the other COI primer pair had mismatches in the critical last 3 bases of the 3′-end with all 7 prey species in the mock community and in 8 of 29 species detected in the field samples (28%).

TP detections might be increased for Lazaro by using multiple reference databases. In this work, we used only a mitogenome reference database, but it is also possible to use rDNA and symbionts [28, 29] and even unassembled reads [24] in a reference database. These databases can be constructed and used at any time after sequencing, unlike metabarcoding.

*H_2_: Metabarcoding is more prone to FP prey detections than Lazaro*. Both the FDR and the specificity (0.58 ± 0.026 versus 0.62 ± 0.022, respectively; *P* = 0.2601, Table 6) were similar for metabarcoding and Lazaro, so H_2_ was rejected. While it was true that metabarcoding detected on average 350 times more true prey reads than Lazaro (Supplementary Table S5), this did not convert into a higher detection of TP or a significantly higher rate of FP prey detections compared to Lazaro.

*H_3_: Lazaro is more prone to have FNs than metabarcoding*. For the field-sampled predators, FOR for Lazaro was similar to that for metabarcoding (0.068 ± 0.019 versus 0.073 ± 0.019, respectively; *P* = 0.649; Table 6). Similarly, Lazaro did not have lower sensitivity than metabarcoding (0.814 ± 0.059 versus 0.806 ± 0.059, respectively; *P* = 0.7874; Table 6), so H_3_ was rejected. The rejection of H_3_ was corroborated by the results from the mock community. A factor that may have contributed to FN detections in some predators is that prey DNA is in an advanced state of degradation, precluding PCR amplification (in the case of metabarcoding) or being excluded by size selection during library construction, but still possible to be detected by MCA in the original sample DNA because of the smaller length of the target amplicon (100–200 bp, Supplementary Table S3). In the case of metabarcoding, it could also be related to insufficient complementarity between template and metabarcoding primers, precluding the representation of a prey species or taxonomic group in the sample, or preferential amplification of the more common predator DNA, resulting in poor amplification of prey DNA. Realistically, it is likely that the number of FNs might be even higher, because we could not check all species co-occurring in the sample area.

With H_1_, H_2_, and H_3_ rejected, it follows that metabarcoding and Lazaro had similar accuracy in prey detection (0.62 ± 0.024 versus 0.66 ± 0.020, respectively; *P* = 0.1769; Table 6). This differs from the results from the mock community and in Srivathsan et al. [26]. The accuracy in the mock community was 0 for metabarcoding versus 0.43–0.71 for Lazaro (log-linear model *g*^2^ = 9.36, *P* = 0.0022). Srivathsan et al. [26] compared metabarcoding and metagenomics (using BLASTn) to identify diet composition by fecal analysis (host plant chloroplasts) of 2 red-shanked doucs langurs (*Pygathrix nemaeus*) fed with a known diet. While metabarcoding detected 34% of the diet composition, metagenomics detected 50% of the known diet plus an unexpected species that was later confirmed to be in the diet.

*H_4_: Lazaro enables prey detection with finer taxonomic resolution than metabarcoding*. For the field-sampled predators, all confirmed species identifications were at the species level for both methods. Thus, in our field samples, taxonomic resolution was the same, and H_4_ was rejected.

*H_5_: Number of reads for both methods are positively related to the probability of a TP detection and to the relative qPCR template concentration for TPs*. Logistic regression showed that the probability of the TP was not related to the number of reads for metabarcoding (regression coefficient = 0.11±0.07, χ^2^ = 2.52, *P* = 0.1122) but was highly positively related to the number of reads for Lazaro (regression coefficient = 0.49±0.12, χ^2^ = 15.87, *P* = 6.798E−5). The tests determined whether the number of prey reads was correlated with the amount of prey in the gut of the predator, as measured by qPCR (Table 7). For Lazaro, H_5_ was accepted because there was a positive correlation between the number of reads and the relative qPCR template concentration in the samples for both species that could be analyzed. However, for metabarcoding, H_5_ was rejected for 3 of the 4 species tested because there was no correlation between the number of reads and the relative template concentration for these species. The quantitative interpretation of number of reads from the metabarcoding results has been controversial [71–76], and our results provide some support for the argument that the number of metabarcoding reads is an unreliable predictor of the DNA quantity in a sample but the number of Lazaro reads might be a good predictor.

**Table 7:** : Pearson correlations between relative initial qPCR template concentration and ln number of reads for TP detections

	***r***	***z*-score**	***P***
**Metabarcoding**			
*Harmonia axyridis*	−0.306	−0.893	0.3718
*Pheidole tristis*	0.865	1.887	0.0592
*Pheidole flavens*	−0.270	−0.920	0.3577
*Spodoptera frugiperda*	0.305	0.419	0.6752
**Lazaro**			
*Harmonia axyridis*	0.605	1.985	0.0472
*Pheidole tristis*	0.962	2.027	0.0427

In terms of cost, metabarcoding has the potential to cost roughly half that of Lazaro. In this study, we chose not to multiplex the 27 samples for metabarcoding analysis to keep the sequencing depth per sample similar between methods. The costs that were the same for both methods were sample preparation (USD10), total DNA extractions (USD100), library construction (USD85.50/each), and HiSeq4000 sequencing lane (USD4,310). For metabarcoding, there were additional costs for primer synthesis, PCR reactions, purification and quantifications for each sample, which were estimated to be USD160 total. If we had multiplexed the 27 purified sample amplicons in 1 library, the total cost of metabarcoding would have been USD2,510.50. For the Lazaro method, samples cannot be multiplexed and the total cost was USD4,573.50.

## Conclusions

Metabarcoding and Lazaro identified a range of prey species that were preyed upon by arthropod epigeal predators, but they were partially complementary methods sharing 87% of TP detections. Both methods crucially depended on the comprehensiveness of their respective DNA reference databases, which for metabarcoding was undeniably larger. Even so, Lazaro determined prey with similar specificity, sensitivity, FDR, FOR, accuracy, and taxonomic resolution as metabarcoding. The use of multiple barcode primers in the metabarcoding analysis could render higher sensitivity, although it could also increase FP detections (reduce specificity) as each primer carries its own associated bias. One may prefer Lazaro because it preserves qualitatively and quantitatively the original sample DNA community, enabling further search for other targets (e.g., host plants, symbionts, parasites) using any other DNA reference database(s), while for metabarcoding prey detection is constrained by the initial chosen barcodes. In addition, for Lazaro, the number of reads is associated with the quantity of prey DNA in the gut of the predators, enabling broader ecological inferences. However, one may prefer metabarcoding as it remains less expensive than Lazaro for processing a large set of samples because they can be multiplexed in a library.

## Data Availability

Library sequencing datasets were deposited at GenBank, and their SRA access codes are in Supporting Information 1. Supporting data and materials are available in the *GigaDB* database [43].

## Additional Files

**Supporting Information 1**. Contains mtDNA elucidation, SRA access codes, Lazaro bioinformatic workflow, MCA tests of cross-reactivity of primers, BLASTn alignments of species related to *Myzus persicae*, Figures S1-S6.

**Supporting Information 2**.Contains Tables S1-S6.

**Supplementary Figure S1**. Cross-reactivity of primers designed to detect two species of Carabidae against known positive control samples.

**Supplementary Figure S2**. Cross-reactivity of primers designed to detect *Chinavia* spp. against known positive control samples.

**Supplementary Figure S3**. Cross-reactivity of primers designed to detect lepidopteran species against known positive control samples.

**Supplementary Figure S4**. Cross-reactivity of primers designed to detect ant species against known positive control samples.

**Supplementary Figure S5**. Annealing of the some metabarcoding primers in the prey species used in the mock community and detected by metabarcoding, before MCA validation.

**Supplementary Figure S6**. Ratio of true to false positive reads for prey species detected by metabarcoding and Lazaro.

**Supplementary Table S1**. Mitochondrial DNA elucidated and annotated to add to the Lazaro reference database for prey identification.

**Supplementary Table S2**. Source of true positive prey samples for the Melting Curve Analysis (MCA) in qPCR.

**Supplementary Table S3**. Species specific primers (5’>3’) used for MCA-qPCR validation of the prey detected by metabarcoding or Lazaro.

**Supplementary Table S4**. Theoretical limit of detection for MCA primers as picograms of whole organism DNA/technical replicate.

**Supplementary Table S5**. Original read number and result of MCA-qPCR confirmation on the detection of prey in 27 libraries of epigeal predators.

**Supplementary Table S6**. Pearson correlations, Fisher z-score transformations, and *P*-values for the relation between predator false ommision rates and reads (or amplicons per predator; or verified prey for the field collected predators).

giac020_GIGA-D-21-00303_Original_Submission

giac020_GIGA-D-21-00303_Revision_1

giac020_Response_to_Reviewer_Comments_Revision_1

giac020_Reviewer_1_Report_Original_Submission Tuomas KankaanpÃ¤Ã¤, Ph.D. -- 10/22/2021 Reviewed

giac020_Reviewer_2_Report_Original_Submission Jordan Patrick Cuff, BSc MRes PhD -- 10/25/2021 Reviewed

giac020_Reviewer_2_Report_Revision_1 Jordan Patrick Cuff, BSc MRes PhD -- 12/9/2021 Reviewed

giac020_Supplemental_Files

## Abbreviations

BLAST: Basic Local Alignment Search Tool; bp: base pairs; CV: coefficient of variation; eDNA: environmental DNA; FDR: false discovery rate; FN: false negative; FOR: false omission rate; FP: false positive; IUPAC: International Union of Pure and Applied Chemistry; LOD: limit of detection; MCA: melt curve analysis; NCBI: National Center for Biotechnology Information; SRA: Sequence Read Archive; TN: true negative; TP: true positive.

## Competing Interests

The authors declare that they have no competing interests.

## Funding

This work was funded by the United States Department of Agriculture-National Institute of Food and Agriculture (USDA-NIFA) grant number 2016–67030-24950.

## Authors' Contributions

Design of study: D.P.P., D.A.A., R.M.P., M.R.B.

Collection and preparation of samples: S.K.A.B., R.M.P., D.P.P.

Data analyses (bioinformatic, qPCR, statistics): R.C.T., D.P.P., D.A.A.

Writing of the manuscript: D.P.P., D.A.A., R.C.T., R.M.P.

## References

[1] Taberlet P, Coissac E, Pompanon F, et al. Towards next-generation biodiversity assessment using DNA metabarcoding. Mol Ecol. 2012;21(8):2045–50. 22486824 10.1111/j.1365-294X.2012.05470.x

[2] Clare EL . Molecular detection of trophic interactions: emerging trends, distinct advantages, significant considerations and conservation applications. Evol Appl. 2014;7(9):1144–57. 25553074 10.1111/eva.12225 PMC4231602

[3] Paula DP . Next-generation sequencing and its impacts on entomological research in ecology and evolution. Neotrop Entomol. 2021;50(5):679–96. 34374956 10.1007/s13744-021-00895-x

[4] Schloss PD, Westcott SL, Ryabin T, et al. Introducing mothur: open-source, platform-independent, community-supported software for describing and comparing microbial communities. Appl Environ Microbiol. 2009;75(23):7537–41. 19801464 10.1128/AEM.01541-09 PMC2786419

[5] Caporaso JG, Kuczynski J, Stombaugh J, et al. QIIME allows analysis of high-throughput community sequencing data. Nat Methods. 2010;7(5):335–6. 20383131 10.1038/nmeth.f.303 PMC3156573

[6] Boyer F, Mercier C, Bonin A, et al. Obitools: a unix-inspired software package for DNA metabarcoding. Mol Ecol Resour. 2016;16(1):176–82. 25959493 10.1111/1755-0998.12428

[7] Callahan BJ, McMurdie PJ, Rosen MJ, et al. DADA2: high-resolution sample inference from Illumina amplicon data. Nat Methods. 2016;13(7):581–3. 27214047 10.1038/nmeth.3869 PMC4927377

[8] Anslan S, Bahram M, Hiiesalu I, et al. PipeCraft: flexible open-source toolkit for bioinformatics analysis of custom high-throughput amplicon sequencing data. Mol Ecol Resour. 2017;17(6):e234. 28544559 10.1111/1755-0998.12692

[9] Taberlet P, Bonin A, Zinger L, et al. Environmental DNA: for biodiversity research and monitoring. 1st ed. Oxford University Press; 2018.

[10] Deagle BE, Jarman SN, Coissac E, et al. DNA metabarcoding and the cytochrome c oxidase subunit I marker: not a perfect match. Biol Lett. 2014;10(9):doi:10.1098/rsbl.2014.0562. PMC4190964 25209199

[11] Clarke LJ, Soubrier J, Weyrich LS, et al. Environmental metabarcodes for insects: *in silico* PCR reveals potential for taxonomic bias. Mol Ecol Resour. 2014;14(6):1160–70. 24751203 10.1111/1755-0998.12265

[12] Elbrecht V, Leese F. Can DNA-based ecosystem assessments quantify species abundance? Testing primer bias and biomass-sequence relationships with an innovative metabarcoding protocol. PLoS One. 2015;10(7):e0130324. 26154168 10.1371/journal.pone.0130324 PMC4496048

[13] Elbrecht V, Leese F. Validation and development of COI metabarcoding primers for freshwater macroinvertebrate bioassessment. Front Environ Sci. 2017;5:11.

[14] Haas BJ, Gevers D, Earl AM, et al. Chimeric 16S rRNA sequence formation and detection in Sanger and 454-pyrosequencing PCR amplicons. Genome Res. 2011;21(3):494. 21212162 10.1101/gr.112730.110 PMC3044863

[15] Zhou X, Li Y, Liu S, et al. Ultra-deep sequencing enables high-fidelity recovery of biodiversity for bulk arthropod samples without PCR amplification. Gigascience. 2013;2(1):4. 23587339 10.1186/2047-217X-2-4 PMC3637469

[16] Gillett CP, Crampton-Platt A, Timmermans MJTN, et al. Bulk *de novo* mitogenome assembly from pooled total DNA elucidates the phylogeny of weevils (Coleoptera: Curculionoidea). Mol Biol Evol. 2014;31(8):2223–37. 24803639 10.1093/molbev/msu154 PMC4104315

[17] Tang M, Tan M, Meng G, et al. Multiple, sequencing of pooled mitochondrial genomes - a crucial step toward biodiversity analysis using mito-metagenomics. Nucleic Acids Res. 2014;42(22):e166. 10.1093/nar/gku917 PMC4267667 25294837

[18] Andujar C, Arribas P, Ruzicka F, et al. Phylogenetic community ecology of soil biodiversity using mitochondrial metagenomics. Mol Ecol. 2015;24(14):3603–17. 25865150 10.1111/mec.13195

[19] Crampton-Platt AL, Timmermans MJTN, Gimmel ML, et al. Soup to tree: the phylogeny of beetles inferred by mitochondrial metagenomics of a Bornean rainforest sample. Mol Biol Evol. 2015;32(9):2302–16. 25957318 10.1093/molbev/msv111 PMC4540967

[20] Gomez-Rodrıguez C, Crampton-Platt A, Timmermans MJ, et al. Validating the power of mitochondrial metagenomics for community ecology and phylogenetics of complex assemblages. Methods Ecol Evol. 2015;6(8):883–94.

[21] Liu S, Wang X, Xie L, et al. Mitochondrial capture enriches mito-DNA 100 fold, enabling PCR-free mitogenomics biodiversity analysis. Mol Ecol Resour. 2016;16(2):470–9. 26425990 10.1111/1755-0998.12472

[22] Linard B, Crampton-Platt A, Timmermans MJTN, et al. Metagenome skimming of insect specimen pools: potential for comparative genomics. Genome Biol Evol. 2015;7(6):1474–89. 25979752 10.1093/gbe/evv086 PMC4494052

[23] Shokralla S, Gibson J, King I, et al. Environmental DNA barcode sequence capture: targeted, PCR-free sequence capture for biodiversity analysis from bulk environmental samples. bioRxiv 2016:doi:10.1101/087437.

[24] Sarmashghi S, Bohmann K, Gilbert MTP, et al. Skmer: assembly-free and alignment-free sample identification using genome skims. Genome Biol. 2019;20(1):34. 30760303 10.1186/s13059-019-1632-4 PMC6374904

[25] Ji Y, Huotari T, Roslin T, et al. SPIKEPIPE: a metagenomic pipeline for the accurate quantification of eukaryotic species occurrences and intraspecific abundance change using DNA barcodes or mitogenomes. Mol Ecol Resour. 2020;20(1):256–67. 31293086 10.1111/1755-0998.13057

[26] Srivathsan A, Sha JCM, Vogler AP, et al. Comparing the effectiveness of metagenomics and metabarcoding for diet analysis of a leaf feeding monkey (*Pygathrix nemaeus*). Mol Ecol Resour. 2015;15(2):250–61. 25042073 10.1111/1755-0998.12302

[27] Srivathsan A, Ang A, Vogler AP, et al. Fecal metagenomics for the simultaneous assessment of diet, parasites, and population genetics of an understudied primate. Front Zool. 2016;13(1):17. 27103937 10.1186/s12983-016-0150-4 PMC4839110

[28] Paula DP, Linard B, Andow DA, et al. Detection and decay rates of prey and prey symbionts in the gut of a predator through metagenomics. Mol Ecol Resour. 2015;15(4):880–92. 25545417 10.1111/1755-0998.12364

[29] Paula DP, Linard B, Platt AC, et al. Uncovering trophic interactions in arthropod predators through DNA shotgun-sequencing of gut contents. PLoS One. 2016; 11(9):e0161841. 27622637 10.1371/journal.pone.0161841 PMC5021305

[30] Aquino AM, Aguiar-Menezes EL, Queiroz JM. Recomendações para coleta de artrópodes terrestres por armadilhas de queda (“pitfall-traps”). Circular Técnica. 16. Rio de Janeiro: Empresa Brasileira de Pesquisa Agropecuária; 2006.

[31] Sutherland WJ . Ecological Census Techniques: a handbook. 2nd ed. Cambridge: Cambridge University Press; 1996.

[32] Greenstone MH, Weber DC, Coudron TA, et al. Removing external DNA contamination from arthropod predators destined for molecular gut-content analysis. Mol Ecol Resour. 2012;12(3):464–9. 22268594 10.1111/j.1755-0998.2012.03112.x

[33] Zaidi RH, Jaal Z, Hawkes NJ, et al. Can multiple-copy sequences of prey DNA be detected amongst the gut contents of invertebrate predators?. Mol Ecol. 1999;8(12):2081–7. 10632859 10.1046/j.1365-294x.1999.00823.x

[34] Elbrecht V, Taberlet P, Dejean T, et al. Testing the potential of a ribosomal 16S marker for DNA metabarcoding of insects. PeerJ. 2016;4:e1966. 27114891 10.7717/peerj.1966 PMC4841222

[35] Sousa LL, Silva SM, Xavier R. DNA metabarcoding in diet studies: unveiling ecological aspects in aquatic and terrestrial ecosystems. Environ DNA. 2019;1(3):199–214.

[36] O'Donnell JL, Kelly RP, Lowell NC, et al. Indexed PCR primers induce template-specific bias in large-scale DNA sequencing studies. PLoS One. 2016;11(3):e0148698. 26950069 10.1371/journal.pone.0148698 PMC4780811

[37] Juen A, Traugott M. Amplification facilitators and multiplex PCR: tools to overcome PCR-inhibition in DNA-gut-content analysis of soil-living invertebrates. Soil Biol Biochem. 2006;38(7):1872–9.

[38] Ficetola GF, Coissac E, Zundel S, et al. An *in silico* approach for the evaluation of DNA barcodes. BMC Genomics. 2010;11(1):434. 20637073 10.1186/1471-2164-11-434 PMC3091633

[39] Stoesser G, Moseley MA, Sleep J, et al. The EMBL Nucleotide Sequence Database. Nucleic Acids Res. 1998;26(1):8–15. 9399791 10.1093/nar/26.1.8 PMC147241

[40] De Barba M, Miquel C, Boyer F, et al. DNA metabarcoding multiplexing and validation of data accuracy for diet assessment: application to omnivorous diet. Mol Ecol Resour. 2014;14(2):306–23. 24128180 10.1111/1755-0998.12188

[41] Quéméré E, Hibert F, Miquel C, et al. A DNA metabarcoding study of a primate dietary diversity and plasticity across its entire fragmented range. PLoS One. 2013;8(3):e58971. 23527060 10.1371/journal.pone.0058971 PMC3602585

[42] Paula DP, Timbó RV, Togawa RC, et al. Quantitative prey species detection in predator guts across multiple trophic levels by DNA shotgun sequencing. bioRxiv 2021:doi:10.1101/2021.04.01.438119. 35852519

[43] Paula DP, Barros SKA, Pitta RM, et al. Supporting data for “Metabarcoding versus mapping unassembled shotgun reads for identification of prey consumed by arthropod epigeal predators.”. GigaScience Database. 2022. 10.5524/100970. PMC8952265 35333301

[44] Andrews D . FastQC: a quality control tool for high throughput sequence data. 2010. http://www.bioinformatics.babraham.ac.uk/projects/fastqc.

[45] Aronesty E . ea-utils: command-line tools for processing biological sequencing data. 2011. https://github.com/ExpressionAnalysis/ea-utils/.

[46] Martin M . Cutadapt removes adapter sequences from high-throughput sequencing reads. EMBnet J. 2011;17(1):10–2.

[47] Shen W, Le S, Li Y, et al. SeqKit: a cross-platform and ultrafast toolkit for FASTA/Q file manipulation. PLoS One. 2016;11(10):e0163962. 27706213 10.1371/journal.pone.0163962 PMC5051824

[48] Ririe KM, Rasmussen RP, Wittwer CT. Product differentiation by analysis of DNA melting curves during the polymerase chain reaction. Anal Biochem. 1997;245(2):154–60. 9056205 10.1006/abio.1996.9916

[49] Zhang T, Fang HH. 16S rDNA clone library screening of environmental sample using melting curve analysis. J Chin Inst Eng. 2005;28(7):1153–5.

[50] Winder L, Phillips C, Richards N, et al. Evaluation of DNA melting analysis as a tool for species identification. Methods Ecol Evol. 2011; 2(3):312–20.

[51] Perera OP, Allen KC, Jain D, et al. Rapid identification of *Helicoverpa armigera* and *Helicoverpa zea* (Lepidoptera: Noctuidae) using ribosomal RNA internal transcribed spacer 1. J Insect Sci. 2015;15(1):155. 26516166 10.1093/jisesa/iev137 PMC4625950

[52] Paula DP, Andow DA. Melting curve analysis for detection and identification of ghost parasitoids in host carcasses a month after host death. Methods Ecol Evol. 2021;12(9):1552–61.

[53] Kearse M, Moir R, Wilson A, et al. Geneious Basic: an integrated and extendable desktop software platform for the organization and analysis of sequence data. Bioinformatics. 2012;28(12):1647–9. 22543367 10.1093/bioinformatics/bts199 PMC3371832

[54] Ye J, Coulouris G, Zaretskaya I, et al. Primer-BLAST: a tool to design target-specific primers for polymerase chain reaction. BMC Bioinformatics. 2012;13(1):134. 22708584 10.1186/1471-2105-13-134 PMC3412702

[55] R Core Team . R: A language and environment for statistical computing. Vienna, Austria: R Foundation for Statisical Computing; 2019.

[56] Altman DG, Bland JM. Diagnostic tests. 1: Sensitivity and specificity. BMJ. 1994;308(6943):1552. 8019315 10.1136/bmj.308.6943.1552 PMC2540489

[57] Fletcher RH, Fletcher SW, Fletcher GS. Clinical Epidemiology: the essentials. 4th ed. Lippincott Williams & Wilkins; 2005.

[58] Ruijter JM, Ramakers C, Hoogaars WMH, et al. Amplification efficiency: linking baseline and bias in the analysis of quantitative PCR data. Nucleic Acids Res. 2009;37(6):e45. 19237396 10.1093/nar/gkp045 PMC2665230

[59] Richardson RT, Bengtsson-Palme J, Johnson RM. Evaluating and optimizing the performance of software commonly used for the taxonomic classification of DNA metabarcoding sequence data. Mol Ecol Resour. 2017;17(4):760–9. 27797448 10.1111/1755-0998.12628

[60] Meusnier I, Singer GA, Landry JF, et al. A universal DNA mini-barcode for biodiversity analysis. BMC Genomics. 2008;9(1):214. 18474098 10.1186/1471-2164-9-214 PMC2396642

[61] Leray M, Yang JY, Meyer CP, et al. A new versatile primer set targeting a short fragment of the mitochondrial COI region for metabarcoding metazoan diversity: application for characterizing coral reef fish gut contents. Front Zool. 2013;10(1):34. 23767809 10.1186/1742-9994-10-34 PMC3686579

[62] Gibson J, Shokralla S, Porter TM, et al. Simultaneous assessment of the macrobiome and microbiome in a bulk sample of tropical arthropods through DNA metasystematics. Proc Natl Acad Sci U S A. 2014;111(22):8007–12. 24808136 10.1073/pnas.1406468111 PMC4050544

[63] Nichols RV, Vollmers C, Newsom LA, et al. Minimizing polymerase biases in metabarcoding. Mol Ecol Resour. 2018;18(5):927–39. 10.1111/1755-0998.12895 29797549

[64] Nilsson RH, Tedersoo L, Lindahl BD, et al. Towards standardization of the description and publication of next-generation sequencing datasets of fungal communities. New Phytol. 2011;191(2):314–8. 21557749 10.1111/j.1469-8137.2011.03755.x

[65] Tedersoo L, Ramirez KS, Nilsson RH, et al. Standardizing metadata and taxonomic identification in metabarcoding studies. Gigascience. 2015;4:34. 26236474 10.1186/s13742-015-0074-5 PMC4521374

[66] Piñol J, San Andrés V, Clare EL, et al. A pragmatic approach to the analysis of diets of generalist predators: the use of next-generation sequencing with no blocking probes. Mol Ecol Resour. 2014;14(1):18–26. 23957910 10.1111/1755-0998.12156

[67] Robasky K, Lewis NE, Church GM. The role of replicates for error mitigation in next-generation sequencing. Nat Rev Genet. 2014;15(1):56–62. 24322726 10.1038/nrg3655 PMC4103745

[68] Munch K, Boomsma W, Huelsenbeck JP, et al. Statistical assignment of DNA sequences using Bayesian phylogenetics. Syst Biol. 2008;57(5):750–7. 18853361 10.1080/10635150802422316

[69] Alberdi A, Aizpurua O, Gilbert MT, et al. Scrutinizing key steps for reliable metabarcoding of environmental samples. Methods Ecol Evol. 2018;9(1):134–47.

[70] Folmer O, Black M, Hoeh W, et al. DNA primers for amplification of mitochondrial cytochrome c oxidase subunit I from diverse metazoan invertebrates. Mol Mar Biol Biotechnol. 1994;3(5):294–9. 7881515

[71] Deagle BE, Thomas AC, McInnes JC, et al. Counting with DNA in metabarcoding studies: how should we convert sequence reads to dietary data?. Mol Ecol. 2019;28(2):391–406. 29858539 10.1111/mec.14734 PMC6905394

[72] Piñol J, Mir G, Gomez-Polo P, et al. Universal and blocking primer mismatches limit the use of high throughput DNA sequencing for the quantitative metabarcoding of arthropods. Mol Ecol Resour. 2015;15(4):819–30. 25454249 10.1111/1755-0998.12355

[73] Piñol J, Senar MA, Symondson WO. The choice of universal primers and the characteristics of the species mixture determines when DNA metabarcoding can be quantitative. Mol Ecol. 2019;28(2):407–19. 29939447 10.1111/mec.14776

[74] Thomas AC, Deagle BE, Eveson JP, et al. Quantitative DNA metabarcoding: improved estimates of species proportional biomass using correction factors derived from control material. Mol Ecol Resour. 2016;16(3):714–26. 26602877 10.1111/1755-0998.12490

[75] Bista I, Carvalho GR, Tang M, et al. Performance of amplicon and shotgun sequencing for accurate biomass estimation in invertebrate community samples. Mol Ecol Resour. 2018;18(5):1020–34. 10.1111/1755-0998.12888 29667329

[76] Lamb PD, Hunter E, Pinnegar JK, et al. How quantitative is metabarcoding: a meta-analytical approach. Mol Ecol. 2019;28(2):420–30. 30408260 10.1111/mec.14920 PMC7379500

