# Supplementary material for: Metabarcoding versus mapping unassembled shotgun reads for identification of prey consumed by arthropod epigeal predators
Source: Gigascience. 2022 Mar 24;11:giac020. doi: 10.1093/gigascience/giac020 (PMC8952265; doi:10.1093/gigascience/giac020)
Supplement: giac020_GIGA-D-21-00303_Original_Submission [file giac020_giga-d-21-00303_original_submission.pdf]

GigaScience

Metabarcoding versus mapping unassembled shotgun reads for identification of prey consumed by arthropod epigeal predators

--Manuscript Draft--

**Manuscript Number:**	GIGA-D-21-00303
**Full Title:**	Metabarcoding versus mapping unassembled shotgun reads for identification of prey consumed by arthropod epigeal predators
**Article Type:**	Research
**Funding Information:**	Agricultural Research Service (USDA-NIFA 2016-67030-24950) — PhD David Andow
**Abstract:**	Background: A central challenge of DNA gut content analysis is to identify prey in a highly degraded DNA community. In this study, we evaluated prey detection using metabarcoding and a method of mapping unassembled shotgun reads (Lazaro). Results: In a control mock prey community, metabarcoding did not detect any prey, while Lazaro detected prey with accuracy 43-71%. Gut content analysis of field-collected arthropod epigeal predators (three ants, one dermapteran and one carabidae) from agricultural habitats in Brazil (27 samples, 46-237 individuals per sample) revealed that 64% of the prey species detections by either method were not confirmed by Melting Curve Analysis and 87% of the true prey were detected in common. We hypothesized that Lazaro would detect fewer true and false positive and more false negative prey with greater taxonomic resolution than metabarcoding, but found that the methods were similar in these respects and had similar accuracy (metabarcoding: 64%; Lazaro: 67%). While metabarcoding detected 350× more true prey reads than Lazaro, this did not convert into a higher number of true positive prey detections. There was a positive correlation between the relative prey DNA concentration in the samples and the number of prey reads detected by Lazaro, while this was inconsistent for metabarcoding. Conclusions: Metabarcoding and Lazaro had similar, but partially complementary, detection of prey in arthropod predator guts. However, while Lazaro was almost 2× more expensive, read number was related to the amount of prey DNA, suggesting that Lazaro can provide quantitative prey information while metabarcoding may not.
**Corresponding Author:**	debora Pires paula, PhD<br>Embrapa: Empresa Brasileira de Pesquisa Agropecuaria<br>Brasilia, DF BRAZIL
**Corresponding Author Secondary Information:**	
**Corresponding Author's Institution:**	Embrapa: Empresa Brasileira de Pesquisa Agropecuaria
**Corresponding Author's Secondary Institution:**	
**First Author:**	debora Pires paula, PhD
**First Author Secondary Information:**	
**Order of Authors:**	debora Pires paula, PhD
	Suellen Barros
	Rafael Pitta
	Marlinto Barreto
	Roberto Togawa
	David Andow
**Order of Authors Secondary Information:**	
**Additional Information:**	
**Question**	**Response**

Powered by Editorial Manager® and ProduXion Manager® from Aries Systems Corporation

Are you submitting this manuscript to a special series or article collection?	No
**Experimental design and statistics**<br><br>Full details of the experimental design and statistical methods used should be given in the Methods section, as detailed in our Minimum Standards Reporting Checklist. Information essential to interpreting the data presented should be made available in the figure legends.<br><br>Have you included all the information requested in your manuscript?	Yes
**Resources**<br><br>A description of all resources used, including antibodies, cell lines, animals and software tools, with enough information to allow them to be uniquely identified, should be included in the Methods section. Authors are strongly encouraged to cite Research Resource Identifiers (RRIDs) for antibodies, model organisms and tools, where possible.<br><br>Have you included the information requested as detailed in our Minimum Standards Reporting Checklist?	Yes
**Availability of data and materials**<br><br>All datasets and code on which the conclusions of the paper rely must be either included in your submission or deposited in publicly available repositories (where available and ethically appropriate), referencing such data using a unique identifier in the references and in the "Availability of Data and Materials" section of your manuscript.<br><br>Have you have met the above requirement as detailed in our Minimum	Yes

Standards Reporting Checklist?	

Powered by Editorial Manager® and ProduXion Manager® from Aries Systems Corporation

Manuscript
Click here to access/download;Manuscript;Main text version R1 Sep 24 2021.docx

Metabarcoding versus mapping unassembled shotgun reads for identification of prey consumed by arthropod epigeal predators

Débora Pires Paula[1,*], Suellen Karina Albertoni Barros[2], Rafael Major Pitta[3], Marliton Rocha Barreto[2], Roberto Coiti Togawa[1], David A. Andow[4]

[1] Embrapa Genetic Resources and Biotechnology, Brasília-DF, Brazil;

[2] Universidade Federal de Mato Grosso, Sinop-MT, Brasil;

[3] Embrapa Agrosilvopastoral, Sinop-MT, Brazil

[4] Department of Entomology, University of Minnesota, MN, USA.

*Corresponding author: debora.pires@embrapa.br

Phone: +55 (61) 34484929; Fax: +55 (61) 34484672

Abstract

Background: A central challenge of DNA gut content analysis is to identify prey in a highly degraded DNA community. In this study, we evaluated prey detection using metabarcoding and a method of mapping unassembled shotgun reads (Lazaro). **Results:** In a control mock prey community, metabarcoding did not detect any prey, while Lazaro detected prey with accuracy 43-71%. Gut content analysis of field-collected arthropod epigeal predators (three ants, one dermapteran and one carabidae) from agricultural habitats in Brazil (27 samples, 46-237 individuals per sample) revealed that 64% of the prey species detections by either method were not confirmed by Melting Curve Analysis and 87% of the true prey were detected in common. We hypothesized that Lazaro would detect fewer true and false positive and more false negative prey with greater taxonomic resolution than metabarcoding, but found that the methods were similar in these respects and had similar accuracy (metabarcoding: 64%; Lazaro: 67%). While metabarcoding detected 350× more true prey reads than Lazaro, this did not convert into a higher number of true positive prey detections. There was a positive correlation between the relative prey DNA concentration in the samples and the number of prey reads detected by Lazaro, while this was inconsistent for metabarcoding. **Conclusions:** Metabarcoding and Lazaro had similar, but partially complementary, detection of prey in arthropod predator guts. However, while Lazaro was almost 2× more expensive, read number was related to the amount of prey DNA, suggesting that Lazaro can provide quantitative prey information while metabarcoding may not.

Keywords: diet analysis; environmental DNA; generalist predators; gut content analysis.

Data Description

Background

The use of high throughput DNA sequencing (HTS) for studying species composition or diversity in environmental samples has been widely adopted and metabarcoding has become the most commonly used method to study environmental DNA (eDNA) [1-3]. In metabarcoding, target barcode regions are enriched through PCR and sequenced for taxonomic identification (specific taxa or operational taxonomic units; OTUs) through a bioinformatic workflow [4-8] by similarity of query sequences with taxonomically identified sequences in a reference database [9].

The main limitations of metabarcoding are related to bias in primer amplification efficiency during the target barcode enrichment process and amplification errors [10-13]. Optimal primer pair(s) would amplify the barcode region(s) of a broad taxonomic range with equivalent efficiency across taxa, avoid formation of chimeras among closely related or abundant sequences, and provide the desired taxonomic resolution without missing any taxon [9,10,12,14]. Such a primer pair has yet to be found, so most recent metabarcoding studies design primers have focused on amplifying barcodes of specific taxonomic groups and/or employing multiple primer pairs for the same or different barcodes. In addition, for predator gut content analysis, the more common predator barcode sequences could mask amplification of closely related species.

Alternative methods for species identification that have no sample DNA enrichment have been developed, and include those that assemble or not assemble the reads previously to mapping them to a reference database. Methods with no sample DNA enrichment in which reads are assembled include mitochondrial metagenomics [15-21], metagenome skimming [22], and enrichment of a barcode sequence by hybridization capture followed by HTS [23]. Mitochondrial metagenomics and metagenome skimming are promising methods for general biodiversity surveys,

but not for predator gut content analysis because they rely on assembling genomes (organelles or nuclear genetic material) to function as a 'superbarcode' to identify species. As the DNA community in predator guts is degraded by digestion, satisfactory assembly of prey mitochondria with sufficient coverage is difficult and, therefore, the application of mitochondrial metagenomics and metagenome skimming is compromised. Hybridization capture replaces PCR to enrich the barcode sequences in a sample, and is suitable for gut content analysis. However, as it still depends on an intermediate step of enrichment of a particular barcode, it might also be subject to bias related to probe design and fidelity/efficiency of the hybridization.

Assembly-free methods have been more recently proposed [24;25], but just a few were tested for gut content analysis [26-29]. Their principle basically comprises the direct eDNA sequencing and mapping the unassembled reads to a reference database for taxa identification using a threshold of high similarity (>95%) with a minimum predefined overlap length for the matches. No barcode primer pair or probe is required, hypothetically minimizing bias and favoring quantitative estimates of prey content. Without amplification, however, the detection of rare eDNA is likely reduced. A major limitation is that different samples cannot be multiplexed in a single or few libraries because there is no sample DNA enrichment step where individual tags are assigned to each sample. Consequently, every sample has to have its own library, increasing the total cost in library construction. In addition, while all methods based on DNA similarity require a reference database, Lazaro requires mitogenomes, other organellar genomes or nuclear genome fragments in the reference database [29]. Therefore, suitable sequences of candidate prey (*e.g.*, species co-occurring with the predators) are often missing from the database, so they need to be elucidated and added to the reference database, otherwise the prey cannot be detected.

Despite great advances and several options, at the moment, there is no unanimous consensus on a "best practice" method for eDNA study of gut contents [9]. To improve the

applicability of large-scale DNA-based methods for prey detection, this work examined the sensitivity, specificity and accuracy of using metabarcoding and a mapping of unassembled shotgun read method (Lazaro) to identify prey in the guts of several epigeal agricultural predators. We used Melt Curve Analysis (MCA) to verify detections by the two methods and examined the number of true and false prey species detected, the number of true and false non-detections recorded, the taxonomic resolution of true detections, and the relation between the number of reads for a detection and the relative prey DNA concentration.

Materials and Methods

Control mock prey community

Newly emerged (48 h) unfed harlequin ladybird *Harmonia axyridis* (Coleoptera: Coccinellidae) adults (n=10, sex ratio of 1:1) were individually supplied simultaneously with seven species of prey, which were consumed within one hour. These were one adult apterae of the aphids (Hemiptera: Aphididae) *Aphis glycines*, *A. gossypii*, *A. craccivora*, *Acyrthosiphon pisum* and *Myzus persicae*, and one egg of the diamondback moth *Plutella xylostella* (Lepidoptera: Plutellidae) and *Cycloneda munda* (Coleoptera: Coccinellidae). Immediately before feeding (negative control) and after feeding, five beetles per sex were placed in 95% ethanol and stored at -80°C.

Arthropod field sampling

Epigeal arthropod predators were sampled twice a month (Brazilian authorization SISBIO 33683-1) in 2014/2015 (July-September) in Sinop-MT/Brazil for a 24 h period [30] in pitfall-traps buried level with the soil in four replicated agricultural experimental plots: soybean/maize (*Glycines max*/*Zea mays*), palisade grass (*Brachiaria brizantha*), eucalyptus plantation (hybrid of *Eucalyptus*

grandis and *E. urophylla*), and an additive mixture of all three. Pitfall-traps contained 750 ml of water and two drops of detergent to break surface tension and preserve the captured specimens [31]. We obtained 12 samples for each of the two more abundant ant species (Hymenoptera: Formicidae), *Pheidole flavens* (n=200 specimens/sample) and *Dorymyrmex brunneus* (n=100 specimens/sample), and one sample of *Solenopsis substituta* (n=273), one sample of the earwig *Euborellia annulipes* (Dermaptera: Anisolabididae) (n=46) and one sample of the tiger beetle *Tetracha* sp. (Coleoptera: Carabidae) (n=49). These species were the most abundant predator species sampled and all the specimens were used for DNA extraction.

DNA extractions

To clean external DNA from the specimens, all the specimens from the feeding bioassay controls and from the field, before DNA extraction, were soaked individually for 40 min in 2.5% commercial bleach in 1.5 microtubes, followed by orbital rotation at 2×*g* at 4°C for 40 min, discarding the washing solution and rinsing the specimens for 5× in ultrapure water [32]. For the ants, the gaster was separated and collected, and for the other species, guts were dissected under a microscope (30× magnification) immediately before DNA extraction using sterilized entomological dissecting tools. Sterilization was performed by soaking the dissection tools in 0.5% sodium hypochlorite for 10 min and autoclavating (121°C at 1 atm for 20 min), followed by rising abundantly with ultrapure water (MilliQ) to minimize cross-contamination. Dissected guts or gasters from the same sample were pooled in a lysis buffer from the kit DNeasy Blood & Tissue (Qiagen), placed on ice and macerated with sterilized glass pestles separately for each sample. Cross-contamination was minimized by sanitizing surfaces and sterilizing all equipment and materials between specimen dissections, and filter tips were used to handle all liquids containing DNA. Total DNA extraction was performed using DNeasy Blood & Tissue (Qiagen) kit. DNA

purity and concentration were assessed by the NanoDrop^{TM} spectrophotometer. DNA quantity was normalized to 1 mg/ml across samples and split into three parts, one for Lazaro, one for metabarcoding, and one for MCA in qPCR.

Preparation of the DNA samples for metabarcoding and Lazaro analyses

For Lazaro, the pertinent aliquots obtained from the previous step were normalized to 150 ng DNA/sample. For metabarcoding, a region of the 16S mitochondrial gene was amplified using the primer pair Ins16S_1short (forward 5´-TRRGACGAGAAGACCCTATA-3´ and reverse 5´-ACGCTGTTATCCCTAAGGTA-3´), which generates an amplicon of around 190 bp [11]. 16S barcode was chosen over COI for several reasons. Although its arthropod database had considerably fewer sequences (133,899 sequences, 2,630 families, 5,829 genera and 48,711 species, obtained from GenBank using the search *arthropod[organism] AND 16S*, release date to 2018/12/31) than the COI invertebrate database (2,570,787 sequences from 1,395 families, 10,394 genera and 26,024 species; obtained from GenBank using the search *coi[Gene Name] AND arthropoda[Organism] AND "1900"[Publication Date] : "2018/12/31"[Publication Date]*), it had higher taxonomic coverage. In addition, Clarke et al. [11] demonstrated better taxonomic coverage of 16S than COI and bias of COI to amplify more lepidopterans and dipterans while failing to amplify other insect orders (e.g., hymenopterans). Lastly, according to Deagle et al. [10], Elbrecht et al. [33] and Sousa et al. [34], 16S has been preferentially used because 16S has some regions of more conserved sites across taxonomic groups, spanning sufficiently variable regions among taxa, resulting in more universal primers with equivalent taxonomic resolution than COI. Primers were not tagged to eliminate bias related to the tagging process [35], so an independent library was produced for each epigeal predator DNA gut sample. PCR reactions (0.2 µM primer pair) were performed in triplicate using Qiagen Multiplex PCR Master Mix and adding 1.28 µg/µl of bovine

serum albumin (BSA) to prevent PCR inhibition [36]. Cycling conditions were: initial heat activation 15 min at 95°C, 40 cycles of 3-step cycling (denaturation 30 s at 94°C, annealing 90 s at 60°C, extension 90 s 72°C), and final extension for 10 min at 72°C. Triplicates were pooled and purified using QIAquick PCR Purification Kit (Qiagen). Amplicons were quantified by NanoDrop™ and normalized in equimolar ratios across all the metabarcoding samples. We opted for not multiplexing the 27 metabarcoding samples to keep the same coverage between metabarcoding and Lazaro methods.

DNA sequencing

All Lazaro and enriched barcode samples were dried in a speed vacuum centrifuge. For the feeding bioassay samples, 20 samples were dried, which comprised 5 treatments (without feeding and four times after feeding)×2 sexes×2 methods (metabarcoding and Lazaro). For the field samples, 54 samples were dried, which comprised 27 for metabarcoding and 27 for Lazaro. The dried feeding bioassay and field samples were shipped simultaneously to the Roy J. Carver Biotechnology Center (University of Illinois at Urbana-Champaign) to construct KAPA Hyper libraries (Kapa Biosystems) with insert size 200 to 600 bp using unique dual indexes. Quality checked samples were sequenced by Illumina HiSeq4000 (150 bp paired-end, 151 cycles, HiSeq 4000 sequencing kit version 1) in a single lane. The Brazilian license to access the genetic heritage was provided by CGEN/SISGEN A8E3D94. Sequence Read Archive (SRA) access codes are presented in Supporting Information 1.

Reference DNA databases and bioinformatic analysis

For metabarcoding, the reference database was constructed by extracting invertebrate 16S barcode regions from the European Nucleotide Sequence database (EMBL) (release 132,

ftp://ftp.ebi.ac.uk/pub/databases/embl/release/; inv: invertebrate database/division; std: standard) using the ecoPCR program [37]. The EMBL is shared daily with GenBank (from USA) and DDBJ (from Japan) databases [38]. In addition, 16S sequences for several species that were collected in the pitfall traps were determined and added to the reference database. Prey detection analysis was performed using OBITools (http://metabarcoding.org/obitools) as in [26, 39,40]. The metabarcoding threshold for taxonomic assignment was 98% identity and reads with count lower than 100 were removed. Only 'head' and 'singleton' identifications were considered.

For the Lazaro reference database [28,29], we constructed a comprehensive arthropod mitochondrial DNA database by obtaining all sequences (partial or complete, Fasta format) available at the time at GenBank (n=3,381, distributed in 2,779 species from 1,850 genera in 598 families). In addition, following the mitochondrial elucidation method described in Paula et al. [28,29], we provided mitochondrial sequences of 29 taxa (Table S1) corresponding to the main potential prey co-occurring with the sampled epigeal predators in the experimental plots, including the predators under analysis (taxa and taxonomic determinations in Table S2). For the taxonomic prey identification, we used the Lazaro method [41], which is designed to detect and quantify species from degraded eDNA samples. Briefly, this method takes raw BlastN output of hit matches, identifies the mismatches (or SNPs) between the query and reference sequence, removes false mismatches (e.g., degenerate IUPAC nucleotide codes, e.g., R=A or G; Y=T or C; S=C or G; etc), reanalyzes overlap length and percent identity, filters the best hit matches with an overlap-identity threshold, eliminates singleton reads, and filters the reads mapping to coding regions of their respective reference mitogenome. The scripts are available in the GitHub repository: https://github.com/molecular-ecology/DDSS. The best overlap-identity threshold was determined from previous experimental data [41] and determined to be at least 100% identity in an overlap length of at least 130 bp (Supporting Information 1). Fastq files were generated and

demultiplexed with the bcl2fastq v2.17.1.14 Conversion Software (Illumina). The quality assessment for each dataset was done using FastQC (v.0.11.3) [42]. Low quality sequences (Phred<30) and library index adaptors were trimmed by Fastqc-mcf (v.1.04.807) [43] and Cutadapt (v.1.9.1) [44]. Retained good quality Fastq reads were converted to Fasta format by SeqTK (v1.2) [45].

MCA confirmation of the field detected prey

For the field samples, we performed Melting Curve Analysis (MCA) in qPCR to check the presence of the prey DNA detected by metabarcoding and Lazaro. The principle is based on the estimation of the melting temperature (Tm), is the temperature at which 50% of the two strands of DNA dissociate, a property dependent on nucleotide composition and product length [46,47]. By monitoring denaturation of the PCR products with SYBR Green and fluorescence levels over a temperature gradient, it is possible to construct the melting curve [48,49]. Confirmation of true positive detections occurred when the Tm of the sample was within 1°C of the true positive control for at least two replicates, and the peak was sharp enough that the absolute value of the slope of the melt curve was greater than 0.6. The DNA source was the original DNA extracted from the gut contents of the predators. For 28 of the 32 species potentially detected as prey, we obtained specimens with confirmed taxonomy to determine a positive control reference melting temperature (Tm) to distinguish true and false positive detections. Their DNA was extracted using the DNeasy Blood & Tissue (Qiagen) kit. Species-specific primer pairs were designed as in Paula and Andow [50], nearly all in regions of the mitogenome (Table S3), for all prey species detected by metabarcoding and Lazaro, using the program Primer 3 at Geneious v7.1.9 [51] and checked in NCBI/Primer-BLAST [52]. The cross-reactivity of these primers with related detected species is presented in Supporting Information 1 (Fig. S1 to S4). The qPCR reactions (13 µl) were prepared

using Thermo Scientific Maxima SYBR Green/ROX qPCR Master Mix (2×), 1.28 µg/µl of BSA and 10 ng of DNA per reaction and each specific primer pair at 0.3 µM. The amplifications were performed in 384-well plates with a Roche Applied Science LightCycler® 480 Real-Time PCR System using a two-step cycling protocol (initial denaturation at 95°C for 10 min, ramp 4.4°C/s), and 40 cycles of denaturation at 95°C for 15 s (ramp 4.4°C/s) and annealing/extension at 60°C for 60 s (ramp 2.2°C/s), and a melt curve from 60°C to 95°C continuous (ramp 1°C/s) with six readings/°C. qPCR for each sample was performed in at least three technical replicates. No-template controls (NTC) were included for every primer pair. Melt curves were constructed using the raw fluorescence data and diffQ in the library MBmca in *R* [53]. Positive prey detection and identification were considered if at least two technical replicates had -dF/dT more than 0.1 above background or if one technical replicate had -dF/dT more than 0.2 above background at the Tm expected for the prey. When there was no positive control, a sample was considered a true positive if the three amplicon replicates had similar melting curves with the same sharp Tm. The presence of multiple peaks suggests that the PCR amplicons were heterogeneous, and/or possibly mixed with chimeras or primer dimers.

Statistical analysis

For each metabarcoding and Lazaro library, we have the number of species detected, the number of reads for each detected species, and for field samples, independent confirmation of each detection by MCA. We used MCA to classify true positive (TP) and false positive (FP or type I error) detections and true negative (TN) and false negative (FN or type II error) non-detections. We are considering: TP as detected prey species confirmed by MCA; FP as detected prey species not confirmed by MCA; TN as prey DNA not detected by MCA when the species was not detected by metabarcoding and Lazaro; FN as prey DNA detected by MCA when not detected by

metabarcoding and Lazaro. Prey species that were detected by MCA but were not detected by Lazaro, because it did not have a sequence in the respective DNA reference databases, were not considered FNs (the metabarcoding reference database was complete with all the species detected by MCA). For the control mock community, we did not need MCA to determine true and false positives and false negatives.

We calculated the theoretical limit of the detection (LOD) of MCA for all of the species with positive controls by estimating the amount of whole organism template that could be detected at a C_q=40, and calculating the upper 95% confidence interval of the geometric mean of the estimates. In addition, we estimated the amplification efficiency of the MCA primers to ensure it was high enough to amplify rare template sufficiently to detect. The limit of detection of MCA is quite low, but if there is little amount of prey DNA left in the gut, it is also possible that the prey sequence targeted by the species-specific primer pairs of MCA is absent, while other sequences are detected by metabarcoding and or Lazaro. If this were occurring, then for a given prey there should be a lower read count in both metabarcoding and Lazaro when the MCA is negative than when it is positive. We tested this with the prey species detected for which more than three true and false positives occurred in either the metabarcoding or Lazaro libraries. There were four and five such species for metabarcoding and Lazaro, respectively. Read numbers were *ln*-transformed and analyzed by the Welch *t*-test for unequal variance using the Welch-Satterthwaite equation to calculate degrees of freedom.

To compare the performance of metabarcoding and Lazaro, for each library, we also estimated [54,55]:

- *Sensitivity* (or true positive rate), which is the probability that a positive is detected: Sensitivity = TP/(TP+FN);

- *Specificity* (or true negative rate), which is the probability that a negative is not detected: Specificity = TN/(TN+TP)
- *False discovery rate* (FDR), which is the probability that a detection is a false positive: FDR = FP/(FP+TP)
- *False omission rate* (FOR), which is the probability that a non-detection is a false negative: FOR = FN/(FN+TN)
- *Accuracy*, which is the probability that detections and non-detections are correct: Accuracy = (TP+TN)/(TP+TN+FP+FN).

Higher sensitivity, specificity and accuracy, and lower false discovery rate and false omission rate are indicative of a better method. Using the aforementioned premises, we tested the following hypotheses:

H_1- Metabarcoding detects a higher number of true positive prey species than Lazaro because the reference database is larger. We tested this by comparing the number of initial detections in a library and the proportion of true positives after confirmation by MCA using a paired *t*-test with the 27 samples as independent observations, predicting that metabarcoding would have more true positives and a higher proportion of true detections;

H_2- Metabarcoding is more prone to false positive prey detections because of amplification bias and the larger reference database. We tested this by comparing the false discovery rate and specificity, predicting that metabarcoding would have a higher false discovery rate and a lower specificity;

H_3- Lazaro is more prone to generate false negatives because lacking the amplification of rare prey DNA fragments, it would be less sensitive. We tested this by comparing the false omission rate and sensitivity, predicting that Lazaro would have a higher false omission rate and lower sensitivity;

H_4- Lazaro enables prey detection with finer taxonomic resolution because the larger reference targets (e.g., mitogenomes) and higher sequencing depth would reduce ambiguity in species identifications. We tested this by comparing the taxonomic resolution of the final prey identifications.

H_5- The number of reads for both metabarcoding and Lazaro are positively related to the probability of a true positive across all prey species and to the relative template concentration for true positives within prey species. We tested the first part of this hypothesis using logistic regression of the *ln*-transform of the number of reads on the binomial variate indicating true positives by MCA versus false positives (logit link, binomial error) with Anova in the package car (Wald type II chi-square) and glm in Base *R*. There were 109 observations for metabarcoding and 116 observations for Lazaro, and no significant overdispersion for either regression. We tested the second part of this hypothesis, i.e., the number of reads is related to the amount of prey DNA in a sample, using the estimated relative template concentration from the qPCR for prey species with at least five true positive detections and variation in both variables of a least 0.5 order of magnitude. This was tested within prey species because amplification efficiency, baselines, and thresholds would be constant for the qPCR. There were three and two species tested for metabarcoding and Lazaro respectively. We calculated the relative initial template concentration from the qPCR amplification curves using LinRegPCR (version 2017.1) with the estimated mean PCR efficiency for each primer pair [56]. Relative initial template concentrations were $\log_{10}$ transformed, number of reads was *ln*-transformed, and data were analyzed with Pearson correlation coefficients using the Fisher transformation to estimate *p*-values.

Results and Discussion

Prey detections from the control mock community

In the control mock community, none of the samples of predators without any prey (controls) had any prey detected for either males or females for either metabarcoding or Lazaro. This indicates that there was no detectable extraneous DNA that contaminated the samples during the extraction process.

Only Lazaro detected prey species in the control mock community (Table 1; $Accuracy_{female}$=0.71, $Accuracy_{male}$=0.43). For some prey species, only one sex detected it and with few reads (n=2). Although theoretically ecoPCR [37] produced amplicons for the target prey species (maximum number of mismatches allowed per primer: -e=2), and the metabarcoding reference database contained their 16S sequences, none of the prey were detected ($Accuracy_{female}$=$Accuracy_{male}$=0), possibly due to the mismatches in at least one primer of the pair (Fig. S5) and preferential amplification of the more abundant predator DNA in the samples. Only the predator was detected by metabarcoding. This illustrates that the insufficient primer universality among taxa can preclude the detection (reduced sensitivity) of expected prey species.

Both metabarcoding and Lazaro did not detect any false positives, despite the use of comprehensive reference databases, which increases the likelihood of detecting false positives. In summary, both metabarcoding and Lazaro generated false negatives, with more false negatives and fewer true positives by metabarcoding than Lazaro. In addition, under controlled conditions, neither generated false positives. These results suggest that neither method on its own will detect all of the prey in a predator gut sample, but that Lazaro may sometimes be more accurate.

Prey detections from field sampled predators

After quality control, the 27 metabarcoding samples (predator guts or libraries) had an average of 2,964,430 reads (12% CV) and the 27 Lazaro samples had an average of 5,504,578 reads (14% CV). The presence of the DNA of each prey species in a predator gut sample was confirmed by

MCA [48]. Examples of the various positive and negative prey confirmations by MCA are illustrated in Fig. 1. In most of the cases, the differentiation of a true and false prey detection was performed by observing the presence or absence of the sample Tm corresponding or not with the Tm of the positive control. For example, *So. invicta* true positive control had a Tm of 76.5°C, and false positives had Tm's at 77.9, 79.2, 82.6 and 84.0°C. The theoretical LODs for detection by qPCR amplification were less than 1 pg of whole organism DNA per technical replicate for 28 of the 31 detected prey species, and for 26 of these species it was less than 0.1 pg of whole organism DNA/technical replicate (Table S4). As the DNA templates for MCA are only a small proportion of the whole organism DNA, the LODs indicate that MCA was very sensitive and unlikely to return a false negative for the majority of prey species examined. The amplification efficiency varied from 1.893-1.997 for all of the species-specific primer pairs, which should result in sufficient amplicons when the template is rare to detect by MCA. Nevertheless, 3 of the 31 detected prey species had high LOD: *Selenophorus alternans* (LOD=1.652 pg/technical replicate); *Euschistus heros* (LOD=3.245 pg/technical replicate); and *Cardiocondyla obscurior* (LOD=22.59 pg/technical replicate). Although unlikely, MCA could give a false negative in the case that prey DNA was so scarce that there was no MCA template in the sample. In this case, we reasoned that if MCA returned false negatives then the number of reads associated with MCA negatives should have a smaller number of reads than for MCA positives within a prey species. However, only one species (*Ph. tristis* for Lazaro) had fewer reads associated with negative than with positive MCA detections (Figure S6), indicating that false negative MCA detections were generally not a problem.

Initially, 30 prey were identified, all to species level, by both methods combined prior to verification by MCA (Tables 3 and S5). They were six species of Heteroptera, 10 Hymenoptera (Formicidae), five Coleoptera, three Lepidoptera, two Dermaptera, and a single species of Diptera, Orthoptera, Isoptera and Annelida. Metabarcoding and Lazaro initially detected a similar number

of prey species (26 [87%] and 21 [81%], respectively, with 16 species in common), but metabarcoding resulted in more species detections per sample than Lazaro (7.85±0.63 versus 6.78±0.42, respectively, t_{26}=2.08, *p*-value=0.0479) (Table 3).There were 212 prey detections in the metabarcoding samples, with *ln*-number of reads averaging 7.66 (range 0 to 14.44), and 183 prey detections in the Lazaro samples, with *ln*-number of reads averaging 2.90 (range 0.69 to 8.39). Out of 30 prey species initially detected, 17 species (57%) were confirmed by MCA as prey of the five epigeal predators (Table S5; 14 species by metabarcoding and 13 by Lazaro, with 10 species in common, i.e., 59% of confirmed prey species). Ten of the 13 species not confirmed by MCA were false positives and three species (*Atta sextans*, *Neomegalotomus parvus* and *Strongygaster triangulifera*) were not tested (Table S5). Of the 10 false positive species, five were detected by Lazaro in 15 prey detection instances (8.2% of initial detections), and seven were detected by metabarcoding in 21 prey detection instances (9.9% of initial detections). Most of these false positives were not amplified by MCA or the replicates did not have a consistent Tm. In a few cases the replicates had a consistent Tm, but at the wrong temperature. For example, all of the MCA replicates for the false detections of *M. persicae* gave a consistent signal with a sharp peak, but the peak was >2°C lower than the Tm of the true positive control (Fig. 1). This kind of false positive might have resulted from the taxonomic overclassification [57]. When the prey species is not in the reference database, a close-related species may be identified, instead of the more accurate determination at a higher taxonomic level. These would give false positive species identifications because they were identified beyond the resolution limitation of a reference database. Indeed, the metabarcode amplicon for the false detections of *M. persicae* also had high similarity with four other species of Macrosiphonini, the aphidid tribe of *M. persicae*. Hence, some false positives might be a true positive prey with a false species determination.

Another possible reason for false positives is contamination of the samples after DNA extraction. This could occur during any of the post-extraction procedures, such as library preparation and sequencing. If such contamination had occurred, both metabarcoding and Lazaro would detect the contaminant with substantial numbers of reads, but MCA would not detect it, because the contaminant would not be in the original DNA extracted sample. For example, many of the false positive detections of the coleopterans *Anthonomus grandis* (Table S5, 19 samples, 47,626 metabarcoding amplicons, 142 Lazaro reads) and *Harmonia axyridis* (Table S5, 8,919,830 metabarcoding amplicons, 18 samples, 1,190 Lazaro reads) had these characteristics, and may be post-extraction contaminants. False positive detections can also occur when the prey species was closely related to the predator. An example was the false detection by metabarcoding of *So. richteri* in the gut content of the predator *So. substituta* (Table S5). It may be difficult with this method to resolve taxonomically closely related species.

Regarding false negative prey detection, there were 11 false negatives for metabarcoding and 11 false negatives for Lazaro (Table 4). For example, *M. spectabilis* was not detected as prey by metabarcoding in two samples, and *Sp. frugiperda* was not detected as prey using Lazaro in two samples (Table S5), but they were detected by MCA. False negatives could have been generated for rare prey in the samples with a large number of pooled individuals (Table 5). For example, for the Lazaro samples, coverage ranged from 20,000 to 120,000 reads per individual, and the number of detected prey reads was only 1.3 to 29.2/individual. Thus, rare prey may be missed (false negative) in the Lazaro samples. The metabarcoding samples had coverage ranging from 11,000 to 64,000 amplicons/individual, and the number of detected prey amplicons ranged from 114 to 40,000/individual. Thus, rare prey may have been missed because of the large numbers of individuals in a sample. However, as the three ant species are known to recruit large numbers of individuals to harvest prey, rare prey are unlikely to occur in only one individual. Moreover, if

false negatives were related to missing rare prey, then the false omission rate, FN/(FN+TN), should be negatively correlated with coverage or prey detection. This was not observed (Tables 5 and S6), hence, while some rare prey may have been missed, they were equally likely to have been missed by both metabarcoding and Lazaro.

Species identifications have been demonstrated to differ when using different DNA extraction protocols, DNA polymerases, amplification parameters, reference databases or barcodes, and even when using different primers from the same barcode [9,13,58-61], and the results from our control mock community also show that different DNA-based detection methods differed in the species identified. So, the incongruent prey species detection between metabarcoding and Lazaro may not be unusual and additional possible reasons are discussed below. Nonetheless, the underlying consequence is that the ecological inferences are likely to be affected by the prey detection method used as the predator food webs would have different structures (Fig. 2). Specifically, the food webs of three of the predators would be different using only metabarcoding or Lazaro. These results highlight the need of precaution when comparing the data between eDNA studies to enable robust ecological comparisons [62,63].

OTU analysis could be conducted on the reads that did not match satisfactorily with any species in the DNA reference databases to complement the prey diversity analysis. The number of 'unassigned' reads was fairly high in both methods, but not surprisingly, more prominent in Lazaro. The percent of reads of confirmed prey detections across all 27 predator samples was 45% for metabarcoding and less than 1% for Lazaro. The majority of the 'unassigned' reads were related to the predator DNA (e.g., nuclear DNA), even though we reduced the amount of predator biomass by gut dissection or gaster removal (for ants). Another part of the 'unassigned' reads could be related to predator symbionts or parasites or other exogenous species that were not present in the reference databases used in this work. Although high, the 99% of 'unassigned' reads for the Lazaro

is not unexpected for two reasons: we only worked with mitochondrial reads and only for the arthropod species preyed upon by the predator, but not the predator mitochondrial reads. It is known that the proportion of mitochondrial reads obtained in NGS sequencing of whole macerated organisms or tissues without *in vitro* mitochondrial enrichment is only about 1% [19,29]. The predator mitochondrial reads should not be considered 'unassigned' reads in the strict sense, nevertheless they were not included in the assigned reads because there is no means to differentiate the predator reads with reads from a cannibalized conspecific. In a similar way, the prevalence of 55% of 'unassigned' reads for the metabarcoding data was not unexpected because no specific predator blocking primers were used to preclude or minimize amplification of the predator template. In metabarcoding, no parts of the predator genome were sequenced, except 16S barcode amplicons. As for Lazaro, the predator 16S amplicons were not considered 'assigned' reads so approximately half of the amplicons sequenced belonged to the predator. Our choice to not use specific predator blocking primers was based on Piñol et al. [64], who demonstrated that predator blocking primers are may coblock the amplification of prey species closely related to the predator.

Metabarcoding versus Lazaro

To compare which method, metabarcoding or Lazaro, resulted in better prey determination, we evaluated the accuracy of prey determination for feeding bioassay controls and field sampled predators. In addition, for the field sampled predators we determined the relation between the number of reads for a prey and the amount of prey DNA in the samples (feeding bioassay controls were not analyzed this way because metabarcoding did not detect prey reads). Specifically, we tested the five hypotheses discussed below.

H_1- Metabarcoding detects more true positive prey species than Lazaro. Following statistical comparisons of the field sampled predators, metabarcoding and Lazaro had a similar

number of confirmed prey per sample (1.81±0.24 and 1.85±0.23, respectively, t_{26}=-0.44, *p*-value=0.6632) and of the initial prey tested by MCA, the same false discovery rate (FP/(TP+FP) (Table 6). One might argue that the use of multiple barcode sequences would have expanded the detection of true positives by metabarcoding [39], however it might also have expanded the detection of false positives. The rejection of H_1 was corroborated by the results from the control mock community.

H_2- Metabarcoding is more prone to false positive prey detections than Lazaro. The false discovery rate for metabarcoding was 0.82±0.03 and Lazaro it was 0.64±0.03 (*p*-value=3.62E-05, Table 6). Metabarcoding and Lazaro had similar specificity (TN/(FP+TN), Table 6), so H_2 was rejected. While it was true that metabarcoding detected on average 350 times more true prey reads than Lazaro (Table S5), this did not convert into a higher detection of true positive or a lower detection of false positive prey detections compared to Lazaro.

H_3- Lazaro is more prone to have false negatives than metabarcoding. For the field sampled predators, false omission rate (FN/(FN+TN)) for Lazaro was similar to that for metabarcoding (Table 6). Similarly, metabarcoding did not have higher sensitivity (TP/(TP+FN)) than Lazaro (Table 6), so H_3 was rejected. The rejection of H_3 was corroborated by the results from the control mock community. A factor that may have contributed to false negative detections in some predators is that prey DNA is in an advanced state of degradation, precluding PCR amplification (in the case of metabarcoding) or being excluded by size selection during library construction, but still possible to be detected by MCA because of the smaller length of the target amplicon (between 100 to 200 bp, Table S3). In the case of metabarcoding, it could also be related to insufficient complementarity between template and metabarcoding primers, precluding the representation of a prey species or taxonomic group in the sample, or preferential amplification of the more common predator DNA, resulting in poor amplification of prey DNA. Realistically, it is quite likely that the number of false

negatives might be even higher, because we could not check all species co-occurring in the sample area because we could not be sure that all species were in the reference databases.

With H_1, H_2 and H_3 rejected, it follows that metabarcoding and Lazaro had similar accuracy in prey detection (Table 6). For the field sampled predators, accuracy for Lazaro was 0.67±0.02 and for metabarcoding it was 0.64±0.03 (p-value=0.4646). This differs from the results from the control mock community and in Srivathsan et al. [26]. They compared the efficacy of metabarcoding and Lazaro (referred to as metagenomics) to identify diet composition by fecal analysis (host plant chloroplasts) of two red-shanked doucs langurs (*Pygathrix nemaeus*) fed with a known diet. While metabarcoding detected 34% of the diet composition, Lazaro detected 50% of the known diet plus an unexpected species that was later confirmed to be in the diet.

H_4- Lazaro enables prey detection with finer taxonomic resolution than metabarcoding. For the field sampled predators, all confirmed species identifications were at the species level for both metabarcoding and Lazaro. Thus, in our field samples, taxonomic resolution was the same, and H_4 was rejected.

H_5- The number of reads for both metabarcoding and Lazaro are positively related to the probability of a true positive across all prey species and to the relative template concentration for true positives within prey species. Logistic regression showed that the probability of the true positive was not related to the number of reads for metabarcoding (regression coefficient =0.11±0.07, χ^2 =2.52, p-value=0.1122) but was highly positively related to the number of reads for Lazaro (regression coefficient =0.49±0.12, χ^2 =15.87, p-value=6.798E-5). For a prey species in the field samples of predators, the tests determine if the number of reads is correlated with the amount of prey in the predator, as measured by qPCR (Table 7). For Lazaro H_5 was accepted as there was a positive correlation between the number of reads and the relative template concentration in the samples for both species that could be analyzed. However, for metabarcoding H_5 was rejected for

two of the species as there was no correlation between the number of reads and the relative template concentration for any of the three species and accepted for one species. The interpretation of read numbers from the metabarcoding results has been controversial [65-70], and our results provide some support for the argument that the number of reads is an unreliable predictor of the DNA quantity in a metabarcoding sample but is a good predictor of prey DNA quantity in a Lazaro sample.

In terms of cost, metabarcoding has the potential to cost half that of Lazaro. In this study, we chose not to multiplex the 27 samples for metabarcoding analysis to keep the coverage per sample similar between methods. The costs that were the same for both methods were: sample preparation (USD10); total DNA extractions (USD100); library construction (USD85.50/each) and HiSeq4000 sequencing lane (USD4,310). For metabarcoding, there were additional costs for primer synthesis, PCR reactions for each sample, PCR reaction purifications and quantifications for each sample, which were estimated to be USD160. If we had multiplexed the 27 purified sample amplicons in one library, the total cost of metabarcoding would have been USD2,510.50. For the Lazaro method, samples cannot be multiplexed and the total cost was USD4,573.50.

Conclusions

Metabarcoding and Lazaro identified a range of prey species that were preyed upon by arthropod epigeal predators, but they were partially complementary methods sharing 87% of true positive detections (Table S5). Both methods crucially depend on the comprehensiveness of their respective DNA reference databases, which for metabarcoding is undeniably larger. Even so, Lazaro determined prey with similar specificity, sensitivity, accuracy and taxonomic resolution as metabarcoding. The use of multiple barcodes in the metabarcoding analysis could render higher sensitivity, although it could also increase false positives (reduce specificity) as each primer pair

carries its own associated bias. One may prefer Lazaro because it preserves the original sample DNA community, enabling further search for other targets (host plants, symbionts, parasites, etc), using any other DNA reference database and the number of reads was associated with the quantity of prey DNA in the predators. For metabarcoding, prey detection is constrained by the initial chosen barcodes. However, one may prefer metabarcoding because it is remains less expensive than Lazaro for processing a large set of samples as they can be multiplexed in a single library. In addition, it is easier to enrich a reference database by elucidating barcode sequences, which usually are fragments of a gene, than elucidating an organellar genome.

Availability of Supporting Data and Materials

Library sequencing datasets were deposited at GenBank and their Sequence Read Archive (SRA) access codes are in Supporting Information 1.

Additional Files

Supporting Information 1 (Word file): Figures S1 to S5.

Supporting Information 2 (Excel file): Tables S1 to S6.

List of abbreviations

Abbreviations used in the text are defined in the text at first use.

Consent for publication

Not applicable.

Competing interests

The authors declare that they have no competing interests.

Funding

This work was funded by the grant USDA-NIFA 2016-67030-24950.

Author’s contributions

Design of study: DPP, DAA, RMP, MRB
Collection and preparation of samples: SKAB, RMP, DPP
Data analyses (bioinformatic, qPCR, statistical): RCT, DPP, DAA
Writing of the manuscript: DPP, DAA, RCT, RMP

Acknowledgements

We would like to thank Jian Chen, Micky Eubanks, Hannah Gray, Fangneng Huang and Michael Strand for the donation of identified specimens to use as positive controls for MCA in qPCR analysis.

References

[1] Taberlet P, Coissac E, Pompanon F, Brochmann C, Willerslev E. Towards next-generation biodiversity assessment using DNA metabarcoding. Molecular ecology. 2012 Apr;21(8):2045-50.

[2] Clare EL. Molecular detection of trophic interactions: emerging trends, distinct advantages, significant considerations and conservation applications. Evol. Appl. 2014 7(9):1144-57.

[3] Paula DP. Next-generation sequencing and its impacts on entomological research in ecology and evolution. Neotrop. Entomol. 2021.

[4] Schloss PD, Westcott SL, Ryabin T, et al. Introducing mothur: open-source, platform-independent, community-supported software for describing and comparing microbial communities. Appl Environ Microbiol. 2009; 75:7537-7541.

[5] Caporaso JG, Kuczynski J, Stombaugh J, et al. QIIME allows analysis of high-throughput community sequencing data. Nat Methods 2010; 7:335-336.

[6] Boyer F, Mercier C, Bonin A, Le Bras Y, Taberlet P, Coissac E. Obitools: a unix-inspired software package for DNA metabarcoding. Mol Ecol Resour. 2016; 16:176-182.

[7] Callahan BJ, McMurdie PJ, Rosen MJ, Han AW, Johnson AJA, Holmes SP. DADA2: high-resolution sample inference from Illumina amplicon data. Nat Methods 2016; 13:581-583.

[8] Anslan S, Bahram M, Hiiesalu I, Tedersoo L. PipeCraft: flexible open-source toolkit for bioinformatics analysis of custom high-throughput amplicon sequencing data. Mol Ecol Resour. 2017;17(6):e234-e240.

[9] Taberlet P, Bonin A, Zinger L, Coissac E. Environmental DNA: for biodiversity research and monitoring. Published to Oxford Scholarship. 272 p. Print ISBN-13: 9780198767220. 2018; doi:10.1093/oso/9780198767220.001.0001.

[10] Deagle BE, Jarman SN, Coissac E, Pompanon F, Taberlet P. DNA metabarcoding and the COI marker: not a perfect match. Biol Lett. 2014; 10: 20140562.

[11] Clarke LJ, Soubrier J, Weyrich LS, Cooper A. Environmental metabarcodes for insects: *in silico* PCR reveals potential for taxonomic bias. Mol Ecol Resour. 2014; 14:1160-1170.

[12] Elbrecht V, Leese F. Can DNA-based ecosystem assessments quantify species abundance? Testing primer bias and biomass-sequence relationships with an innovative metabarcoding protocol. PLoS One 2015; 10:e0130324.

[13] Elbrecht V, Leese F. Validation and development of COI metabarcoding primers for freshwater macroinvertebrate bioassessment. Front Environ Sci. 2017; 5:11.

[14] Haas BJ, Gevers D, Earl AM, et al. (2011). Chimeric 16S rRNA sequence formation and detection in Sanger and 454-pyrosequencing PCR amplicons. Genome Res. 2011; 21:494-504.

[15] Zhou X, Li Y, Liu S, et al. Ultra-deep sequencing enables high-fidelity recovery of biodiversity for bulk arthropod samples without PCR amplification. GigaScience 2013; 2:4.

[16] Gillett CP, Crampton-Platt A, Timmermans MJTN, et al. Bulk *de novo* mitogenome assembly from pooled total DNA elucidates the phylogeny of weevils (Coleoptera: Curculionoidea). Mol Biol Evol. 2014; 31:2223-2237.

[17] Tang M, Tan M, Meng G, Yang S, et al. Multiple, sequencing of pooled mitochondrial genomes - a crucial step toward biodiversity analysis using mito-metagenomics. Nucleic Acids Res. 2014; 42(22):e166.

[18] Andujar C, Arribas P, Ruzicka F, et al. Phylogenetic community ecology of soil biodiversity using mitochondrial metagenomics. Mol Ecol. 2015; 24:3603-3617.

[19] Crampton-Platt AL, Timmermans MJTN, Gimmel ML, et al. Soup to tree: the phylogeny of beetles inferred by mitochondrial metagenomics of a Bornean rainforest sample. Mol Biol Evol. 2015; 32(9):2302-2316.

[20] Gomez-Rodrıguez C, Crampton-Platt A, Timmermans MJ, et al. Validating the power of mitochondrial metagenomics for community ecology and phylogenetics of complex assemblages. Meth Ecol Evol. 2015; 6(8), 883-894.

[21] Liu S, Wang X, Xie L, et al. (2016) Mitochondrial capture enriches mito-DNA 100 fold, enabling PCR-free mitogenomics biodiversity analysis. Mol Ecol Resour. 2016; 16:470-479.

[22] Linard B, Crampton-Platt A, Timmermans MJTN, Vogler AP (2015) Metagenome skimming of insect specimen pools: potential for comparative genomics. Gen Biol Evol. 7(6):1474-1489.

[23] Shokralla S, Gibson J, King I, et al. Environmental DNA barcode sequence capture: targeted, PCR-free sequence capture for biodiversity analysis from bulk environmental samples. bioRxiv 2016.

[24] Sarmashghi, S., Bohmann, K., Gilbert, M.T.P., Bafna, V. & Mirarab, S. Skmer: assembly-free and alignment-free sample identification using genome skims. Genome Biol. 20(1), 1-20 (2019).

[25] Ji, Y., Huotari, T., Roslin, T., Schmidt, N. M., Wang, J., Yu, D. W., & Ovaskainen, O. (2020). SPIKEPIPE: A metagenomic pipeline for the accurate quantification of eukaryotic species occurrences and intraspecific abundance change using DNA barcodes or mitogenomes. Mol Ecol Resour. 20(1), 256-267.

[26] Srivathsan A, Sha JCM, Vogler AP, Meier R. Comparing the effectiveness of metagenomics and metabarcoding for diet analysis of a leaf feeding monkey (*Pygathrix nemaeus*). Mol Ecol Resour. 2015; 15:250-261.

[27] Srivathsan A, Ang A, Vogler AP, Meier R. Fecal metagenomics for the simultaneous assessment of diet, parasites, and population genetics of an understudied primate. Front Zool. 2016; 13:17.

[28] Paula DP, Linard B, Andow DA, Sujii ER, Pires CSS, Vogler AP. Detection and decay rates of prey and prey symbionts in the gut of a predator through metagenomics. Mol Ecol Resour. 2015; 15:880-892.

[29] Paula DP, Linard B, Platt AC, Srivathsan A, Timmermans M, Sujii E, Pires C, Machado L, Andow DA, Vogler A. Uncovering trophic interactions in arthropod predators through DNA shotgun-sequencing of gut contents. PLoS ONE 2016; 11:E0161841.

[30] Aquino AM, Aguiar-Menezes EL, Queiroz JM. Recomendações para coleta de artrópodes terrestres por armadilhas de queda ("pitfall-traps"). Circular Técnica. 16. Embrapa. Rio de Janeiro. 2006; 8p.

[31] Sutherland WJ. Ecological census techniques: a handbool. Cambridge: Cambridge University, 1996. 336 p.

[32] Greenstone MH, Weber DC, Coudron TA, Payton ME, Hu JS. Removing external DNA contamination from arthropod predators destined for molecular gut-content analysis. Mol Ecol Resour. 2012, 12(3):464-9.

[33] Elbrecht, V., Taberlet, P., Dejean, T., Valentini, A., Usseglio-Polatera, P., Beisel, J. N., ... Leese, F. (2016). Testing the potential of a ribosomal 16S marker for DNA metabarcoding of insects. Peer Journal, 4, e1966.

[34] Sousa LL, Silva SM, Xavier R (2019). DNA metabarcoding in diet studies: unveiling ecological aspects in aquatic and terrestrial ecosystems. Environmental DNA 2019; 1(3):199-214.

[35] O'Donnell JL, Kelly RP, Lowell NC, Port JA (2016) Indexed PCR Primers Induce Template-Specific Bias in Large-Scale DNA Sequencing Studies. PLoS ONE 11(3): e0148698.

[36] Juen A, Traugott M. (2006) Amplification facilitators and multiplex PCR: Tools to overcome PCR-inhibition in DNA-gut-content analysis of soil-living invertebrates. Soil Biol Biochem 38(7):1872-1879.

[37] Ficetola GF, Coissac E, Zundel S, Riaz T, Shehzad W, Bessière J, et al. An *in silico* approach for the evaluation of DNA barcodes. BMC Genomics 2010;11:e434.

[38] Stoesser G, Moseley M A, Sleep J, McGowran M, Garcia-Pastor M, Sterk P, The EMBL Nucleotide Sequence Database. Nucleic Acids Res. 1998; 26:8-15.

[39] De Barba M, Miquel C, Boyer F, Mercier C, Rioux D, Coissac E, Taberlet P. DNA metabarcoding multiplexing and validation of data accuracy for diet assessment: application to omnivorous diet. Mol Ecol Resour. 2014; 14:306-323.

[40] Quéméré E, Hibert F, Miquel C, Lhuillier E, Rasolondraibe E, Champeau J, et al. (2013) A DNA metabarcoding study of a primate dietary diversity and plasticity across its entire fragmented range. PLoS ONE. 2013, 8(3):e58971.

[41] Paula, DP; Timbó, RV; Togawa, RC; Vogler, AP; Andow, DA (2021) Quantitative prey species detection in predator guts across multiple trophic levels by DNA shotgun sequencing. Submitted to MEE. bioRxiv preprint: doi: https://doi.org/10.1101/2021.04.01.438119.

[42] Andrews, D. (2010) FastQC: a quality control tool for high throughput sequence data. Available online at: http://www.bioinformatics.babraham.ac.uk/projects/fastqc

[43] Aronesty E. ea-utils: Command-line tools for processing biological sequencing data. 2011; URL https://github. com/ExpressionAnalysis/ea-utils.

[44] Martin M. Cutadapt removes adapter sequences from high-throughput sequencing reads. EMBnet.journal 2011; 17(1):10-12.

[45] Shen W, Le S, Li Y, Hu F. SeqKit: a cross-platform and ultrafast toolkit for FASTA/Q file manipulation. PLoS ONE 2016; 11(10):e0163962.

[46] Ririe KM, Rasmussen RP, Wittwer CT. Product differentiation by analysis of DNA melting curves during the polymerase chain reaction. Anal Biochem 1997; 245:154-160.

[47] Zhang T, Fang HH (2005). 16S rDNA clone library screening of environmental sample using melting curve analysis. Journal of the Chinese Institute of Engineers 2005; 28:1153-1155.

[48] Winder L, Phillips C, Richards N, Ochoa-Corona F, Hardwick S, Vink CJ, Goldson S. Evaluation of DNA melting analysis as a tool for species identification. Methods Ecol Evol. 2011; 2:312-320.

[49] Perera OP, Allen KC, Jain D, Purcell M, Little NS, Luttrell RG. Rapid Identification of *Helicoverpa armigera* and *Helicoverpa zea* (Lepidoptera: Noctuidae) Using Ribosomal RNA Internal Transcribed Spacer 1. J Insect Sci. 2015; 15(1):155.

[50] Paula DP, Andow DA Melting curve analysis for detection and identification of ghost parasitoids in host carcasses a month after host death. Methods Ecol Evol 2021; 12(9):1552-1561.

[51] Kearse M, Moir R, Wilson A, Stones-Havas S, Cheung M, Sturrock S, Buxton S, Cooper A, Markowitz S, Duran C, Thierer T, Ashton B, Meintjes P, Drummond A. Geneious Basic: an integrated and extendable desktop software platform for the organization and analysis of sequence data. Bioinformatics 2012; 28(12):1647-1649.

[52] Ye J, Coulouris G, Zaretskaya I, Cutcutache I, Rozen S, Madden T. Primer-BLAST: A tool to design target-specific primers for polymerase chain reaction. BMC Bioinformatics 2012; 13:134.
[53] R Core Team. R: A language and environment for statistical computing. R Foundation for Statisical Computing, Vienna, Austria, 2019.
[54] Altman DG, Bland JM (June 1994). "Diagnostic tests. 1: Sensitivity and specificity". BMJ. 308 (6943): 1552.
[55] Fletcher RH, Fletcher SW, Fletcher GS. Clinical epidemiology: the essentials (4th ed.). Baltimore, Md.: Lippincott Williams & Wilkins. 2005; pp. 45. ISBN 0-7817-5215-9.
[56] Ruijter JM, Ramakers C, Hoogaars WMH, Karlen Y, Bakker O, van den Hoff MJB, Moorman AFM Amplification efficiency: linking baseline and bias in the analysis of quantitative PCR data. Nucleic Acids Res. 2009; 37: e45.
[57] Richardson RT, Bengtsson-Palme J, Johnson RM. Evaluating and optimizing the performance of software commonly used for the taxonomic classification of DNA metabarcoding sequence data. Mol Ecol Resour. 2017; 17:760-769.
[58] Meusnier I, Singer GA, Landry JF, Hickey DA, Hebert PD, Hajibabaei M. A universal DNA mini-barcode for biodiversity analysis. BMC Genomics 2008; 9:214.
[59] Leray M, Yang JY, Meyer CP, Mills SC, Agudelo N, Ranwez V, et al. A new versatile primer set targeting a short fragment of the mitochondrial COI region for metabarcoding metazoan diversity: application for characterizing coral reef fish gut contents. Front Zool. 2013; 10(1):34.
[60] Gibson J, Shokralla S, Porter TM, King I, van Konynenburg S, Janzen DH, Hallwachs W, Hajibabaei M. Simultaneous assessment of the macrobiome and microbiome in a bulk sample of tropical arthropods through DNA metasystematics. PNAS 2014; 111(22):8007-8012.
[61] Nichols RV, Vollmers C, Newsom LA, Wang Y, Heintzman PD, Leighton M, Green RE, Shapiro B. Minimizing polymerase biases in metabarcoding. Mol Ecol Resour. 2018; 18:927-939.
[62] Nilsson RH, Tedersoo L, Lindahl BD, et al. Towards standardization of the description and publication of next-generation sequencing datasets of fungal communities. New Phytologist 2011; 191:314-318.

[63] Tedersoo L, Ramirez KS, Nilsson RH, Kaljuvee A, Kõljalg U, Abarenkov K. Standardizing metadata and taxonomic identification in metabarcoding studies. GigaScience 2015; 4(1):s13742-015-0074-5.

[64] Piñol J, San Andrés V, Clare EL, Mir G, Symondson WO. A pragmatic approach to the analysis of diets of generalist predators: The use of next-generation sequencing with no blocking probes. Mol Ecol Resour. 2014, 14(1):18-26.

[65] Deagle, B. E., Thomas, A. C., McInnes, J. C., Clarke, L. J., Vesterinen, E. J., et al. Counting with DNA in metabarcoding studies: how should we convert sequence reads to dietary data? Mol Ecol. 28, 391-406 (2019).

[66] Piñol, J., Mir, G., Gomez-Polo, P. & Agustí, N. Universal and blocking primer mismatches limit the use of high throughput DNA sequencing for the quantitative metabarcoding of arthropods. Mol Ecol Resour. 15, 1-12 (2015).

[67] Piñol, J., Senar, M. A., & Symondson, W. O. The choice of universal primers and the characteristics of the species mixture determines when DNA metabarcoding can be quantitative. Mol Ecol. 28, 407-419 (2018).

[68] Thomas, A. C., Deagle, B. E., Eveson, J. P., Harsch, C. H., & Trites, A. W. Quantitative DNA metabarcoding: improved estimates of species proportional biomass using correction factors derived from control material. Mol Ecol Resour. 16(3), 714-726 (2016).

[69] Bista, I., Carvalho, G. R., Tang, M., Walsh, K., Zhou, X., Hajibabaei, M., ... Creer, S. Performance of amplicon and shotgun sequencing for accurate biomass estimation in invertebrate community samples. Mol Ecol Resour. 18, 1020-1034 (2017).

[70] Lamb, P. D., Hunter, E., Pinnegar, J. K., Creer, S., Davies, R. G., & Taylor, M. I. How quantitative is metabarcoding: A meta-analytical approach. Mol Ecol. 28(2), 420-430 (2019).

Table 1. Number of reads detected for the control mock community by mapping unassembled shotgun reads (Lazaro) and metabarcoding (16S barcode) using the harlequin *Harmonia axyridis* (48 h after adult emergence with no food) as predator and six prey species, consumed at once in an interval of one hour. Gut contents of the predators were analyzed after six hours after feeding on the last prey item. The threshold used for Lazaro was 99% identity in a minimum overlap of 140 bp and for metabarcoding was 98% identity for an amplicon between 180-230 bp. Mb: metabarcoding; L: Lazaro.

		**Predator**		**Prey**											
**Predator sex**	**Time (h) after feeding**	***Harmonia axyridis***		***Acyrthosiphon pisum***		***Aphis craccivora***		***Aphis glycines***		***Aphis gossypii***		***Myzus persicae***		***Cycloneda munda***	
		**Mb**	**L**	**Mb**	**L**	**Mb**	**L**	**Mb**	**L**	**Mb**	**L**	**Mb**	**L**	**Mb**	**L**
Female	-	114,033	18,984	0	0	0	0	0	0	0	0	0	0	0	0
	6 h	128,773±11,714.9	40,498±9,556.9	0	16	0	26	0	2	0	2	0		0	6
Male	-	130,314	35,340	0	0	0	0	0	0	0	0	0	0	0	0
	6 h	451,570.75±371,433.7	43,452±9,443.5	0	10	0	4	0	0	0	0	0	10	0	0

Table 2. Species detected as prey of epigeal arthropod predators by metabarcoding or Lazaro or both in at least one of 27 libraries before verification by Melting Curve Analysis (MCA) in qPCR. Species with the name in bold are the ones with detection confirmed by MCA in at least one library. All these species are likely to occur in the sampling area/period.

Order	Species (Family)	Detection method(s)	# reads	# libraries	Predator
Annelida	*Phascolosoma esculenta* (Phascolosomatidae)	Metabarcoding	21,938	13	*Dorymyrmex brunneus*, *Tetracha* sp.
Coleoptera	***Anthonomus grandis* (Curculionidae)**	Both	946,283	27	*Pheidole flavens*, *Dorymyrmex brunneus*, *Solenopsis substituta*, *Tetracha* sp., *Euborellia annulipes*
	Eriopis connexa (Coccinellidae)	Both	2,706	5	*Dorymyrmex brunneus*, *Solenopsis substituta*, *Tetracha* sp., *Euborellia annulipes*
	***Harmonia axyridis* (Coccinellidae)**	Both	12,800,160	27	*Pheidole flavens*, *Dorymyrmex brunneus*, *Solenopsis substituta*, *Tetracha* sp., *Euborellia annulipes*
	Selenophorus alternans (Carabidae)	Both	1,639,746	9	*Pheidole flavens*, *Dorymyrmex brunneus*, *Solenopsis substituta*, *Tetracha* sp.
	Tetracha brasiliensis (Carabidae)	Lazaro	2	1	*Solenopsis substituta*
Dermaptera	***Doru luteipes* (Forficulidae)**	Both	1,808	7	*Pheidole flavens*, *Dorymyrmex brunneus*, *Euborellia annulipes*
	***Euborellia annulipes* (Anisolabididae)**	Both	41,492	12	*Pheidole flavens*, *Dorymyrmex brunneus*, *Solenopsis substituta*, *Tetracha* sp.
Diptera	*Strongygaster triangulifera* (Tachinidae)	Both	62	2	*Tetracha* sp., *Euborellia annulipes*
Hemiptera	***Chinavia impicticornes* (Pentatomidae)**	Both	24,621	10	*Dorymyrmex brunneus*, *Solenopsis substituta*, *Tetracha* sp., *Euborellia annulipes*
	***Euschistus heros* (Pentatomidae)**	Both	267,209	5	*Dorymyrmex brunneus*, *Tetracha* sp.
	***Mahanarva spectabilis* (Cercopidae)**	Lazaro	12	2	*Solenopsis substituta*, *Tetracha* sp.
	Neomegalotomus parvus	Metabarcoding	365	1	*Tetracha* sp.
	Myzus persicae (Aphididae)	Metabarcoding	1,696	2	*Dorymyrmex brunneus*

	Planicephalus flavicosta (Cicadellidae)	Metabarcoding	367	1	*Dorymyrmex brunneus*
Hymenoptera	*Atta sextans* (Formicidae)	Both	2,291	6	*Dorymyrmex brunneus*
	Brachymyrmex patagonicus (Formicidae)	Lazaro	24	11	*Pheidole flavens*, *Dorymyrmex brunneus*, *Tetracha* sp., *Euborellia annulipes*
	***Cardiocondyla obscurior* (Formicidae)**	Both	5,576	3	*Pheidole flavens*, *Solenopsis substituta*
	***Dorymyrmex brunneus* (Formicidae)**	Both	3,829	15	*Pheidole flavens*, *Solenopsis substituta*, *Tetracha* sp., *Euborellia annulipes*
	***Pheidole flavens* (Formicidae)**	Both	244,820	15	*Dorymyrmex brunneus*, *Solenopsis substituta*, *Tetracha* sp., *Euborellia annulipes*
	***Pheidole obscurithorax* (Formicidae)**	Both	36	4	*Pheidole flavens*, *Dorymyrmex brunneus*
	***Pheidole oxyops* (Formicidae)**	Both	9,015,220	27	*Pheidole flavens*, *Dorymyrmex brunneus*, *Solenopsis substituta*, *Tetracha* sp., *Euborellia annulipes*
	***Pheidole tristis* (Formicidae)**	Both	2,795,078	27	*Pheidole flavens*, *Dorymyrmex brunneus*, *Solenopsis substituta*, *Tetracha* sp., *Euborellia annulipes*
	Solenopsis richteri (Formicidae)	Metabarcoding	75,556	1	*Solenopsis substituta*
	Solenopsis substituta (Formicidae)	Both	608	9	*Pheidole flavens*, *Dorymyrmex brunneus*
Isoptera	***Syntermes spinosus* (Termitidae)**	Metabarcoding	897	1	*Dorymyrmex brunneus*
Lepidoptera	***Chrysodeixis includens* (Noctuidae)**	Metabarcoding and Lazaro	45	1	*Tetracha* sp.
	Glena unipennaria (Geometridae)	Both	280	1	*Tetracha* sp.
	***Spodoptera frugiperda* (Noctuidae)**	Metabarcoding	8,448	11	*Dorymyrmex brunneus*, *Tetracha* sp.
Orthoptera	*Gryllus argentinus* (Gryllidae)	Metabarcoding	32	1	*Dorymyrmex brunneus*

Table 3. Number of prey species identified by metabarcoding and Lazaro before verification and proportion verified by Melting Curve Analysis (MCA) in qPCR. Mb: metabarcoding; L: Lazaro.

Library	Predator Species	Original			Proportion verified by MCA		
		Mb	L	Both	Mb	L	both
1	*Pheidole flavens*	6	6	5	0.50	0.40	0.50
2	*Pheidole flavens*	5	5	4	0.50	0.50	0.67
3	*Pheidole flavens*	6	6	5	0.20	0.25	0.25
4	*Pheidole flavens*	6	5	5	0.40	0.40	0.40
5	*Pheidole flavens*	5	6	4	0.33	0.33	0.33
6	*Pheidole flavens*	5	6	5	0.60	0.50	0.60
7	*Pheidole flavens*	4	6	4	0.33	0.25	0.33
8	*Pheidole flavens*	4	5	4	0.00	0.00	0.00
9	*Pheidole flavens*	5	7	4	0.67	0.50	0.67
10	*Pheidole flavens*	4	7	4	0.50	0.33	0.50
11	*Pheidole flavens*	4	6	4	0.50	0.25	0.50
12	*Pheidole flavens*	5	6	3	0.67	0.40	0.67
13	*Dolymyrmex brunneus*	9	6	6	0.43	0.50	0.50
14	*Dolymyrmex brunneus*	10	7	5	0.40	0.20	0.33
15	*Dolymyrmex brunneus*	8	4	4	0.33	0.50	0.50
16	*Dolymyrmex brunneus*	6	9	5	0.25	0.33	0.33
17	*Dolymyrmex brunneus*	10	5	5	0.25	0.25	0.25
18	*Dolymyrmex brunneus*	11	6	5	0.29	0.50	0.50
19	*Dolymyrmex brunneus*	12	8	6	0.33	0.50	0.67
20	*Dolymyrmex brunneus*	10	8	5	0.00	0.00	0.00
21	*Dolymyrmex brunneus*	10	6	5	0.43	0.60	0.75
22	*Dolymyrmex brunneus*	8	8	5	0.00	0.00	0.00
23	*Dolymyrmex brunneus*	8	6	5	0.40	0.50	0.67
24	*Dolymyrmex brunneus*	13	6	5	0.13	0.33	0.33
25	*Solenopsis substituta*	12	11	8	0.50	0.57	0.60
26	*Tetracha* sp.	16	15	10	0.45	0.55	0.50
27	*Euborellia annulipes*	10	7	6	0.33	0.33	0.33
	**Average**	7.85	6.78	5.04	0.36	0.36	0.43
	**Standard Error**	0.63	0.42	0.26	0.04	0.03	0.04

Table 4. False negative and false positive species detected by metabarcoding, Lazaro or both. In bold are the species that did not have a mitogenome deposited at the GenBank.

	Metabarcoding	Lazaro	Both
**False negatives**	*Pheidole obscurithorax*	***Syntermes spinosus***	*Cardiocondyla obscurior*
			Chrysodeixis includens
			Mahanarva spectabilis
			Solenopsis richteri
			Spodoptera frugiperda
**False positives**	*Euschistus heros*	*Brachymyrmex patagonicus*	*Anthonomus grandis*
	Gryllus argentinus	*Pheidole obscurithorax*	*Cardiocondyla obscurior*
	Myzus persicae	*Solenopsis substituta*	*Doru luteipes*
	Phascolosoma esculenta	*Tetracha sp.*	*Dorymyrmex brunneus*
	Planicephalus flavicosta		*Eriopis connexa*
	Solenopsis richteri		*Glena unipennaria*
	Spodoptera frugiperda		*Harmonia axyridis*
			Pheidole oxyops
			Pheidole tristis
			Selenophorus alternans

Table 5. Coverage per individual predator and verified prey per individual in the field predator samples and false omission rate for each predator species.

Predator species	Number of samples	Individuals/ sample	Sample		Detected prey		False omission rate (FN/(FN+TN))	
			Metabarcoding amplicons/ individual	Lazaro reads/ individual	Metabarcoding amplicons/ individual	Lazaro reads/ individual	Metabarcoding	Lazaro
Dorymyrmex brunneus	12	100	29,644	55,046	11,797	4.2	0.073	0.068
Euborellia annulipes	1	46	64,444	119,665	114	9.1	0.250	0.250
Pheidole flavens	12	200	14,822	27,523	4,867	10.2	0.047	0.056
Solenopsis substituta	1	273	10,859	20,163	317	1.3	0.286	0.167
Tetracha sp.	1	49	60,499	112,338	39,600	29.2	0.200	0.167

Table 6. Sensitivity, specificity, false discovery rate, false omission rate and accuracy for prey determinations in field collected predators by metabarcoding and Lazaro, with paired *t*-test and *p*-value.

	**Sensitivity**	**Specificity**	**False discovery rate**	**False omission rate**	**Accuracy**
Metabarcoding	0.812 (0.058)	0.591 (0.030)	0.638 (0.035)	0.076 (0.020)	0.641 (0.027)
Lazaro	0.807 (0.058)	0.619 (0.022)	0.638 (0.033)	0.079 (0.020)	0.665 (0.021)
t_{26}	-0.1653	0.7149	-0.0231	0.2568	0.7422
p-value	0.8700	0.4810	0.9818	0.7994	0.4646

Table 7. Correlations between relative initial template concentration and *ln* number of read for true positives.

	r^2	***z*-score**	***p*-value**
**Metabarcoding**			
Harmonia axyridis	-0.306	-0.893	0.3718
Pheidole tristis	0.906	4.984	6.23E-07
Pheidole flavens	-0.270	-0.920	0.3577
**Lazaro**			
Harmonia axyridis	0.605	1.985	0.0472
Pheidole tristis	0.902	4.916	8.82E-07

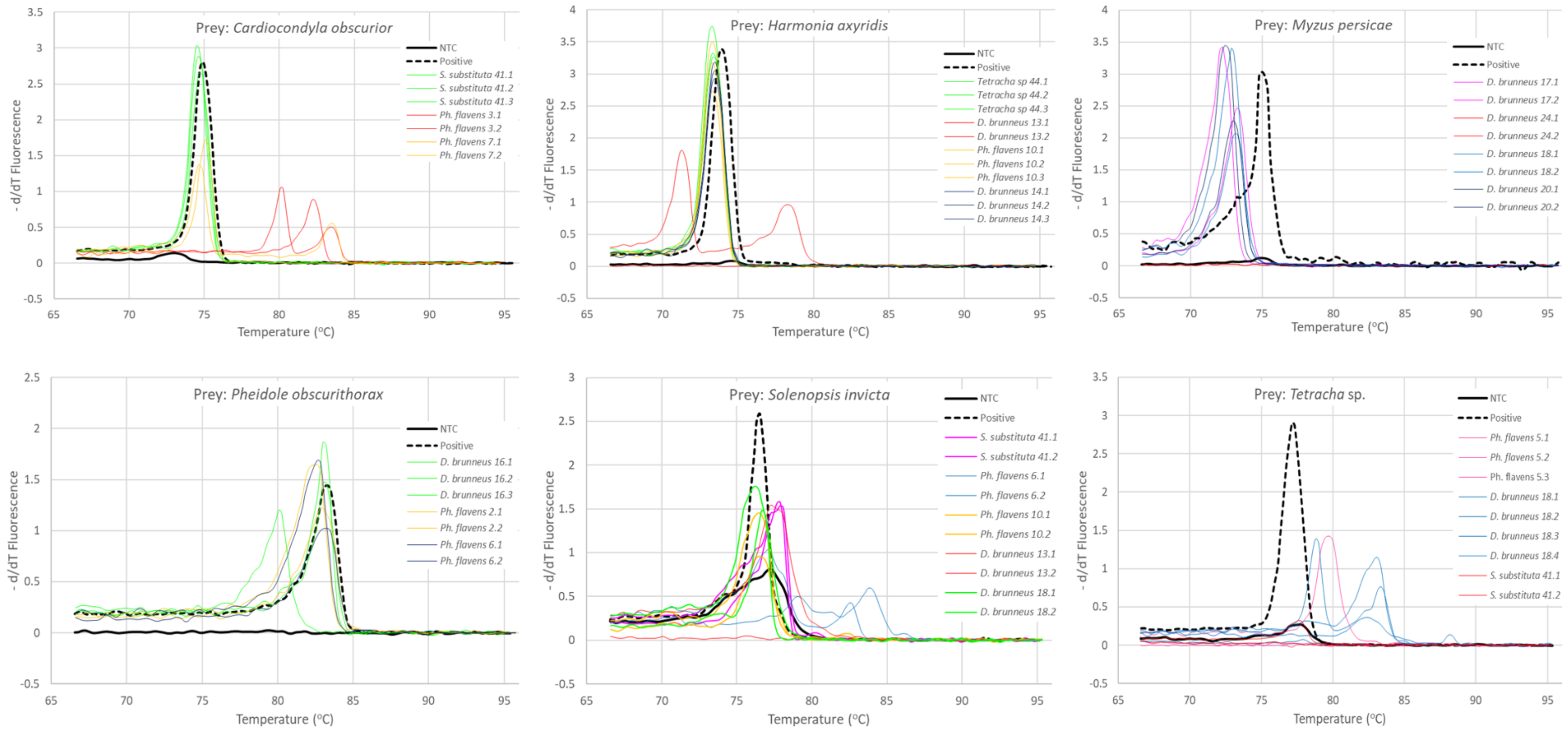

Fig. 1. Verification of prey detection by Melting Curve Analysis (MCA) in qPCR with positive controls and NTCs (no template controls). The graphs represent a melting curve for some prey detected by metabarcoding or mapping of unassembled shotgun reads (Lazaro) or both. Predator samples are informed at the side legend. Green, yellow and gray curves are positive identification of the indicated prey, and red, magenta and blue curves are negative identifications. Predator species, library identification and technical replicate number designate samples.

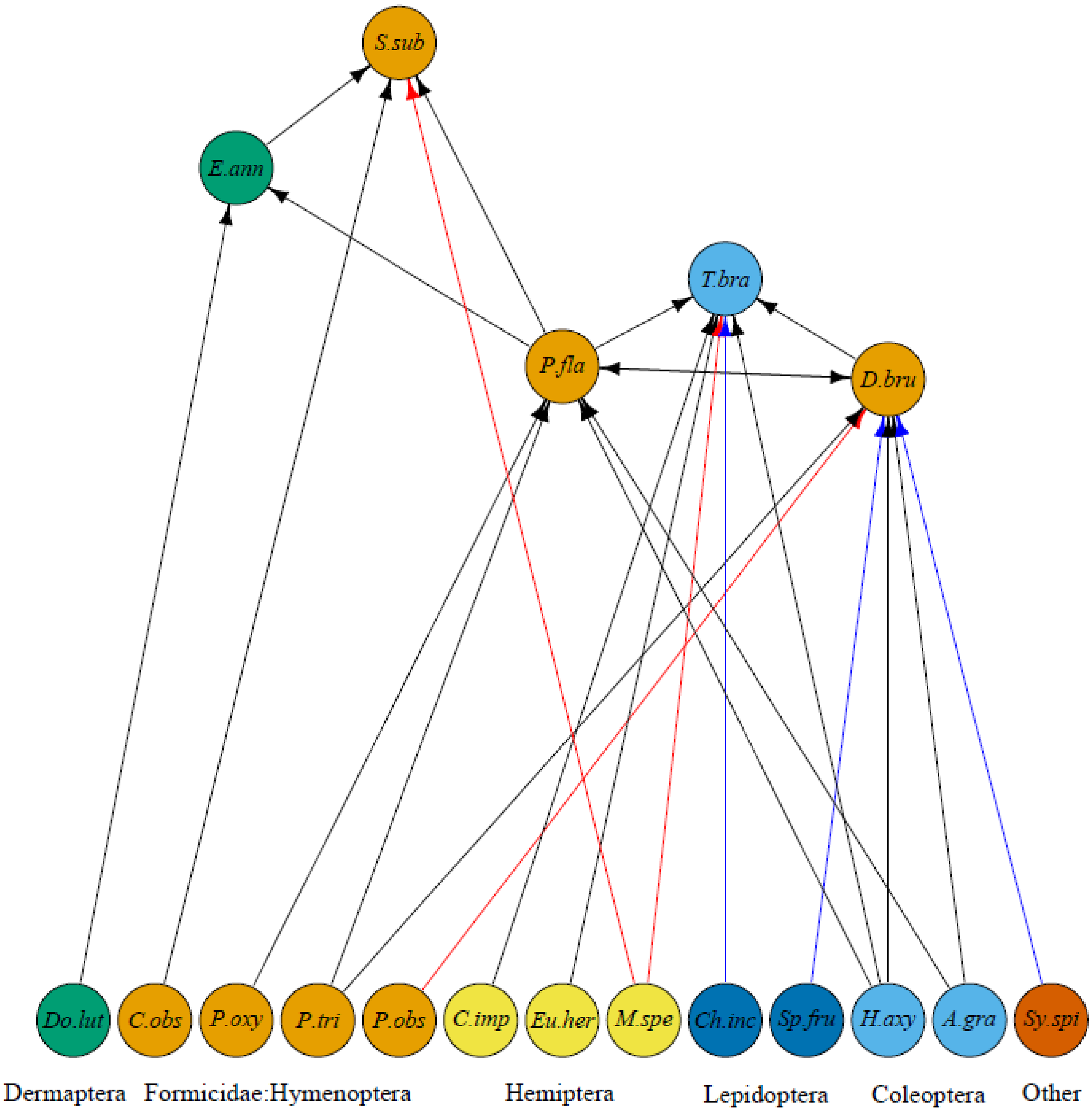

Fig. 2. Qualitative food web of the five epigeal predators (top of figure) detected by metabarcoding only (blue links), mapping of unassembled shotgun reads (Lazaro) only (red links), or both (black links) and confirmed by Melting Curve Analysis (MCA) in qPCR. Predation is indicated by the arrow direction. Height of species is the relative trophic level of the species. Predator species are *E.ann* = *Euborellia annulipes*; *S.sub* = *Solenopsis substituta*; *Ph.fla* = *Pheidole flavens*; *T.bra* = *Tetracha brasilensis*; *D.bru* = *Dorymyrmex brunneus*. Extra- or

intraguild prey are *Do.lut* = *Doru luteipes*; *C.obs* = *Cardiocondyla obscurior*; *Ph.oxy* = *Pheidole oxyops*; *Ph.tri* = *Pheidole tristis*; *Ph.obs* = *Pheidole obscurithorax*; *C.imp* = *Chinavia impicticornes*; *Eu.her* = *Euschistus heros*; *M.spe* = *Mahanarva spectabilis*; *Ch.inc* = *Chrysodeixis includens*; *Sp.fru* = *Spodoptera frugiperda*; *H.axy* = *Harmonia axyridis*; *A.gra* = *Anthonomus grandis*; *Sy.spi* = *Syntermes spinosus*.

Click here to access/download
Supplementary Material
Supporting information 1 GigaScience Sep 6 2021.docx

Click here to access/download
Supplementary Material
Supporting Information 2_Sep 6 2021.xlsx

Personal Cover
Click here to access/download;Personal Cover;Rebuttal letter GigaScience USDA AFRI ms Sep 6 2021.pdf

Dear Editor, Dr. Zauner,

Thank you very much for your assessment of the previous version of our manuscript GIGA-D-20-00345 and for sending us the reviewers' comments. We have substantially reformulated the manuscript and offer it as a resubmission, having added an experimental control, clarified the methodologies used, reanalyzed the metabarcoding data with a reference database that includes all of the main species sampled, and providing new information to justify the use of MCA (melt curve analysis) to validate the other methods.

We added an experimental control, which we refer to as a control mock prey community, as a methodological control for the other methods, which also acted as an extraction control, addressing two of the major concerns of the reviewers of our previous submission. The results from the control mock community experiment support the findings we reported for the field samples. We decided to replace the acronym DDSS (direct DNA shotgun sequencing) by the name 'Lazaro' because the previous acronym was not providing a complete representation of the method as it was not mentioning the mapping of unassembled reads, which is an important component, and it did not distinguish our method from other methods that rely on DDSS. We did not find a good acronym to represent the whole idea of "mapping unassembled shotgun reads", so decided to use the name Lazaro as it represents "resuscitation" of the prey DNA.

We also added to the metabarcoding reference database the 16S amplicon sequences from 43 potential prey species that occurred in the experimental plots that were not available at the time we performed the first analysis. These species included all of the predator species sampled and a few prey species that were present in the experimental fields. We extracted these sequences from the mitogenome sequences that we had previously deposited in GenBank. This addition resulted in considerably fewer false negatives and positives for metabarcoding. Now, in this updated resubmission of the manuscript, the results of the comparison between metabarcoding and Lazaro are more balanced.

We added new information to justify the use of MCA to verify the results from metabarcoding and Lazaro, specifically by addressing the possibility that MCA was giving false negative results. The calculated LODs (limit of detection) for MCA were extremely low for all but three species examined by MCA, and therefore the likelihood of a false negative for these species was remote. The three species with higher LODs still had at least one or four positive MCA detections for two of these species, so MCA was still quite sensitive for these two species. The other species with a higher LOD had only four negative determinations (2% of total negative determinations), so its impact is minor. In addition, we added a statistical test to evaluate if some samples had a false negative MCA because the prey DNA was present but so scarce that there was no MCA template DNA in the sample. This test indicated that this circumstance was not observed. Thus, we have addressed another of the more critical comments of the previous reviewers.

Several controls were already in the original ms, but were probably difficult to notice, so we altered the text to highlight these better. These included DNA extraction methods, MCA negative controls (no template controls), and the availability of the bioinformatics scripts. We added details on the bioinformatics methods used to clean up the metabarcoding and Lazaro data. We have responded to specific reviewer issues below.

Finally, we added new analyses to investigate the possibility that the large number of individuals in a sample resulted in false negatives, and to explore the relationship between read number in true positive detections by metabarcoding and Lazaro with the relative template concentration determined by qPCR. We found no evidence that the large number of individuals in a sample resulted in a higher number of false negatives. In addition, we found that read number was consistently positively correlated with template concentration for Lazaro, but not for metabarcoding and the probability of a true positive was positively correlated with read number for Lazaro, but not for metabarcoding.

As can be seen from the resubmission, we made profound changes to the main text and hope our responses (indicated below after “>>>Response”) and the new text (including the supporting information) will be found satisfactory.

Please let us know if you have any questions.

Cheers,

Debora

Dear Dr Paula,

Thank you for considering GigaScience. Peer review of your manuscript is now complete and, in the light of the reports, and my own assessment as Editor, I regret to inform you that your manuscript cannot be accepted for publication in GigaScience.

Please find the reviewers' reports at the end of this email.

The reviewers agree that the project has merit and the manuscript contains interesting aspects. However, I am sorry to say that the reviewers also agree that there are major methodological issues, such as missing controls and quality checks. Overall, I feel that the submission was maybe a bit premature and a substantial amount of additional work, both in terms of experiments and write-up, would be needed to reach the high methodological standards we expect at GigaScience.

On a positive note, if at some stage you are able to fully address these concerns, also including additional experimental validation and controls, we may be able to consider a re-submission. If you resubmit to GigaScience at some stage, a cover letter explaining the revisions made should accompany the manuscript.

I wish you every success with your research and hope that you will consider us again in the future.

Best wishes,

Hans Zauner
GigaScience
www.gigasciencejournal.com

Reviewer reports:

Reviewer #1: This manuscript compares the output of metabarcoding and direct DNA shotgun sequencing for the dietary analysis of arthropod predators. The authors discuss the two techniques critically with reference to an example dataset and qPCR. The introduction summarises the various methods in use for characterisation of gut content DNA and explores the topics surrounding the manuscript aptly, but references to the literature are fairly scarce in a few places. The different methods compared make for an interesting comparison of emerging and existing methods, but the descriptions are lacking several key details and could benefit from some restructuring. I would particularly like to see more about the controls (positives, extraction and PCR negative and any blanks) and any post-bioinformatic data clean-up applied, all of which have important implications for the ultimate comparison. The discussion offers some critical insight into both techniques, but could be expanded in a few areas to include details relevant to a wide range of practitioners. The written English is sometimes in need of clarity, but these instances are addressed in the specific comments.
>>>Response: a) We added several references throughout. b) As already mentioned, we now added a control mock prey community, which provided extraction DNA controls, and known positive and negative and blanks (starved predator) controls for both metabarcoding and Lazaro. Regarding controls for the MCA, controls include true positive samples for all (n=32) but 7 species. One of these species was an aquatic oligochaete and, therefore, could not be a true positive species, and had 13 false positives and no true positives. The other 6 species had 12 detections total, of which 1 was considered a true positive based on the consistent melting curve for all technical replicates and no amplification of the NTC (no template control), 2 were considered false positives (no amplification of the NTCs), and 9 were not tested by MCA. There was a true positive and a NTC conducted for each of the other 25 species with a total of 232 detections. c) We are uncertain what the reviewer means by “post-bioinformatic” cleanup, as all of the cleanup is part of the bioinformatics workflow, but we provided considerably more details about the bioinformatics cleanup of the sequencing data in the main text and the supporting information. d) We have expanded and simplified the discussion to make it more relevant to a broader range of practitioners.

One aspect I found lacking is discussion around the prevalence of predator DNA in dietary analyses of arthropod gut contents. This is a substantial problem for many researchers in this field and the differential ability of the two compared methods to account for this would make an extremely valuable discussion (for example, the average % reads comprising predator for both).
>>>Response: This is a good point, and is especially a concern with metabarcoding, where the very high abundance of predator DNA can outcompete rarer prey DNA for primers. This has not been recorded as a problem with Lazaro, except that it requires sufficient sequencing depth. We added text about this to the metabarcoding discussion and mentioned it in the introduction.

The large number of individuals per sample could be better justified, as the read depth per individual is presumably miniscule by comparison to the whole sample, resulting in rare prey taxa being entirely eliminated in some workflows; indeed, this may be the cause of some of the inconsistencies between methods.
>>>Response: This is a very interesting and valid point, and stimulated us to reexamine our data. We have added data and analysis relevant to this point in a new

Table 4 and S6 and associated text. Read depth per individual is not that low for metabarcoding, where our read depth for the P. flavens libraries with 200 individuals per sample was about 15,000 reads per individual, compared to 10,000 reads per individual reaction recommended by Taberlet et al. (Taberlet, P., Bonin, A., Zinger, L. and Coissac, E., 2018. Environmental DNA: For biodiversity research and monitoring. Oxford University Press). It may be low for Lazaro (28,000 reads per individual for the same samples). This would suggest that there should be more false negatives (FN) for Lazaro samples than for metabarcoding samples, predicting that the false discovery rate (FDR, FP/(FP+TP)) should be higher for Lazaro than metabarcoding, and FDR should be negatively correlated with read depth per individual and/or read depth of detected prey across species. Our analysis in Table 4 and S6 shows no such relationships. Moreover, as the ant species we studied typically recruit massive numbers to prey, it is unlikely that rare prey will be associated with a single individual predator. Thus, while it is still possible that metabarcoding and Lazaro missed species that were rare, our data do not support this interpretation. We have added this information to the ms.

The reliance on consistency between the three methods used to confirm the results is arguably flawed, with each having individual problems and pitfalls (sometimes shared between two methods, e.g. PCR-based amplification resulting in disproportionate representation following bias). This is especially problematic given the lack of described controls, which could assist in identifying false positives.
>>>Response: We agree that each of the methods has its strengths and weaknesses, but the NGS methods are newer and all of the weaknesses are not known, while MCA and qPCR have been around a lot longer with known weaknesses. Our implementation of MCA should eliminate the possibility of a false positive, as it is based on the known melt curve of a true positive and confirmed non-amplification in a NTC for 93.2% of the initial determinations. MCA still has the potential to return a false negative (no detection of a rare DNA template, or the absence of the template when prey DNA is present but scarce). We addressed the first possibility by reporting very low LODs for detection by the Cq method, which is itself not as sensitive as the MCA method. This makes it unlikely that MCA missed detect rare templates. We addressed the second possibility by comparing read numbers of true and false positive determinations of a species. If the prey DNA was scarce, but detectable by metabarcoding or Lazaro, and there was no qPCR template, then the number of reads in false positives should be less than in true positives. For the species with sufficient observations to be statistically tested, this was true for only one of 7 comparisons, so we consider this to be unlikely as well.
It is possible that MCA for the species with the highest LODs might return a false negative. For *Cardiocondyla obscurior*, which had the highest LOD, this was unlikely. There was 1 detection by metabarcoding and Lazaro (verified by MCA), 2 detections by MCA only (not detected by either metabarcoding or Lazaro), and 3 non-verifications by MCA. These results suggest that MCA was more sensitive than either metabarcoding or Lazaro for this species. We did not report species-specific details in the resubmission because we did not want to bog down the manuscript with these details, but we are willing to add this information to the supporting information upon request.

While the manuscript is methods-focused and targeted at a molecular audience, the abstract should at least mention the species investigated (even if just coarsely listing the orders).
>>>Response: We added the information about the orders of the species investigated in the abstract.

Specific comments are outlined below:

Lines 22-23: The "27 samples, n=46 to 237 individuals/sample) is confusingly phrased. Simply stating "27 samples, 46-237 individuals per sample" would increase clarity.
>>>Response: Modified accordingly.

Line 37: The phrasing is a bit unclear here. Consider: "As DDSS does not currently allow sample multiplexing in a library…".
>>>Response: Modified accordingly.

Line 45: Some example references would be nice to support this statement, possibly a couple of large reviews of eDNA metabarcoding (e.g. by Taberlet et al.) or dietary metabarcoding (e.g. Clare et al.).
>>>Response: Modified accordingly.

Line 47: It would usually be common practice to put the acronym in parentheses, but if already in parentheses to separate with a semi-colon.
>>>Response: Modified accordingly.

Line 57: "Primer pairs" does not need to be hyphenated.
>>>Response: Modified accordingly where indicated and also throughout the text.

Line 58: Would "marker" or "gene region" be more appropriate than "barcode" in this instance?
>>>Response: We thought about it, but we don't think that "marker" or "gene region" would be a more appropriate terminology than "barcode".

Lines 62-72: Some of these statements could benefit from reference to the literature, particularly regarding the techniques discussed and their shortcomings.
>>>Response: The references were provided in the topic sentence at the head of the paragraph where the techniques were mentioned for the first time in the paragraph. As the subsequent sentences in the same paragraph are the development of the topic sentence and rely on the same references, we didn't see justification to repeat the citations in that context. In the case of Lazaro, the references were brought to the sentence head, instead of having them in the second sentence of the paragraph.

Line 65: "However" does not seem appropriate here and could be removed altogether.
>>>Response: We made modifications in the text to fit "however" better.

Line 66: "satisfactory assembly of prey mitochondria with…"
>>>Response: Modified accordingly.

Line 69: The "(s)" seems redundant.
>>>Response: The "(s)" was removed.

Line 70: Are there any studies that you could cite which have explored or discussed these effects?
>>>Response: Not that we are aware of.

Line 75: Quantification is mentioned, but I think it should be caveated. While many

would argue that DDSS is a more quantitative method (without PCR bias) it is still arguably heavily constrained by variation in biomass per individual consumed (further constrained by variation between life stages, sexes, etc.) resulting in, at best, biomass estimates. Even then, tissue DNA density and differential degradation of prey tissues in the gut are problematic, and to obtain even that, correction factors must first be devised in order to obtain any level of accuracy. This results in a semi-quantitative method at best. Admittedly, it is a contentious topic with many disagreeing parties, but some recognition of the work required for and the caveats imposed by the quantification of these data would be valuable here.
>>>Response: We made a modification in the text to soften the statement about quantification. We also developed some new analyses of our data to show that Lazaro read number is consistently correlated with relative prey template quantity, while metabarcoding read number is not.

Line 81: The first "or" should be replaced with a comma.
>>>Response: Modified accordingly.

Lines 82-84: The statement that these sequences "need to be elucidated" is very vague. Are you suggesting researchers should carry out this sequencing prior to DDSS gut content analysis (i.e. create bespoke reference libraries)?
>>>Response: Yes, we are suggesting that and this is true for any other method for gut content analysis based on DNA similarity (e.g., metabarcoding). Without the potential target prey species being present in the reference database, prey detection is fated to fail.

Lines 85-86: I would recommend rephrasing this to "there is no unanimous consensus on a "best practice" method…" to reflect that some groups would consider a given method best practice.
>>>Response: Modified accordingly.

Line 94: The lack of PCR bias seems an odd rationale to lead the hypothesis that DDSS would detect prey at a finer taxonomic resolution. Do you perhaps mean that the larger amount of data generated by DDSS (i.e. greater coverage) would increase the resolution? If not, a brief explanation could be beneficial for this hypothesis.
>>>Response: No, not exactly. We were referring to the larger target sequence for Lazaro. We modified the text to express that instead of mentioning “no PCR bias”.

Line 103: Pitfall trapping is an interesting choice for this study as some researchers worry that DNA in solution with the trapping fluid can enter the guts of the captured animals and misrepresent the dietary data. Some discussion around this would be valuable for clarity and transparency. It is not damning, but could be worth considering in the context of the two methods being compared.
>>>Response: It is true that live specimens placed in alcohol have a vomit response, and can lose prey DNA from the foregut into the alcohol. Prey, specimen and symbiont DNA can be found in the alcohol in which samples were collected (Linard et al. 2015). It is speculative if these prey will acquire DNA from the alcohol into their guts. The response of insects trapped in water has not been clearly demonstrated. In any event, we had taken care to remove any DNA adhering to the specimens based on Greenstone et al. (2012) before they were dissected, to avoid including DNA that was in the collecting medium, and described these methods in the revised ms. In addition,

as already mentioned, the guts were dissected as opposed to macerate the whole predators' body to and no external part of the predators were used.

Lines 103-107: Why these species? Some rationale would be valuable. Were they just the most common species? Presumably not given the presence of just one sample for many of them. Were they selected due to their similarity or even disparity in size/morphology? Some description would be valuable. Are the numbers per sample simply all that were present, or were attempts made to input the recommended tissue mass for the Qiagen protocol?
>>>Response: Those species were the most abundant predator species sampled. The numbers comprised all the specimens of the predator species present, except for *Ph. flavens* and *D. brunneus*, where the number of specimens was kept constant for the 12 samples of each species. The rationale was provided in the ms text now.

Lines 103-107: References are made to the coarse groupings of these species elsewhere, so it would be appropriate to mention the orders of these animals at this first mention (e.g. "Three ant (Hymenoptera: Formicidae) species were selected for analysis, from which we obtained 12 samples for each of the two more abundant species, Pheidole flavens (n=200 specimens/sample) and Dorymyrmex brunneus (n=100 specimens/sample), and one sample of Solenopsis substituta (n=273). One earwig (Dermaptera) sample was also analysed, of Euborellia annulipes (n=46), and one tiger beetle (Coleoptera: Carabidae) sample, of Tetracha sp. (n=49)", or something along these lines to coarsely designate the species).
>>>Response: Modified accordingly.

Lines 109-113: The references to sterilisation could benefit from clarification; was this bleach sterilisation? Or something else?
>>>Response: The sterilization method is described in the ms text now. It included bleach and autoclaving.

Lines 121-122: It would be valuable to know the amplicon length given the context of comparing two methods in this study; the amplicon length would have important implications for the amplification of degraded DNA and the taxonomic resolution.
>>>Response: The amplicon length generated by the Ins16S_1short primer pair is now provided. We also now provide their nucleotide sequences. The lengths of the MCA amplicons were already informed in Table S3 in the Supporting Information 2.

Lines 122-125: What reference databases are you referring to here? Most public databases would surely have far more data for COI than for 16S. Does the smaller 16S database not bias the results of this study against metabarcoding versus comparison against larger public databases? Some more information and context around the reference databases would be valuable.
>>>Response: Not sure what more the reviewer wanted here. The text already provided the context for the use of the 16S barcode instead of COI: 1) although having a smaller number of sequences, the 16S database had higher taxonomic coverage than the COI database; 2) bias of COI to amplify more lepidopterans and dipterans and failure to amplify other insect orders, such as hymenopterans; and 3) parts of 16S are more conserved across taxonomic groups, favoring a more 'universal' primer pair across insect orders). In the text it is stated clearly that COI is a larger database in terms of the number of sequences than 16S, but had lower taxonomic coverage. One major factor for this is because there are a lot of redundancies of COI depositions for

different vouchers of the same species. Consequently, the larger COI database size is not directly related to greater representation of prey species than 16S. Because we considered that the reviewer's concern is already addressed in the original version of the ms text, we did not implement any modification.

Lines 128-130: This implies that the entire gene is more conserved, which would mean taxonomic resolution would also be lower, unless the amplicon length was much greater. This should be rephrased to state instead that 16S has better conserved sites appropriate for universal primers with sufficiently variable amplicons for taxonomic resolution (if this is indeed the case).
>>>Response. Thank you. This is indeed what Sousa et al. showed. The suggested modification was made in the text.

Line 135: "Cycling conditions were: initial heat activation for 15 min at 95.."
>>>Response: Modified accordingly.

Line 135: Importantly, 40 cycles is more than recommended for the kit used and would increase bias. Was there a reason for 40?
>>>Response: The kit used was the Qiagen Multiplex PCR kit, in which according to the manufacturer Quick-Start Protocol from March 2016, it is recommended 30 to 45 number of cycles for standard multiplex PCR reaction and 25 to 40 number of cycles for amplification of microsatellites or short amplicons (up to 0.5 kb) using multiplex PCR. The 16S amplicon is around 190 bp. Therefore, 40 cycles is not more than recommended and we used 40 cycles to improve the likelihood of amplifying rare DNA of prey (or prey consumed a long time ago or in a small amount).

Lines 136-146: Was the same sequencing platform used for all data for consistency's sake?
>>>Response: Yes, the same sequencing platform was used for metabarcoding and Lazaro samples and this information was already provided in the original ms text. It is also true with the additional mock community study.

Line 149: Are these the same references databases mentioned above? If so, it would have been valuable to state this database compilation (or at least the source) alongside the details regarding database contents.
>>>Response: Yes, the metabarcoding database referred to in line 149 is the same referred above. As suggested, the source of the database is now informed alongside with the details of the database contents.

Lines 154-156: Why not use GenBank for the 16S genes too? It seems inconsistent and could benefit from some justification.
>>>Response: The European database (EMBL Nucleotide Sequence) is shared with GenBank (from USA) and DDBJ (from Japan) database (Stoesser et al. 1998, https://doi.org/10.1093/nar/26.1.8) so as sequences deposited in GenBank or DDBJ are shared daily with EMBL and vice-versa. So, ultimately, the 16S sequences from GenBank and DDBJ were already in EMBL and no such inconsistency exists.

Lines 156-159: How were these prey mitochondria generated?
>>>Response: The citation of the mitochondrial elucidation method is now provided.

Lines 219-228: Some citations for the equations could be valuable. Have they all been applied in similar contexts before?
>>>Response: Yes, they were. Citations were added in the ms text now.

Lines 204-218: Was there any post-bioinformatic data clean up carried out for the metabarcoding data? False positives are a consistent issue in metabarcoding and strict data clean up with reference to negatives, blanks and positives is one of the only effective means to have confidence in the data. Arguably, this is not possible for DDSS thus removing some degree of standardisation, but also by not doing it, this arguably underrepresents the stringency of the metabarcoding process.
>>>Response: For metabarcoding data, the cleanup of false positives is an embedded step in the OBITools package. So, no post-bioinformatic cleanup was performed for the metabarcoding data. The strict OBITools data cleanup using negatives, blanks and positives mentioned for metabarcoding can also be applied in Lazaro as is presented now in the 'Feeding bioassay controls' topic of the Methods section. So, the same rigor used for metabarcoding data cleanup was also applied for Lazaro data. Text was added now in the ms so that the degree of standardization between metabarcoding and Lazaro comparison is described.

Line 254: A redundant "or" (i.e. should read "of each prey species").
>>>Response: It was a redundant "of". Modified accordingly. Thank you.

Lines 263-265: It strikes me that the main prey orders are identical to those of the predators. Were these the same species? Could this simply be an artefact of misassignment of predator reads to other samples? How was this accounted for if not through post-bioinformatic data clean-up?
>>>Response: In some cases, predators were recorded as consuming other predators, but clearly, the methods cannot determine cannibalism. These determinations were at the species level and not at the ordinal or other higher than species taxonomic level. This information is described in Table 1, as was originally stated in the ms text. The majority of prey species detected were not the same as one of the predators, but the cases of a predator consuming another predator are cases of intraguild predation, which is common among generalist predators, particularly when they share habitat and are fairly abundant (both were the case). These identifications are not artefacts. MCA confirmed the DNA presence of the (predator) prey in the original DNA samples from the predator guts (or gasters), so they cannot be artefacts, misassignment of predator reads or any mistake of the bioinformatic processing of the sequencing data. This also demonstrates the significance of using MCA to verify detections without requiring any post-bioinformatic cleanup. The results from the control mock community experiment also corroborate the validity of intraguild predation. The same bioinformatics analyses were performed on these data, yet only prey and no predator species or their closely related species were identified in the experimental results from the control mock community. If the prey detection from the same orders as the predator could be attributed to misassignment or poor data cleanup, the species closely related to the predator would also have been identified in the controlled experiments results.

Line 266: Why not give the raw read numbers rather than ln? Single reads (singletons) could easily be a result of artefacts in the sequencing process; how have you ensured these are not erroneous?

>>>Response: The raw read number was provided already in Table S4 in Supporting Information 2. The ln numbers were used in the statistical analysis, so when reporting these results, it is most appropriate to report on the same scale. The ln scale is more appropriate than the untransformed scale for the metabarcoding reads, as they are based on exponential amplification. Because of this, the Lazaro reads must also be similarly transformed. Singleton reads were removed in both bioinformatics workflows, so they cannot be artefacts or erroneous determinations. These details were added to the descriptions of the workflows.

Lines 270-272: Please give the full genus name at first mention.
>>>Response: *D. brunneus* and *P. flavens* were cited with the full genera at the first mention in Material and Methods, and *P. tristis* was cited with the full genus in Table 1, i.e. they were all cited with the full genus previously to lines 270-272.

Line 276: "seems not unusual" could be clearer (e.g. "is not unexpected").
>>>Response: Changed to “may not be unusual”.

Lines 279-282: Standardisation would be good, but you have outlined the flaws in metabarcoding here, so presumably you would suggest standardising to DDSS? This is highly problematic given the financial inaccessibility of this technique for many researchers, particularly in regions where funding is less readily available. Perhaps instead you could state that it is an important consideration when comparing the data between studies, at least until an accessible and superior method is identified and made readily available.
>>>Response: Modified accordingly.

Lines 299-301: What thresholds did you use for taxonomic assignment? Surely this would at least partly mitigate this issue?
>>>Response: The Lazaro threshold determined for taxonomic assignment was 100% identity in at least an overlap length of 130 bp (150 bp reads). Now this is clearly informed in the Material and Methods section. Yes, the determination and use of a stringent threshold as this was essential to eliminate a lot of false positives as presented in Tables 1 and S4 (Supporting Information 2). We now also clearly provided the threshold used for the metabarcoding taxonomic assignment.

Lines 307-308: Indeed, this kind of contamination can be accounted for with effective controls and blanks. Importantly, the contaminant may only be present in the metabarcoding or DDSS data. Did you use controls and, if so, did you use these to limit these issues?
>>>Response: Actually, the kind of contamination we are referring to at this point in the manuscript is contamination that occurs after DNA extraction and there are no obvious controls that could account for these sources of contamination. Blanks are useful controls for determining if there is contamination during extraction. Our mock community work indicates that there was no contamination during extraction and suggests that contamination after extractions was not extensive. In addition, if there had been contamination during extractions, all three methods, Lazaro, metabarcoding and MCA would be expected to be positive, which was not the case.
Contamination of only the Lazaro or metabarcoding samples would occur after the sample was split into three parts. Lazaro sample contamination could occur during the plating or drying of the samples, and during the opening of the plate and library prep at

the genomics lab. There are no obvious controls for these sources of contamination, because they are highly unlikely to affect all samples equally and they are more likely to affect only a few samples. Therefore, any negative controls would be unlikely to be affected and thus they would give false confidence of the lack of these sources of contamination. Metabarcoding contamination could occur during sample preparation for amplification or handling the samples after amplification, as well as any of the possibilities for Lazaro contamination. Similar to Lazaro, there are no obvious controls for these sources of contamination, for the same reasons.

Lines 319-328: So, if it was detected by qPCR it was considered legitimate? What if the qPCR was contaminated? Were there sufficient controls in place?
>>>Response: The reviewer is concerned about the possibility of MCA false positives. MCA-qPCR was used as a final criterion to attribute a prey species detection because it is a very sensitive method and it checked for the presence of a potential prey directly in the source sample of DNA used for metabarcoding and Lazaro analysis. If qPCR became contaminated, the contamination would enter the reaction through the SYBR mix, water or primers. In any of these cases, the NTC (non-template controls) would have shown presence of contaminant, which it didn't. NTC is the qPCR reaction with all the components (SYBR, water and primers) except for the sample DNA. NTC were used in every plate for every primer pair and prey species tested. All this was already informed in the original ms text. Thus, the possibility of false positives was controlled. A possibility the reviewer did not consider is that MCA would give a false negative. We have now addressed this in detail with a description of the LOD and primer efficiency for each of the MCA primers, and a test for the possibility of false negatives.
There is no paper that has used MCA-qPCR before this study to check false detection (or lack of it) in environmental DNA detected by NGS based methods.

Lines 333-335: This seems indicative of a wider issue to me. Can you explain this, aside from either incorrectly identified false positives/negative, or a severe contamination issue? The numbers, to me, suggest the former; perhaps this was indeed present in L19, just simply not detected by the other methods for whatever reason.
>>>Response: The most likely explanation to the *Euschistus heros* detection by metabarcoding but lack of MCA-qPCR validation in sample 19 with detection of 262,723 reads is what it was already mentioned in the original ms text: taxonomic overclassification, i.e. insufficient taxonomic resolution between closely related species. The 262,723 reads in sample 19 only had 99% identity with *E. heros*, so it is possible that a related stink bug species (they were abundant in the sampled area as they are soybean pests) was indeed preyed upon by the predator of sample 19, but their 16S sequence was not in the reference database.

Lines 356-360: This seems like a major point of discussion, but is barely touched on. One of the greatest constraints of invertebrate gut content analysis is the prevalence of predator DNA in the output (even when dissecting out the guts). Some detail on the prevalence of predator DNA and a comparison of this problem between the methods deserves a much larger synthesis in this manuscript.

>>>Response: The discussion of this point (high prevalence of unassigned reads) was expanded in the ms text.

Line 362: Given that your conclusions are very much contextual, I don't think you are

prepared to answer this question outright, so it seems misplaced to ask it. Why not title this section something like: "Comparing metabarcoding and DDSS" or even, like the title, "Metabarcoding versus DDSS"?
>>>Response: Modified accordingly.

Line 367: "genus" should be "genera"
>>>Response: Modified accordingly.

Lines 367-369: You surely don't need both "hypothesis 1" and "(H1)". In the original description of the hypotheses, no numerical denominator is given, so perhaps select one and apply it there too.
>>>Response: Modified accordingly.

Line 370: Why "initially"? This didn't change over time, just the outcomes of separate comparisons differed.
>>>Response: Modified accordingly.

Line 401: This needs rephrasing as it doesn't currently make sense.
>>>Response: The sentence was rephrased.

Line 417: "undeniably"
>>>Response: Modified accordingly.

Lines 419-420: The detection of intraguild predation is dependent on the primer selection for metabarcoding, which could be further refined (especially through use of multiple markers). Whilst this would increase cost, the ability to include all such metabarcoding reactions in one sequencing run ultimately discounts this cost increase versus the full cost of DDSS for multiple samples.
>>>Response: Once we supplemented the metabarcoding reference database with the 16S sequences that we extracted from the mitogenomes we had sequenced, metabarcoding analysis detected considerable intraguild predation (IGP). Thus the lack of detection of IGP in the original manuscript was due to the incomplete reference database, and not the reliance on only one barcode.

Line 421: I have mentioned the quantitative issue above; this should be caveated here.
>>>Response: The mention of quantitative was removed from the ms text except where we have now shown a quantitative relationship between the number of reads detected by Lazaro and the relative template concentration determined by qPCR. In addition, we showed a quantitative relationship between the probability of detecting a true positive and the number of reads detected by Lazaro.

Line 426: Why 95? A 96 well plate can obviously contain 96 reactions, but even if controls are accounted for, "best practice" for controls would dictate the use of more than just one (PCR and extraction negatives throughout the PCR plate, mock community positive controls and blanks).
>>>Response: The mention of “95” was a misspelling and we removed it from the ms text.

Reviewer #2: Paula et al. in their manuscript titled "Metabarcoding versus direct DNA

shotgun sequencing for identification of prey consumed by arthropod epigeal predators" set out to compare two commonly used competing methods for molecular inference of species associations. Where the scale of the study is not especially large, producing data for few dozen aggregated samples for a handful of species, the study question remains relevant for anyone planning a study in the rapidly growing field of molecular food web research.

The study is reported very thoroughly, and anyone wanting to replicate the study would encounter no problems due to missing information. As well anyone wanting to use the data would understand how the data was generated. The authors are committed to have their data open and accessible. Overall, the laboratory-work seems of high quality, using trusted techniques and high-quality reagents.

The strengths and weaknesses of the study are intertwined. I find the use of MCA-qPCR in the verification of the prey detections a nice idea, but in the end, much of the results and their interpretation depend on the MCA-qPCR, performance of which cannot be independently estimated. Here, spiking the samples with known mock communities or few mock species would have helped to resolve the reason for some of the unexpected findings.
>>>Response: It is possible that a spike-in with a few mock species in each sample would have helped to resolved the reasons for the unexpected findings associated with Lazaro and metabarcoding, but it would not have helped resolve any of the issues associated with the major reasons we have proposed for the findings: taxonomic overdetermination, contamination after extraction, inability to resolve closely related species, lack of sufficient coverage of genetic variation in a reference species, or unknown flaws in the bioinformatics pipelines. We have provided considerably more detail about our use of MCA that should allow readers to evaluate independently our use of MCA to verify the detections by metabarcoding and Lazaro.

I find it to some degree concerning that the true and false positive prey detections showed no clear difference in read numbers and that the overlap between the prey detections of the three methods was so low. While I appreciate the forthcoming discussion of possible reasons for the findings, I can't help to wonder if some error may have happened either in the dual indexing for subsequent bioinformatic assignation of sample identity. Is this something that can be ruled out? The other option is, as was mentioned, gaps in the reference database and inaccurate species assignments. Here I note that details of the species assignment were the only poorly documented part of the manuscript, referring the reader to previous work. A simple thing to check would be if the false positive identification are poorer matches to the reference sequences than the true positives.
>>>Response: 1) Read numbers in true and false positive species. We were also concerned that there was no statistical difference in the number of reads for MCA-TP and MCA-FP, so we looked into this in three different ways and added these analyses to the resubmission. (a) If there is little amount of prey DNA left in the gut, it is possible that the prey sequence targeted by MCA is absent, while other sequences are detected by metabarcoding and or Lazaro. If this were occurring, then for a given prey there should be a lower read count in both metabarcoding and Lazaro when the MCA is negative than when it is positive. We tested this possibility for 4 and 5 species for metabarcoding and Lazaro respectively and only one species for Lazaro showed this relationship. Thus, this possibility was not supported. (b) If the number of reads was

positively correlated with the amount of prey DNA in the sample, then the probability of a true positive should be correlated with the number of reads across all species. We tested this with logistic regression, and found a significant positive relation for Lazaro, but not for metabarcoding. (c) If the number of reads was positively correlated with the amount of prey DNA in the sample, and for true positives within a prey species, the number of reads should be positively correlated with the relative template concentration determined by qPCR. This can only be tested for individual prey because amplification efficiency, baseline, thresholds are prey species specific in the qPCR for determining relative template concentration. We found a positive correlation for the Lazaro samples, and no correlation in most of the metabarcoding samples. We added methods, results and text in the resubmission on all three of these findings. 2) Dual indexing. We used unique dual indexing, as recommended for multiplexing libraries. This mitigates index hopping by filtering hopped reads in the initial assignment of reads to samples. While it is possible that the genomics lab made mistakes during the dual indexing, this would have resulted in substantial numbers of reads either being filtered before sample assignment or being misassigned to samples. We did not see evidence of either. Thus, misassignment is highly unlikely. 3) Species assignments. We have expanded the methods to provide details of the species assignment methods in both the main text and the Supporting Info. We found that supplementation of the metabarcoding reference database resulted in significant changes in the results. Thus, database gaps were very important as we had stated in the original manuscript. 4) Match of false versus true positives. The Lazaro threshold was 100% identity in at least 130 bp overlap length, which means that reads can have no mismatches with the reference. So the match of true and false positives cannot be different (always perfect matches). For the metabarcoding reads, the threshold was 98% identity in the ~190 bp amplicon, which allows at most 3 mismatches with the reference. With this threshold, a statistical analysis is not possible, but we explored the tendency in the data for the 3 species where this was possible. There was a slight tendency for the false positives to have a higher identity to the reference genome than the true positives (opposite expectation), but this was at most 0.06% with an SD of 0.3%. We mention this in the ms, but there we indicate that there was no tendency observed.

The introduction and the description of methods is very pleasing to read. The results and their subsequent discussion section, on the other hand, is quite heavy to read with many very specific species and sample - level examples. Some revising should be done to improve clarity and generality of the results and discussions section. Especially the first part titled "prey detections" is heavy on these details.
>>>Response: We have modified these sections to remove most of the detail and make the presentation more general.

Figures could be more usefull, showing all of the prey species and a figure summarizing the main metrics of performance: sensitivity, specificity , false discovery rate, false omission rate and accuracy would be preferable to table 4.
>>>Response: With the new results in the resubmission, which show no significant differences between metabarcoding and Lazaro for any of these measures, we believe a figure is not essential.

Finally, I have a few observations on a line-by-line basis (line numbers following the word document).

Line 89: When initially presenting these hypotheses here. It would be helpful to

introduce the hypothesis numbers used at the end of the manuscript and use them consistently throughout the manuscript.
>>>Response: Modified accordingly.

Line 93: For this hypothesis it is unclear what "taxonomic resolution" refers to. PCR bias does not affect taxonomic resolution, it itroduces a taxonomic skew into detection probabilities. The already mentioned poorer coverage of e.g. mitogenomic reference libraries will lead to more identifications with poorer taxonomic resolution, whereas DNA barcodes have been specifically chosen to separate species. Of course in a perfect world DDSS has the potential for a taxonomic level finer than species. Also this hypothesis doesn't match up with any of the three hypothesis discussed at the end of the manuscript, where it reads: "and hypothesis 3 (H3)= DDSS would be more prone to generate false negatives (less sensitive)." I would thus stick to the clear hypotheses discussed at the end of the manuscript.
>>>Response: We agree with the reviewer argument about the use of PCR bias to justify testing the hypothesis (H4) that Lazaro might give finer taxonomic resolution than metabarcoding. So that was removed from the ms text. However, we do not agree with the reviewer's suggestion of removing H4 from the ms because, as reviewer 1 pointed out, Lazaro presumably has the potential of providing greater coverage of prey DNA and simultaneous multiple independent queries to match with the sequences in the reference database. We kept H4 and we clearly indicated at the end of the ms discussion that H4 was rejected, i.e. both methods have similar taxonomic resolution.

Line 103: If the aim was to conduct a comparison of methodology, why use liquid pitfall trapped material, in which cross contamination between all sorts of taxa is inevitable?
>>>Response: This is a good question, but we believe we have minimized cross-contamination and eliminated the most obvious sources. We used pitfall traps with water and detergent to preserve the captured specimens, as recommended by Sutherland (1996; https://doi.org/10.1017/CBO9780511790508). This is particularly important when gut content analysis is intended. In addition, we limited the capture time to a 24 h period for similar reasons, which had the added advantage of reducing the number of specimens captured in a single trap, thereby reducing cross-contamination. It is well known that insects will regurgitate foregut contents when submerged in alcohol, and that these regurgitants will contaminate the exterior cuticle of co-occurring specimens. To mitigate this potential source of cross-contamination, we used the Greenstone et al. (2012; https://doi.org/10.1111/j.1755-0998.2012.03112.x) protocol of washing all the specimens with bleach and ultrapure water to remove external DNA contamination. In addition, the guts were dissected as opposed to whole body maceration, so contaminants that might remain after washing were avoided during sample preparation for DNA extraction. Now we provided this information in the ms text as other readers might have the same concerns.

Line 104: This part needs to give more information on the other species. Also what is the rationale behind this selection of 12 samples for the 12 samples for two common ants each, one for a less comon ant, one for an earwing and one for a carabid?
>>>Response: As this ms is on comparing the prey detection by gut content analysis using metabarcoding and Lazaro, the variable number of samples among species does not influence the results, as we are not comparing their food webs or trophic interactions. However, for the reviewer's information, the two common species were collected in abundance in all of the experimental treatments throughout the year, so the 12 samples correspond to different experimental treatments at different times of the

year. The collections of the less common species were pooled across treatments and seasons. The species used in this study were the most abundant predators collected.

Line 125: are the family and genera numbers for the CO1 database correct? Also which database are you referring to?
>>>Response: Yes, the family and genus numbers for the COI database used at the time are correct. We were referring to the COI of invertebrates obtained from EMBL. This information was already requested by reviewer 1 and added in the ms text.

Line 159: This is insufficient. We need to know how the species assignations were made as it may influence the downstream classification of as prey detections as true or false positive.
>>>Response: The species assignments of the specimens used as true positive controls were performed by morphological classification by the specialists listed in Table S2 (Supporting Information 2), and confirmed by MCA-qPCR analysis using species-specific primers (Table S3, Supporting Information 2).

Line 219: The key metrics analyzed here are given both short names (Sensitivity, specificity , false discovery rate and false omission rate) long names and their acronyms. The acronyms are used very little in the manuscript, and the long and short names used interchangeably is confusing. I suggest defining the terms here and then using just the short names in the manuscript.
>>>Response: We removed the acronyms, and are not sure what “long names” the reviewer is thinking about. We have standardized the ms to the “short names”.

Line 233: This sentence makes no sense, even if it is highly tautologous.
Also, "likelihood of false negative prey detection" is a very convoluted way to say "false omission" which is a somewhat convoluted way of saying "failing to detect prey items". This kind of double negatives occur also elsewhere in the manuscript causing some confusion.
>>>Response: The sentence was rephrased to improve clarity.

Line 270 and elsewhere: Referring to samples as libraries is somewhat confusing. It would be better to consistently refer to the samples as samples.
>>>Response: Modified accordingly.

Overall, the manuscript is an interesting piece of research with useful titbits of novel insight that will be directly useful for someone as myself, who has a few thousand pitfall trap samples waiting to be processed. I found the study to be sound in many aspects but would like to see a further investigation to the high occurrence of false positive detections as well as more clarity in the discussion part of the manuscript.

Best regards, Tuomas Kankaanpää,
Department of Agricultural sciences, University of Helsinki.
