# Supplementary material for: Metabarcoding versus mapping unassembled shotgun reads for identification of prey consumed by arthropod epigeal predators
Source: Gigascience. 2022 Mar 24;11:giac020. doi: 10.1093/gigascience/giac020 (PMC8952265; doi:10.1093/gigascience/giac020)
Supplement: giac020_GIGA-D-21-00303_Revision_1 [file giac020_giga-d-21-00303_revision_1.pdf]

GigaScience

Metabarcoding versus mapping unassembled shotgun reads for identification of prey consumed by arthropod epigeal predators

--Manuscript Draft--

**Manuscript Number:**	GIGA-D-21-00303R1
**Full Title:**	Metabarcoding versus mapping unassembled shotgun reads for identification of prey consumed by arthropod epigeal predators
**Article Type:**	Research
**Funding Information:**	Agricultural Research Service (USDA-NIFA 2016-67030-24950) — PhD David Andow
**Abstract:**	Background: A central challenge of DNA gut content analysis is to identify prey in a highly degraded DNA community. In this study, we evaluated prey detection using metabarcoding and a method of mapping unassembled shotgun reads (Lazaro). Results: In a control mock prey community, metabarcoding did not detect any prey, probably due to primer choice and or preferential predator DNA amplification, while Lazaro detected prey with accuracy 43-71%. Gut content analysis of field-collected arthropod epigeal predators (three ants, one dermapteran and one carabid) from agricultural habitats in Brazil (27 samples, 46-237 individuals per sample) revealed that 64% of the prey species detections by either method were not confirmed by Melting Curve Analysis and 87% of the true prey were detected in common. We hypothesized that Lazaro would detect fewer true and false positives and more false negatives prey with greater taxonomic resolution than metabarcoding, but found that the methods were similar in sensitivity, specificity, false discovery rate, false omission rate and accuracy. There was a positive correlation between the relative prey DNA concentration in the samples and the number of prey reads detected by Lazaro, while this was inconsistent for metabarcoding. Conclusions: Metabarcoding and Lazaro had similar, but partially complementary, detection of prey in arthropod predator guts. However, while Lazaro was almost 2× more expensive, the number of reads was related to the amount of prey DNA, suggesting that Lazaro may provide quantitative prey information while metabarcoding can not.
**Corresponding Author:**	debora Pires paula, PhD<br>Embrapa: Empresa Brasileira de Pesquisa Agropecuaria<br>Brasilia, DF BRAZIL
**Corresponding Author Secondary Information:**	
**Corresponding Author's Institution:**	Embrapa: Empresa Brasileira de Pesquisa Agropecuaria
**Corresponding Author's Secondary Institution:**	
**First Author:**	debora Pires paula, PhD
**First Author Secondary Information:**	
**Order of Authors:**	debora Pires paula, PhD
	Suellen Barros
	Rafael Pitta
	Marliton Rocha Barreto
	Roberto Togawa
	David Andow
**Order of Authors Secondary Information:**	
**Response to Reviewers:**	Dear Editor-in-Chief,<br>Thank you for considering our manuscript “Metabarcoding versus mapping unassembled shotgun reads for identification of prey consumed by arthropod epigeal

Powered by Editorial Manager® and ProduXion Manager® from Aries Systems Corporation

predators” (GIGA-D-21-00303) for publication in GigaScience. Please find below our point-by-point response to the reviewer comments. We agreed with their suggestions. We look forward to hearing from you and would be glad to respond to any further questions and comments.
Sincerely,
Debora
--

Reviewer reports:

Reviewer #1: Dear Dr. Paula
The manuscript has been improved considerably from the initial version. The introduction in now an enjoyable read with enough background on competing methodologies. The rest of the manuscript now offers sufficient detail to objectively assess the study and its results. The responses to reviewer queries were throughout and satisfactory. The supplementary materials are informative as well.
I still feel the methods comparison is somewhat unfair to metabarcoding, performance of which can be improved in several ways: e.g. processing individuals separately, while still sequencing them in a multiplexed single library; improving or adding primers; using blocking primers or optimising PCR conditions (40 cycles will definitely mask rare prey species due to the exponential nature of amplification). Most of these aspects are of course discussed, and attention is drawn to them by the a priori hypotheses not being confirmed.
>>>Response:
a) The individuals were processed equally for both methods as the total DNA extracted was split among the 3 molecular methods (metabarcoding, Lazaro and MCA-qPCR) to avoid any bias regarding sample processing. In fact, we suppressed the tagging step to avoid tag jumping source of errors (false positives), ultimately enabling metabarcoding to perform more accurately than usual.
b) The argument that using additional primers would improve detection of true positive prey could also be used to suggest an increased detection of false positive prey. Although there are a number of publications that demonstrate that the use of multiple primers increase the diversity of prey detection, they often don’t present a concomitant analysis of the effect on false prey detection. Moreover, there is no guarantee that the use of multiple primers will always improve metabarcoding performance. To illustrate this point we added additional illustrations in the Fig. S5 (Supporting information 1) to show the potential mismatches of the 16S barcode primer pair that we used and of 3 commonly used COI barcode primer pairs (LCO1490 and HCO2198, amplicon length ca. 710 bp, Folmer et al. 1994; mICOIintF and jgHCO2198, amplicon length ca. 313 bp, Leray et al. 2013; UniMiniBar, amplicon length 100-150 bp, Meusnier et al. 2008) with the sequences of the prey species used in the control mock community assay and detected in the field gut content samples. As it can be seen, the COI primer pairs LCO1490/HCO2198 and UniMiniBar had many more mismatches than our 16S primer pair, and therefore would likely have detected fewer prey species than the 16S primer pair. The COI primer pair mICOIintF and jgHCO2198 has high degeneracy, and could have performed equivalently or a little better than the 16S primer pair, but the high degenerance comes with the price of lower primer amplification efficiency. In addition, the forward primer (mICOIintF) seems to form a mismatch in the critical last three bases of the 3’-end with all 6 species used in the control mock community assay and in 8 out of 29 species (27%) detected in the field samples. Regarding research to improve a metabarcode primer pair, this has not been not easy, and several studies have been published on the search for better metabarcodes, but due to many factors (e.g.,taxonomic coverage; sample nature complexity) there have been no breakthroughs. So, we used one of the barcode primer pairs available nowadays to make the comparison between the two detection methods.
c) The use of blocking primers is not recommended for gut content analysis of generalist predators because the blocking primers block the detection of species closely related to the predator. In biodiversity surveys, it might be useful to use blocking primers, yet one could still face problems (Pinol et al. 2014, doi: 10.1111/1755-0998.12156). The PCR conditions used in this study were optimised previous to the sample analysis, including the number of cycles to be used.

Nevertheless, the article is helpful to anyone aiming to either further develop methods for molecular analysis of trophic interactions or to choose an existing protocol for

Powered by Editorial Manager® and ProduXion Manager® from Aries Systems Corporation

ecological studies. The melting curve analysis elevates the study to a level required by the journal.
Thus, I only have few very small comments (line numbers refer to the word document).

Line 84: I know this was suggested by reviewer 1. but a "unanimous consensus" is a tautology
>>>Response: Modified accordingly.

Line 98: should be: one adult aptera
>>>Response: Modified accordingly.

Line 338: should be: Neither metabarcoding or Lazaro produced any false positives
>>>Response: Modified accordingly.

Line 478: I would say higher detection of false positives instead of lower in this sentence to be consistent with the hypothesis and the previous two sentences. "this did not convert into a higher detection of true positive or a lower detection of false positive prey detections compared to Lazaro."
>>>Response: Modified accordingly.

The reference list needs some touches to match journal guidelines. E.g. doi format, italisation of journal names.
>>>Response: References were revised and meet the journal guidelines .

Yours, Dr. Tuomas Kankaanpää, Ecology and Genetics unit, University of Oulu.

Reviewer #2: The effort taken by authors to address the comments from the previous round of review is significant and admirable. The authors have clearly taken great care and effort to strengthen the manuscript, which has evidently led to some excellent additions. The experimental controls and more detail around the overall QC are very welcome additions. The additional information regarding the number of individuals per sample and sequencing depth are very valuable too. The differentiation in nomenclature between "DDSS" and "Lazaro" is a good idea (and the new name is great)!
Regarding the response to the comment about data cleanup, appreciably "post-bioinformatic" is quite vague and disregards any processes incorporated into the bioinformatics. The additional information and controls presented in the revised manuscript are an excellent addition. Many metabarcoding studies apply quality thresholds (sometimes arbitrary, e.g., remove taxa with <100 reads, sometimes more uniform and robust, e.g., remove any reads that comprise less than 1 % of the sample read count) to remove false positives that arise through contamination/error. Whilst the scale of these errors might be reduced in Lazaro (i.e., no PCR amplification of false positives), there are still many opportunities through which they may arise (e.g., environmental/lab contaminants, sequencing errors) so it would be interesting to know how these might best be tackled going forward. Given the novelty of these techniques, it is appreciably difficult to determine best practice, so it is great to see that this is now more overtly incorporated.

In the response to reviewers, it is mentioned that controls will not identify contamination that occurs after DNA extraction, but that is not always the case. A distinction between negative controls (e.g., extractions without input tissue or PCRs without input DNA) and blanks (e.g., unused combinations of used sample tags). Equally, running samples in triplicate (as is done for some aspects of this manuscript) can facilitate identification of post-extraction contamination. Of course, these are just suggestions for further applications and would be an unfair expectation of every such study given the financial upscaling associated. Whilst the authors are correct in suggesting that this type of contamination may occur infrequently and thus contamination may not be detected by these controls, the statement that this means the controls give false confidence in the lack of contamination should be revisited. Surely not screening for this contamination at all is much worse in this regard than checking and not discovering any contamination.
>>>Response: We agree that not checking at all for contamination is a bad idea, and the reviewer has made some suggestions that could be examined in the future. Our concern was with infrequent contamination of a few samples after DNA extraction. As

there are many steps between DNA extraction and sequencing, the best approach for detecting contamination is still not clear. The idea of using unused combinations of the sample tags as blanks would check if contaminants were entering via the tags or if many samples were being contaminated during the PCR step. The idea of maintaining the triplicate PCR products separate, would help detect contamination during the PCR step, however it would increase the likelihood of missing the rare DNA. Contamination could occur at many more steps of the process, so this problem merits serious investigation that goes beyond the scope of the present study (as recognized by the reviewer).

Regarding cannibalism, is there no potential to identify instances of cannibalism in future studies by looking for intraspecific genetic variation? Obviously this may be beyond the scope of this study, but it would certainly be a great benefit over metabarcoding if it could be achieved. Even if only by incorporation of phylogenetic distances between individual predators in the analysis, there seems to be great potential for lateral streams of data using this approach which appear to be otherwise untapped.
>>>Response: In another study, we are precisely testing this hypothesis if it is possible to distinguish different specimens by intraspecific genetic variation. But as the reviewer said, this is beyond the scope of this study.

Some specific comments:
Lines 19-20: Here it is stated that metabarcoding did not detect any prey and that Lazaro detected 43-71 %. Without stating the possible reasons for the poor detection of prey by metabarcoding, this seems to be an unfair representation, since it seems to come down to primer choice. Positive results from ecoPCR for amplification of the target species is mentioned later as a justification for it not being a primer problem, but importantly ecoPCR is a very simple simulation of PCR, neglecting critical aspects of primer success such as 3' proximity and adjacency of mismatches. Ultimately, it seems that primer mismatch or preferential amplification of predator DNA were likely causes of this issue, which is of course an indictment of metabarcoding more generally anyway (i.e., primer mismatches are unpredictable and can hamper taxonomic coverage sporadically), but it could obviously have been circumvented by alternative primer choice, or by using multiple primer pairs. Ultimately though, to test whether this primer mismatch issue is the cause of this issue, one could simply diagnostically test tissue extracts from these taxa with the primers in question.
>>>Response: We added a statement in line 20 to express that one possibility why metabarcoding did not detect any prey from the mock community could be related to our primer choice and or preferential predator DNA amplification. However, ecoPCR has a feature to avoid adjacent mismatches in the 3' proximity, as informed in the example 2 of the software documentation (https://pythonhosted.org/OBITools/scripts/ecoPCR.html): "a perfect match can be enforced by adding a '#' after the considered nucleotide. Example 2:
> ecoPCR -d mydatabase -e 2 -l 80 -L 120 -D 50 -r 7742 \
TTAGATACCCCACTATG#C# TAGAACAGGCTCCTCTA#G# >
mysequences.ecopcr". We used this # feature to avoid adjacent mismatches in the 3' proximity. Additionally, one should also consider that the use of multiple barcode primers can simultaneously increase the rate of false positive detection, so if the use of another primer could have resulted in true prey detection, it could also have resulted in increased false prey detection. In addition, we only used one reference database for Lazaro's analysis as well, when in fact we could have used multiple reference databases to improve prey detection by Lazaro. We added these to the main text.

Line 27: It is stated that Lazaro and metabarcoding are "similar in these respects" and then two percentages are given, but what do these refer to? Detection of true positives, false positives and false negatives, and taxonomic resolution are all mentioned, so it is not clear which these percentages pertain to.
>>>Response: Modified accordingly.

Lines 28: The statement that metabarcoding detected 350X more true prey reads is surely uninformative because those reads are amplified, so what does this tell us? It is then stated that this doesn't convert into a higher number of true positive prey detections, which is to be expected. It is probably a valuable statement for the

	manuscript, but it doesn't feel important enough for the abstract when there are so many great findings already there.<br>>>>Response: Modified accordingly.<br><br>Line 34: I feel that the "may" and "can" should be flipped. Whilst there is some debate around quantification of metabarcoding reads, there seems to be a general consensus that even the best attempts require additional experimental data (i.e., it cannot be quantitative in isolation). Lazaro, however, shows promise for quantification, but without more studies and data from alternative applications, this seems speculative still (thus "may" could be better).<br>>>>Response: Modified accordingly.<br><br>Line 57: "do not assemble"<br>>>>Response: Modified accordingly.<br><br>Line 57: "prior" may be more appropriate than "previously"<br>>>>Response: Modified accordingly.<br><br>Line 80: This is the first main text mention of Lazaro; it would be valuable to introduce it. It is introduced on line 88; perhaps these could be switched. It might be nice to include briefly the justification for the name too.<br>>>>Response: Modified accordingly.<br><br>Lines 96-103: Was it visually confirmed that all seven prey were consumed by the predators?<br>>>>Response: Yes, it was visually confirmed. Now we mention that in the main text.<br><br>Lines 286-307: It's unconventional to present hypotheses at the end of the methods. It would seem more logical to include them at the end of the introduction. This is not, however, to say that this should not be done unless the editorial team stipulate otherwise. It just might be worth considering if it makes logical sense to present the hypotheses before the methods (i.e., indicative of a hypothesis-led study) or as they are (i.e., indicative of an exploratory study).<br>>>>Response: We appreciate the suggestion, but we prefer to keep the hypotheses as they are.<br><br>Lines 432-435: Could it not be said that these differences between the methods support the idea of using both in tandem? The manuscript is very much focused on the competition between the techniques, but some exploration, however brief, of their compatibility might be interesting.<br>>>>Response: That's a good idea. Thank you. We added the idea of using both methods in tandem. We had already stated that the methods were complementary and used them together to construct the food web.<br><br>Line 457: redundant "are" before "may"<br>>>>Response: Modified accordingly.<br><br>Lines 472-473: Again, the mock community results seem somewhat problematic for this statement given the primer selection problem.<br>>>>Response: Answered in the first response to this reviewer, and also in the response regarding lines 19-20.<br><br>Line 498: Is this metagenomic work by Srivathsan et al. not methodologically different from Lazaro? My understanding is that Lazaro is a distinct method, hence the new name.<br>>>>Response: Reviewer is correct. Modified accordingly.
**Additional Information:**	
**Question**	**Response**
Are you submitting this manuscript to a special series or article collection?	No
**Experimental design and statistics**	Yes

Powered by Editorial Manager® and ProduXion Manager® from Aries Systems Corporation

Full details of the experimental design and statistical methods used should be given in the Methods section, as detailed in our Minimum Standards Reporting Checklist. Information essential to interpreting the data presented should be made available in the figure legends.<br><br>Have you included all the information requested in your manuscript?	
**Resources**<br><br>A description of all resources used, including antibodies, cell lines, animals and software tools, with enough information to allow them to be uniquely identified, should be included in the Methods section. Authors are strongly encouraged to cite Research Resource Identifiers (RRIDs) for antibodies, model organisms and tools, where possible.<br><br>Have you included the information requested as detailed in our Minimum Standards Reporting Checklist?	Yes
**Availability of data and materials**<br><br>All datasets and code on which the conclusions of the paper rely must be either included in your submission or deposited in publicly available repositories (where available and ethically appropriate), referencing such data using a unique identifier in the references and in the "Availability of Data and Materials" section of your manuscript.<br><br>Have you have met the above requirement as detailed in our Minimum Standards Reporting Checklist?	Yes

Powered by Editorial Manager® and ProduXion Manager® from Aries Systems Corporation

Manuscript

Click here to access/download;Manuscript;GIGA-D-21-00303R1-main text-Feb 8 revDPP.docx

Metabarcoding versus mapping unassembled shotgun reads for identification of prey consumed by arthropod epigeal predators

Débora Pires Paula[1,*], Suellen Karina Albertoni Barros[2], Rafael Major Pitta[3], Marliton Rocha Barreto[2], Roberto Coiti Togawa[1], David A. Andow[4]

[1] Embrapa Genetic Resources and Biotechnology, Brasília-DF, Brazil;

[2] Universidade Federal de Mato Grosso, Sinop-MT, Brasil;

[3] Embrapa Agrosilvopastoral, Sinop-MT, Brazil

[4] Department of Entomology, University of Minnesota, MN, USA.

*Corresponding author: debora.pires@embrapa.br

Phone: +55 (61) 34484929; Fax: +55 (61) 34484672

Débora P Paula [0000-0003-1199-5210];

Suellen Barros [0000-0002-8783-0570];

Rafael Pitta [0000-0002-8503-7106];

Marliton Barreto [0000-0003-3793-8855];

Roberto Togawa [0000-0001-7179-3210];

David Andow [0000-0002-7730-2686].

Abstract

Background: A central challenge of DNA gut content analysis is to identify prey in a highly degraded DNA community. In this study, we evaluated prey detection using metabarcoding and a method of mapping unassembled shotgun reads (Lazaro). **Results:** In a control mock prey community, metabarcoding did not detect any prey, probably due to primer choice and or preferential predator DNA amplification, while Lazaro detected prey with accuracy 43-71%. Gut content analysis of field-collected arthropod epigeal predators (three ants, one dermapteran and one carabid) from agricultural habitats in Brazil (27 samples, 46-237 individuals per sample) revealed that 64% of the prey species detections by either method were not confirmed by Melting Curve Analysis and 87% of the true prey were detected in common. We hypothesized that Lazaro would detect fewer true and false positives and more false negatives prey with greater taxonomic resolution than metabarcoding, but found that the methods were similar in sensitivity, specificity, false discovery rate, false omission rate and accuracy. There was a positive correlation between the relative prey DNA concentration in the samples and the number of prey reads detected by Lazaro, while this was inconsistent for metabarcoding. **Conclusions:** Metabarcoding and Lazaro had similar, but partially complementary, detection of prey in arthropod predator guts. However, while Lazaro was almost 2× more expensive, the number of reads was related to the amount of prey DNA, suggesting that Lazaro may provide quantitative prey information while metabarcoding can not.

Keywords: diet analysis; environmental DNA; generalist predators; gut content analysis.

Data Description

Background

The use of high throughput DNA sequencing (HTS) for studying species composition or diversity in environmental samples has been widely adopted and metabarcoding has become the most commonly used method to study environmental DNA (eDNA) [1-3]. In metabarcoding, target barcode regions are enriched through PCR and sequenced for taxonomic identification (specific taxa or operational taxonomic units; OTUs) through a bioinformatic workflow [4-8] by similarity of query sequences with taxonomically identified barcode sequences in a reference database [9].

The main limitations of metabarcoding are related to bias in primer amplification efficiency during the target barcode enrichment process and amplification errors [10-13]. Optimal primer pair(s) would amplify the barcode region(s) of a broad taxonomic range with equivalent efficiency across taxa, avoid formation of chimeras among closely related or abundant sequences, and provide the desired taxonomic resolution without missing any taxon [9,10,12,14]. Such a primer pair has yet to be found, so most recent metabarcoding studies design primers have focused on amplifying barcodes of specific taxonomic groups and/or employing multiple primer pairs for the same or different barcodes. In addition, for predator gut content analysis, the more common predator barcode sequences could mask amplification of closely related species.

Alternative methods for species identification that have no sample DNA enrichment have been developed, and include those that assemble or do not assemble the reads prior to mapping them to a reference database. Methods with no sample DNA enrichment in which reads are assembled include mitochondrial metagenomics [15-21], metagenome skimming [22], and enrichment of a barcode

sequence by hybridization capture followed by HTS [23]. Mitochondrial metagenomics and metagenome skimming are promising methods for general biodiversity surveys, but not for predator gut content analysis because they rely on assembling genomes (organelles or nuclear genetic material) to function as a 'superbarcode' to identify species. As the DNA community in predator guts is degraded by digestion, satisfactory assembly of prey mitochondria with sufficient coverage is difficult and, therefore, the application of mitochondrial metagenomics and metagenome skimming is compromised. Hybridization capture replaces PCR to enrich the barcode sequences in a sample, and is suitable for gut content analysis. However, as it still depends on an intermediate step of enrichment of a particular barcode, it might also be subject to bias related to probe design and fidelity/efficiency of the hybridization.

Assembly-free methods have been more recently proposed [24;25], but just a few were tested for gut content analysis [26-29]. Their principle basically comprises the direct eDNA sequencing and mapping the unassembled reads to a reference database for taxa identification using a threshold of high similarity (>95%) with a minimum predefined overlap length for the matches. No barcode primer pair or probe is required, hypothetically minimizing bias and favoring quantitative estimates of prey content. Without amplification, however, the detection of rare eDNA is likely reduced. A major limitation is that different samples cannot be multiplexed in a single or few libraries because there is no sample DNA enrichment step where individual tags are assigned to each sample. Consequently, every sample has to have its own library, increasing the total cost in library construction. In addition, while all methods based on DNA similarity require a reference database, mapping of unassembled shotgun read method (named Lazaro as it represents "resuscitation" of the prey DNA) requires mitogenomes, other organellar genomes or nuclear genome fragments in the reference database [29]. Therefore, suitable

sequences of candidate prey (*e.g.*, species co-occurring with the predators) are often missing from the database, so they need to be elucidated and added to the reference database, otherwise the prey cannot be detected.

Despite great advances and several options, at the moment, there is no consensus on a "best practice" method for eDNA study of gut contents [9]. To improve the applicability of large-scale DNA-based methods for prey detection, this work examined the sensitivity, specificity and accuracy of using metabarcoding and Lazaro (mapping of unassembled shotgun read method) to identify prey in the guts of several epigeal agricultural predators. We used Melt Curve Analysis (MCA) to verify detections by the two methods and examined the number of true and false prey species detected, the number of true and false non-detections recorded, the taxonomic resolution of true detections, and the relation between the number of reads for a detection and the relative prey DNA concentration.

Materials and Methods

Control mock prey community

Newly emerged (48 h) unfed harlequin ladybird *Harmonia axyridis* (Coleoptera: Coccinellidae) adults (n=10, sex ratio of 1:1) were individually supplied simultaneously with seven species of prey, which were visually confirmed to be consumed within one hour. These were one adult aptera of the aphids (Hemiptera: Aphididae) *Aphis glycines*, *A. gossypii*, *A. craccivora*, *Acyrthosiphon pisum* and *Myzus persicae*, and one egg of the diamondback moth *Plutella xylostella* (Lepidoptera: Plutellidae) and *Cycloneda munda* (Coleoptera: Coccinellidae). Immediately before feeding (negative control) and after feeding, five beetles per sex were placed in 95% ethanol and stored at -80°C.

Arthropod field sampling

Epigeal arthropod predators were sampled twice a month (Brazilian authorization SISBIO 33683-1) in 2014/2015 (July-September) in Sinop-MT/Brazil for a 24 h period [30] in pitfall-traps buried level with the soil in four replicated agricultural experimental plots: soybean/maize (*Glycines max*/*Zea mays*), palisade grass (*Brachiaria brizantha*), eucalyptus plantation (hybrid of *Eucalyptus grandis* and *E. urophylla*), and an additive mixture of all three. Pitfall-traps contained 750 ml of water and two drops of detergent to break surface tension and preserve the captured specimens [31]. We obtained 12 samples for each of the two more abundant ant species (Hymenoptera: Formicidae), *Pheidole flavens* (n=200 specimens/sample) and *Dorymyrmex brunneus* (n=100 specimens/sample), and one sample of *Solenopsis substituta* (n=273), one sample of the earwig *Euborellia annulipes* (Dermaptera: Anisolabididae) (n=46) and one sample of the tiger beetle *Tetracha* sp. (Coleoptera: Carabidae) (n=49). These species were the most abundant predator species sampled and all the specimens were used for DNA extraction.

DNA extractions

To clean external DNA from the specimens, all the specimens from the feeding bioassay controls and from the field, before DNA extraction, were soaked individually for 40 min in 2.5% commercial bleach in 1.5 microtubes, followed by orbital rotation at 2×*g* at 4°C for 40 min, discarding the washing solution and rinsing the specimens for 5× in ultrapure water [32]. For the ants, the gaster was separated and collected, and for the other species, guts were dissected under a microscope (30× magnification) immediately before DNA extraction

using sterilized entomological dissecting tools. Sterilization was performed by soaking the dissection tools in 0.5% sodium hypochlorite for 10 min and autoclavating (121°C at 1 atm for 20 min), followed by rising abundantly with ultrapure water (MilliQ) to minimize cross-contamination. Dissected guts or gasters from the same sample were pooled in a lysis buffer from the kit DNeasy Blood & Tissue (Qiagen), placed on ice and macerated with sterilized glass pestles separately for each sample. Cross-contamination was minimized by sanitizing surfaces and sterilizing all equipment and materials between specimen dissections, and filter tips were used to handle all liquids containing DNA. Total DNA extraction was performed using DNeasy Blood & Tissue (Qiagen) kit. DNA purity and concentration were assessed by the NanoDrop™ spectrophotometer. DNA quantity was normalized to 1 mg/ml across samples and split into three parts, one for Lazaro, one for metabarcoding, and one for MCA in qPCR.

Preparation of the DNA samples for metabarcoding and Lazaro analyses

For Lazaro, the pertinent aliquots obtained from the previous step were normalized to 150 ng DNA/sample. For metabarcoding, a region of the 16S mitochondrial gene was amplified using the primer pair Ins16S_1short (forward 5´-TRRGACGAGAAGACCCTATA-3´ and reverse 5´-ACGCTGTTATCCCTAAGGTA-3´), which generates an amplicon of around 190 bp [11], following the 'recommendation' of using primer pairs that generate amplicons <200 bp for more degraded environmental samples, as gut contents [33]. 16S barcode was chosen over COI for several reasons. Although the amount of arthropod 16S sequences in GenBank was considerably smaller (133,899 sequences, 2,630 families, 5,829 genera and 48,711 species, obtained from GenBank using the search *arthropod[organism] AND 16S*, release date to 2018/12/31) than the COI sequences (2,570,787 sequences from 1,395 families, 10,394 genera and 26,024 species;

obtained from GenBank using the search *coi[Gene Name] AND arthropoda[Organism] AND "1900"[Publication Date] : "2018/12/31"[Publication Date]*), it had higher taxonomic coverage. In addition, Clarke et al. [11] demonstrated better taxonomic coverage of 16S than COI and bias of COI to amplify more lepidopterans and dipterans, while failing to amplify other insect orders (e.g., hymenopterans). Lastly, according to Deagle et al. [10], Elbrecht et al. [34] and Sousa et al. [35], 16S has been preferentially used because 16S has some regions of more conserved sites across taxonomic groups, spanning sufficiently variable regions among taxa, resulting in more universal primers with equivalent taxonomic resolution than COI. Primers were not tagged to eliminate bias related to the tagging process [36], so an independent library was produced for each epigeal predator DNA gut sample. PCR reactions (0.2 µM primer pair) were performed in triplicate using Qiagen Multiplex PCR Master Mix and adding 1.28 µg/µl of bovine serum albumin (BSA) to prevent PCR inhibition [37]. Cycling conditions were: initial heat activation 15 min at 95°C, 40 cycles of 3-step cycling (denaturation 30 s at 94°C, annealing 90 s at 60°C, extension 90 s 72°C), and final extension for 10 min at 72°C. Triplicates were pooled and purified using QIAquick PCR Purification Kit (Qiagen). Amplicons were quantified by NanoDrop™ and normalized in equimolar ratios across all the metabarcoding samples. We opted for not multiplexing the 27 metabarcoding samples to keep the same coverage between metabarcoding and Lazaro methods.

DNA sequencing

All Lazaro and enriched barcode samples were dried in a speed vacuum centrifuge. For the feeding bioassay samples, 20 samples were dried, which comprised 5 treatments (without feeding and four times after feeding) ×2 sexes×2 methods (metabarcoding and Lazaro).

For the field samples, 54 samples were dried, which comprised 27 for metabarcoding and 27 for Lazaro. The dried feeding bioassay and field samples were shipped simultaneously to the Roy J. Carver Biotechnology Center (University of Illinois at Urbana-Champaign) to construct KAPA Hyper libraries (Kapa Biosystems) with insert size 200 to 600 bp using unique dual indexes. Quality checked samples were sequenced by Illumina HiSeq4000 (Illumina HiSeq 3000/HiSeq 4000 System, RRID:SCR_016386; 150 bp paired-end, 151 cycles, HiSeq 4000 sequencing kit version 1) in a single lane. The Brazilian license to access the genetic heritage was provided by CGEN/SISGEN A8E3D94. Sequence Read Archive (SRA) access codes are presented in Supporting Information 1.

Reference DNA databases and bioinformatic analysis

For metabarcoding, the reference database was constructed by extracting invertebrate 16S barcode regions from the European Nucleotide Sequence database (EMBL) (release 132; inv: invertebrate database/division; std: standard) using the ecoPCR program [38]. The EMBL is shared daily with GenBank (from USA) and DDBJ (from Japan) databases [39]. In addition, 16S sequences for several species that were collected in the pitfall traps were determined and added to the other arthropod16S sequences obtained from GenBank and, after *in silico* PCR using ecoPCR and the Ins16S_1short primer, resulting in a 16S amplicon database composed of 63,618 sequences for 39,397 species from 2,172 families. Prey detection analysis was performed using OBITools as in [26, 40,41]. The metabarcoding threshold for taxonomic assignment was 98% identity and reads with count lower than 100 were removed. Only ‘head’ and ‘singleton’ identifications were considered.

For the Lazaro reference database [28,29], we constructed a comprehensive arthropod mitochondrial DNA database by obtaining all sequences (partial or complete, Fasta format) available at the time at GenBank (n=3,381, distributed in 2,779 species from 1,850 genera in 598 families). In addition, following the mitochondrial elucidation method described in Paula et al. [28,29] and is briefly presented in Supporting Information 1, we provided mitochondrial sequences of 29 taxa (Table S1, Supporting Information 2) corresponding to the main potential prey co-occurring with the sampled epigeal predators in the experimental plots, including the predators under analysis (taxa and taxonomic determinations in Table S2, Supporting Information 2). For the taxonomic prey identification, we used the Lazaro method [42], which is designed to detect and quantify species from degraded eDNA samples. Briefly, this method takes raw BlastN (BLASTN, RRID:SCR_001598) output of hit matches, identifies the mismatches (or SNPs) between the query and reference sequence, removes false mismatches (e.g., degenerate IUPAC nucleotide codes, e.g., R=A or G; Y=T or C; S=C or G; etc), reanalyzes overlap length and percent identity, filters the best hit matches with an overlap-identity threshold, eliminates singleton reads, and filters the reads mapping to coding regions of their respective reference mitogenome. The scripts are available in the GitHub repository [43]. The best overlap-identity threshold was determined from previous experimental data [42] and determined to be at least 100% identity in an overlap length of at least 130 bp (Supporting Information 1). Fastq files were generated and demultiplexed with the bcl2fastq (bcl2fastq, RRID:SCR_015058) v2.17.1.14 Conversion Software (Illumina). The quality assessment for each dataset was done using FastQC (FastQC, RRID:SCR_014583) (v.0.11.3) [44]. Low quality sequences (Phred<30) and library index adaptors were trimmed by Fastqc-mcf (v.1.04.807) [45] and Cutadapt (cutadapt, RRID:SCR_011841) (v.1.9.1) [46]. Retained good quality Fastq reads were converted to Fasta format by SeqTK (Seqtk, RRID:SCR_018927) (v1.2) [47].

MCA confirmation of the field detected prey

For the field samples, we performed Melting Curve Analysis (MCA) in qPCR to check the presence of the prey DNA detected by metabarcoding and Lazaro. The principle is based on the estimation of the melting temperature (Tm), which is the temperature at which 50% of the two strands of DNA dissociate, a property dependent on nucleotide composition and product length [48,49]. By monitoring denaturation of the PCR products with SYBR Green and fluorescence levels over a temperature gradient, it is possible to construct the melting curve [50,51]. Confirmation of true positive detections occurred when the Tm of the sample was within 1°C of the true positive control for at least two replicates, and the peak was sharp enough that the absolute value of the slope of the melt curve was greater than 0.6. The DNA source was the original DNA extracted from the gut contents of the predators. For 28 of the 32 species potentially detected as prey, we obtained specimens with confirmed taxonomy to determine a positive control reference Tm to distinguish true and false positive detections. Their DNA was extracted using the DNeasy Blood & Tissue (Qiagen) kit. Species-specific primer pairs were designed as in Paula and Andow [52], nearly all in regions of the mitogenome (primer sequences at Table S3, Supporting Information 2), for all prey species detected by metabarcoding and Lazaro, using the program Primer 3 at Geneious v7.1.9 [53] and checked in NCBI/Primer-BLAST [54]. The cross-reactivity of these primers with related detected species is presented in Supporting Information 1 (Fig. S1 to S4). The qPCR reactions (13 µl) were prepared using Thermo Scientific Maxima SYBR Green/ROX qPCR Master Mix (2×), 1.28 µg/µl of BSA and 10 ng of DNA per reaction and each specific primer pair at 0.3 µM. The amplifications were performed in 384-well plates with a Roche Applied Science LightCycler® 480 (Roche Light Cycler 480 Instrument, RRID:SCR_020502) Real-Time PCR

System using a two-step cycling protocol (initial denaturation at 95°C for 10 min, ramp 4.4°C/s), and 40 cycles of denaturation at 95°C for 15 s (ramp 4.4°C/s) and annealing/extension at 60°C for 60 s (ramp 2.2°C/s), and a melt curve from 60°C to 95°C continuous (ramp 1°C/s) with six readings/°C. qPCR for each sample was performed in at least three technical replicates. No-template controls (NTC) were included for every primer pair. Melt curves were constructed using the raw fluorescence data and diffQ in the library MBmca in *R* [55]. Positive prey detection and identification were considered if at least two technical replicates had -dF/dT more than 0.1 above background or if one technical replicate had -dF/dT more than 0.2 above background at the Tm expected for the prey. When there was no positive control, a sample was considered a true positive if the three amplicon replicates had similar melting curves with the same sharp Tm. The presence of multiple peaks suggests that the PCR amplicons were heterogeneous, and/or possibly mixed with chimeras or primer dimers.

Statistical analysis

For each metabarcoding and Lazaro library, we have the number of species detected, the number of reads for each detected species, and for the field samples, independent confirmation of each detection by MCA. We used MCA to classify true positive (TP) and false positive (FP or type I error) detections and true negative (TN) and false negative (FN or type II error) non-detections. We are considering: TP as detected prey species confirmed by MCA; FP as detected prey species not confirmed by MCA; TN as prey DNA not detected by MCA when the species was not detected by metabarcoding or Lazaro; FN as prey DNA detected by MCA when not detected by metabarcoding or Lazaro. Prey species that were detected by MCA but were not detected by Lazaro, because it did not have a sequence in the respective

DNA reference database, were not considered FNs (the metabarcoding reference database was complete). For the control mock community, we did not need MCA to determine true and false positives and false negatives as the predator feeding history was known.

We calculated the theoretical limit of the detection (LOD) of MCA for all of the species with positive controls by estimating the amount of whole organism template that could be detected at a C_q=40, and calculating the upper 95% confidence interval of the geometric mean of the estimates. In addition, we estimated the amplification efficiency of the MCA primers to ensure it was high enough to amplify rare template sufficiently to detect. The limit of detection of MCA is quite low, but if there is little amount of prey DNA left in the gut, it is also possible that the prey sequence targeted by the species-specific primer pairs of MCA is absent, while other sequences are detected by metabarcoding and or Lazaro. If this were occurring, then for a given prey there should be a lower read count in both metabarcoding and Lazaro when the MCA is negative than when it is positive. We tested this with the prey species for which there were more than three true and false positives in the sample libraries. Number of reads were *ln*-transformed and analyzed by the Welch *t*-test for unequal variance using the Welch-Satterthwaite equation to calculate degrees of freedom.

To compare the performance of metabarcoding and Lazaro, for each library, we also estimated [56,57]:

- *Sensitivity* (or true positive rate), which is the probability that a positive is detected: Sensitivity = TP/(TP+FN);
- *Specificity* (or true negative rate), which is the probability that a negative is not detected: Specificity = TN/(TN+TP)
- *False discovery rate* (FDR), which is the probability that a detection is a false positive:

  FDR = FP/(FP+TP)
- *False omission rate* (FOR), which is the probability that a non-detection is a false negative: FOR = FN/(FN+TN)

- *Accuracy*, which is the probability that detections and non-detections are correct: Accuracy = (TP+TN)/(TP+TN+FP+FN).

Higher sensitivity, specificity and accuracy, and lower false discovery rate and false omission rate are indicative of a better method. Using the aforementioned premises, we tested the following hypotheses:

H_1- Metabarcoding detects a higher number of true positive prey species than Lazaro because the reference database is larger. We tested this by comparing the number of initial detections in a library and the proportion of true positives after confirmation by MCA using a paired *t*-test with the 27 samples as independent observations, predicting that metabarcoding would have more true positives and a higher proportion of true detections;

H_2- Metabarcoding is more prone to false positive prey detections because of amplification bias and the larger reference database. We tested this by comparing the false discovery rate and specificity, predicting that metabarcoding would have a higher false discovery rate and a lower specificity;

H_3- Lazaro is more prone to generate false negatives because lacking the amplification of rare prey DNA fragments, it would be less sensitive. We tested this by comparing the false omission rate and sensitivity, predicting that Lazaro would have a higher false omission rate and lower sensitivity;

H_4- Lazaro enables prey detection with finer taxonomic resolution because the larger reference targets (e.g., mitogenomes) and higher sequencing depth would reduce ambiguity in species identifications. We tested this by comparing the taxonomic resolution of the final prey identifications.

H_5- The number of reads for both metabarcoding and Lazaro are positively related to the probability of a true positive across all prey species and to the relative template concentration for true positives within prey species. We tested the first part of this hypothesis using logistic regression of the *ln*-transform of the number of reads on the binomial variate indicating true positives by MCA versus false positives (logit link, binomial error) with Anova in the package car (type II Wald chi-square) and glm in *Base R*. There were 109 observations for metabarcoding and 116 observations for Lazaro, and no significant overdispersion for either regression. We tested the second part of this hypothesis, i.e., the number of reads is related to the amount of prey DNA in a sample, using the estimated relative template concentration from the qPCR for prey species with at least five true positive detections and variation in both variables of a least 0.5 order of magnitude. This was tested within prey species because amplification efficiency, baselines, and thresholds would be constant for the qPCR. There were three and two species tested for metabarcoding and Lazaro respectively. We calculated the relative initial template concentration from the qPCR amplification curves using LinRegPCR (version 2017.1) with the estimated mean PCR efficiency for each primer pair [58]. Relative initial template concentrations were $\log_{10}$ transformed, number of reads was *ln*-transformed, and data were analyzed with Pearson correlation coefficients using the Fisher transformation to estimate *p*-values.

Results and Discussion

Prey detections from the control mock community

After quality control, the 10 metabarcoding samples (predator guts or libraries) had an average of 2,728,294 reads (8% coefficient of variation [CV]) and the 10 Lazaro samples had an average of 5,355,167 reads (10% CV). None of the samples of predators without any

prey (controls) had any prey detected for either males or females for either metabarcoding or Lazaro. This indicates that extraneous DNA was unlikely to have contaminated the samples during and after the extraction process.

Only Lazaro detected prey species in the control mock community (Table 1; $Accuracy_{female}$=0.71, $Accuracy_{male}$=0.43). For some prey species, only one sex detected it and with few reads (n=2). Although theoretically ecoPCR [38] produced amplicons for the target prey species (maximum number of mismatches allowed per primer: -e=2 and using the # feature to ensure perfect matches in the last two nucleotides at the 3'-end), and the metabarcoding reference database contained their 16S sequences, none of the prey were detected ($Accuracy_{female}$=$Accuracy_{male}$=0), possibly due to the mismatches in at least one primer of the pair (Fig. S5, Supporting Information 1) and preferential amplification of the more abundant predator DNA in the samples. Only the predator was detected by metabarcoding. This illustrates that the insufficient primer universality among taxa can preclude the detection (reduced sensitivity) of expected prey species and that the use of multiple barcodes may be preferable.

Neither metabarcoding nor Lazaro detected any false positives, despite the use of comprehensive reference databases, which increases the likelihood of detecting false positives. In summary, both metabarcoding and Lazaro generated false negatives, with more false negatives and fewer true positives by metabarcoding than Lazaro. These results suggest that neither method on its own will detect all of the prey in a predator gut sample, but that Lazaro may sometimes be more accurate.

Prey detections from field sampled predators

After quality control, the 27 metabarcoding samples (predator guts or libraries) had an average of 2,964,430 reads (12% CV) and the 27 Lazaro samples had an average of 5,504,578 reads (14% CV). The presence of the DNA of each prey species in a predator gut sample was confirmed by MCA [49]. Examples of the various positive and negative prey confirmations by MCA are illustrated in Fig. 1. The differentiation of a true and false prey detection was performed by observing the presence or absence of the sample Tm corresponding to the Tm of the positive control. For example, *So. invicta* true positive control had a Tm of 76.5°C, and false positives had Tm's at 77.9, 79.2, 82.6 and 84.0°C. The theoretical LODs for detection by qPCR amplification were less than 1 pg of whole organism DNA per technical replicate for 28 of the 31 detected prey species, and for 26 of these species it was less than 0.1 pg of whole organism DNA/technical replicate (Table S4, Supporting Information 2). As the DNA templates for MCA are only a small proportion of the whole organism DNA, the LODs indicate that MCA was very sensitive and unlikely to return a false negative for the majority of prey species examined. The amplification efficiency varied from 1.893-1.997 for all of the species-specific primer pairs, which should result in sufficient amplicons for detection by MCA even when the template is rare. Nevertheless, 3 of the 31 detected prey species had higher LOD, which might have resulted in some false negatives: *Selenophorus alternans* (LOD=1.652 pg/technical replicate), *Euschistus heros* (LOD=3.245 pg/technical replicate), and *Cardiocondyla obscurior* (LOD=22.59 pg/technical replicate). Although unlikely, MCA could also give a false negative when the prey DNA was present, but so scarce that there was no MCA template in the sample. In this case, we reasoned that if MCA returned false negatives then the number of reads associated with MCA negatives should be smaller than the number of reads for MCA positives within a prey species. However, only one species (*Ph. tristis* for Lazaro) had fewer reads associated

with negative than with positive MCA detections (Figure S6, Supporting Information 1), indicating that false negative MCA detections were generally not a problem.

Initially, 30 prey were identified, all to species level, by both methods combined prior to verification by MCA (Tables 3 and S5, Supporting Information 2). They were six species of Heteroptera, 10 Hymenoptera (Formicidae), five Coleoptera, three Lepidoptera, two Dermaptera, and a single species of Diptera, Orthoptera, Isoptera and Annelida. Metabarcoding and Lazaro initially detected a similar number of prey species (26 [87%] and 21 [81%], respectively, with 16 species in common), but metabarcoding resulted in more species detections per sample than Lazaro (7.85±0.63 versus 6.78±0.42, respectively, t_{26}=2.08, p-value=0.0479) (Table 3). There were 212 prey detections in the metabarcoding samples, with *ln*-number of reads averaging 7.66 (range 0 to 14.44), and 183 prey detections in the Lazaro samples, with *ln*-number of reads averaging 2.90 (range 0.69 to 8.39). Out of 30 prey species initially detected, 17 species (57%) were confirmed by MCA as prey of the five epigeal predators (Table S5, Supporting Information 2; 14 species by metabarcoding and 13 by Lazaro, with 10 species in common, i.e., 59% of confirmed prey species). Ten of the 13 species not confirmed by MCA were false positives and three species (*Atta sextans*, *Neomegalotomus parvus* and *Strongygaster triangulifera*) were not tested (Table S5, Supporting Information 2). Of the 10 false positive species, five were detected by Lazaro in 15 prey detection instances (8.2% of initial detections), and seven were detected by metabarcoding in 21 prey detection instances (9.9% of initial detections). Most of these false positives were not amplified by MCA or the replicates did not have a consistent Tm. In a few cases the replicates had a consistent Tm, but at the wrong temperature. For example, all of the MCA replicates for the false detections of *M. persicae* gave a consistent signal with a sharp peak, but the peak was >2°C lower than the Tm of the true positive control (Fig. 1). This kind of false positive might have

resulted from taxonomic overclassification [59]. When the prey species is not in the reference database, a closely related species may be identified. These would give false positive species identifications because they were identified beyond the resolution limitation of a reference database. Indeed, the metabarcode amplicon for the false detections of *M. persicae* also had high similarity (≥98%) with several other species of Macrosiphonini, the aphidid tribe of *M. persicae* (BLASTn search, Supporting Information 1). Hence, some false positives might be a true positive prey with a false species determination.

Another possible reason for false positives is contamination of the samples after DNA extraction. This could occur during any of the post-extraction procedures, including library preparation and sequencing. If such contamination had occurred, either or both metabarcoding and Lazaro might detect the contaminant with substantial numbers of reads, but MCA would not, because the contaminant would not be in the original DNA extracted sample. For example, many of the false positive detections of the coleopterans *Anthonomus grandis* (Table S5 Supporting Information 2, 19 samples, 47,626 metabarcoding amplicons, 142 Lazaro reads) and *Harmonia axyridis* (Table S5 Supporting Information 2, 8,919,830 metabarcoding amplicons, 18 samples, 1,190 Lazaro reads) had these characteristics, and may be post-extraction contaminants. The presence of *An. grandis* at the experimental site was unlikely as it is a specific cotton herbivore and none of the experimental plots had cotton, however, this species is mass-reared in a nearby laboratory, which may have been the source of contamination. False positive detections can also occur when the prey species is closely related to the predator. An example was the false detection by metabarcoding of *So. richteri* in the gut content of the predator *So. substituta* (Table S5, Supporting Information 2). It may be difficult with metabarcoding to resolve taxonomically closely related species.

Regarding false negative prey detection, there were 11 false negatives for metabarcoding and 11 false negatives for Lazaro (Table 4). For example, *M. spectabilis* was not detected as prey by metabarcoding in two samples, and *Sp. frugiperda* was not detected as prey using Lazaro in one sample (Table S5, Supporting Information 2), but they were detected by MCA. False negatives could have been generated for rare prey in the samples with a large number of pooled individuals (Table 5). For example, for the Lazaro samples, coverage ranged from 20,000 to 120,000 reads/individual, and the number of detected prey reads was only 1.3 to 29.2/individual. Thus, rare prey may be missed (false negative) in the Lazaro samples. The metabarcoding samples had coverage ranging from 11,000 to 64,000 amplicons/individual, and the number of detected prey amplicons ranged from 114 to 40,000/individual. Thus, rare prey may have been missed because of the large numbers of individuals in a sample. However, as the three-ant species are known to recruit large numbers of individuals to harvest prey, rare prey are likely to occur in multiple individuals, and may be unlikely to be missed. Moreover, if false negatives were primarily related to missing rare prey, then the false omission rate should be negatively correlated with coverage or prey detection. This was not observed (Tables 5 and S6, Supporting Information 2), hence, while some rare prey may have been missed, they were equally likely to have been missed by both metabarcoding and Lazaro. Finally, it is also possible that false positives and negatives can occur because the taxonomy of the reference genetic material at GenBank was incorrectly identified.

Species identifications have been demonstrated to differ when using different DNA extraction protocols, DNA polymerases, amplification parameters, reference databases or barcodes, and even when using different primers from the same barcode [9,13,60-63], and the results from our control mock community also showed that different DNA-based detection methods differed in the species identified. So, the incongruent prey species detection between metabarcoding and Lazaro may not be unusual and additional possible

reasons are discussed below. Nonetheless, the underlying consequence is that the ecological inferences are likely to be affected by the prey detection method used as the predator food webs would have different structures (Fig. 2). Specifically, the food webs of three of the predators would be different using only metabarcoding or Lazaro. These results highlight the need for precaution when comparing the data between eDNA studies to enable robust ecological comparisons [64,65]. Indeed, as the two methods appear to be partially complementary, using both may provide more robust results.

OTU analysis could be conducted on the reads that did not match satisfactorily with any species in the DNA reference databases to complement the prey diversity analysis. The number of 'unassigned' reads was fairly high in both methods, but not surprisingly, more prominent in Lazaro. The percent of reads of confirmed prey detections across all 27 predator samples was 45% for metabarcoding and less than 1% for Lazaro. The majority of the 'unassigned' reads were related to the predator DNA (e.g., nuclear DNA), even though we reduced the amount of predator biomass by gut dissection or gaster removal (for ants). Another part of the 'unassigned' reads could be related to predator symbionts or parasites or other exogenous species that were not present in the reference databases used in this work. Although high, the 99% of 'unassigned' reads for the Lazaro is not unexpected for two reasons: we only worked with mitochondrial reads and only for the arthropod species preyed upon by the predator, but not the predator mitochondrial reads. It is known that the proportion of mitochondrial reads obtained in NGS sequencing of whole macerated organisms or tissues without *in vitro* mitochondrial enrichment is only about 1% [19,29]. The predator mitochondrial reads should not be considered 'unassigned' reads in the strict sense, nevertheless they were not included in the assigned reads because there is no means to differentiate the predator reads with reads from a cannibalized conspecific, especially in our samples comprised of large pools of individuals. In a similar way, the prevalence of 55% of

'unassigned' reads for the metabarcoding data was not unexpected because no specific predator blocking primers were used to preclude or minimize amplification of the predator template. Similar to Lazaro, most of the 'unassigned' reads were predator 16S amplicons. Our choice to not use specific predator blocking primers was based on Piñol et al. [66], who demonstrated that predator blocking primers may coblock the amplification of prey species closely related to the predator, which is more critical when analyzing diet composition of arthropod generalist predators.

Metabarcoding versus Lazaro

To compare which method, metabarcoding or Lazaro, resulted in better prey determination, we evaluated the sensitivity, specificity, false discovery rate, false omission rate and accuracy of prey determination for the field sampled predators and accuracy of prey determination for the mock community. In addition, for the field sampled predators we determined the relation between the number of reads for a prey and the amount of prey DNA in the samples (feeding bioassay controls were not analyzed this way because metabarcoding did not detect prey reads). Specifically, we tested the five hypotheses discussed below.

H_1- Metabarcoding detects more true positive prey species than Lazaro. Contrary to this hypothesis, in the field sampled predators, metabarcoding and Lazaro had a similar number of confirmed prey per sample (1.81±0.24 and 1.85±0.23, respectively, t_{26}=-0.44, *p*-value=0.6632) and of the initial prey tested by MCA, the same false discovery rate ((0.68±0.033 versus 0.67±0.031, respectively; *p*-value=0.3929, Table 6). The rejection of H_1 was corroborated by the results from the mock community for which no true positives were detected by metabarcoding.

True positives might be increased for metabarcoding by sequencing separately PCR replicates for each barcode per sample [67], using a multi-level assignment approach [68] or pre-testing the metabarcode primer to ensure amplification of expected prey and reduced amplification of the predator [69]. This last approach may hamper the detection of prey species closely related to the predator. While there are a number of publications showing that the use of multiple metabarcode primers increase the diversity of prey detection [e.g., 40], they typically do not evaluate if false prey detection also increases, as might be expected. In addition, there is no guarantee that the use of multiple primers will always improve metabarcoding performance. To illustrate this point, we show the putative mismatches of the 16S barcode primer pair that we used and three commonly used COI barcode primer pairs (LCO1490 and HCO2198 [70]; mICOIintF and jgHCO2198 [71]; UniMiniBar [72]) with the prey species used in the mock community and detected in the field samples (Fig. S5, Supporting Information 1). LCO1490/HCO2198 and UniMiniBar had a higher number of template mismatches than the 16S primers and probably would have detected fewer prey species than the 16S primer pair. The forward primer of the other COI primer pair had mismatches in the critical last three bases of the 3'-end with all six prey species in the mock community and in eight of 29 species (27%) detected in the field samples.

True positives might be increased for Lazaro by using multiple reference databases. In this work, we used only a mitogenome reference database, but it is also possible to use rDNA and symbionts [28, 29] and even unassembled reads [73] in a reference database. These databases can be constructed and used at any time after sequencing, unlike metabarcoding.

H_2- Metabarcoding is more prone to false positive prey detections than Lazaro. Both the false discovery rate, and the specificity (0.58±0.026 versus 0.62±0.022, respectively; *p*-value=0.2601, Table 6) were similar for metabarcoding and Lazaro, so H_2 was rejected.

While it was true that metabarcoding detected on average 350 times more true prey reads than Lazaro (Table S5, Supporting Information 2), this did not convert into a higher detection of true positive or a significantly higher detection of false positive prey detections compared to Lazaro.

H_3- Lazaro is more prone to have false negatives than metabarcoding. For the field sampled predators, false omission rate for Lazaro was similar to that for metabarcoding (0.068±0.019 versus 0.073±0.019, respectively; *p*-value=0.649; Table 6). Similarly, Lazaro did not have lower sensitivity than metabarcoding (0.814±0.059 versus 0.806±0.059, respectively; *p*-value=0.7874; Table 6), so H_3 was rejected. The rejection of H_3 was corroborated by the results from the control mock community. A factor that may have contributed to false negative detections in some predators is that prey DNA is in an advanced state of degradation, precluding PCR amplification (in the case of metabarcoding) or being excluded by size selection during library construction, but still possible to be detected by MCA because of the smaller length of the target amplicon (between 100 to 200 bp, Table S3, Supporting Information 2). In the case of metabarcoding, it could also be related to insufficient complementarity between template and metabarcoding primers, precluding the representation of a prey species or taxonomic group in the sample, or preferential amplification of the more common predator DNA, resulting in poor amplification of prey DNA. Realistically, it is quite likely that the number of false negatives might be even higher, because we could not check all species co-occurring in the sample area because we could not be sure that all species were in the reference databases.

With H_1, H_2 and H_3 rejected, it follows that metabarcoding and Lazaro had similar accuracy in prey detection (0.62±0.024 versus 0.66±0.020, respectively; *p*-value=0.1769; Table 6). This differs from the results from the control mock community and in Srivathsan et

al. [26]. The accuracy in the control mock community was 0.00 for metabarcoding versus 0.43-0.71 for Lazaro (log-linear model g^2=9.36, p-value=0.0022). Srivathsan et al. [26] compared metabarcoding and metagenomics (using BLASTn) to identify diet composition by fecal analysis (host plant chloroplasts) of two red-shanked doucs langurs (*Pygathrix nemaeus*) fed with a known diet. While metabarcoding detected 34% of the diet composition, metagenomics detected 50% of the known diet plus an unexpected species that was later confirmed to be in the diet.

H_4- Lazaro enables prey detection with finer taxonomic resolution than metabarcoding. For the field sampled predators, all confirmed species identifications were at the species level for both methods. Thus, in our field samples, taxonomic resolution was the same, and H_4 was rejected.

H_5- The number of reads for both metabarcoding and Lazaro are positively related to the probability of a true positive across all prey species and to the relative template concentration for true positives within prey species. Logistic regression showed that the probability of the true positive was not related to the number of reads for metabarcoding (regression coefficient =0.11±0.07, χ^2 =2.52, p-value=0.1122) but was highly positively related to the number of reads for Lazaro (regression coefficient =0.49±0.12, χ^2 =15.87, p-value=6.798E-5). For a prey species in the field samples of predators, the tests determined if the number of reads was correlated with the amount of prey in the predator, as measured by qPCR (Table 7). For Lazaro, H_5 was accepted as there was a positive correlation between the number of reads and the relative template concentration in the samples for both species that could be analyzed. However, for metabarcoding, H_5 was rejected for three of the four species as there was no correlation between the number of reads and the relative template concentration for these species. The interpretation of number of reads from the metabarcoding results has been controversial

[74-79], and our results provide some support for the argument that the number of metabarcoding reads is an unreliable predictor of the DNA quantity in a sample but the number of Lazaro reads might be a good predictor.

In terms of cost, metabarcoding has the potential to cost about half that of Lazaro. In this study, we chose not to multiplex the 27 samples for metabarcoding analysis to keep the coverage per sample similar between methods. The costs that were the same for both methods were: sample preparation (USD10); total DNA extractions (USD100); library construction (USD85.50/each) and HiSeq4000 sequencing lane (USD4,310). For metabarcoding, there were additional costs for primer synthesis, PCR reactions, purification and quantifications for each sample, which were estimated to be USD160. If we had multiplexed the 27 purified sample amplicons in one library, the total cost of metabarcoding would have been USD2,510.50. For the Lazaro method, samples cannot be multiplexed and the total cost was USD4,573.50.

Conclusions

Metabarcoding and Lazaro identified a range of prey species that were preyed upon by arthropod epigeal predators, but they were partially complementary methods sharing 87% of true positive detections. Both methods crucially depended on the comprehensiveness of their respective DNA reference databases, which for metabarcoding was undeniably larger. Even so, Lazaro determined prey with similar specificity, sensitivity, false discovery and omission rates, accuracy, and taxonomic resolution as metabarcoding. The use of multiple barcodes in the metabarcoding analysis could render higher sensitivity, although it could also increase false positives (reduce specificity) as each primer pair carries its own associated bias. One may prefer Lazaro because it preserves the original sample DNA

community, enabling further search for other targets (host plants, symbionts, parasites, etc), using any other DNA reference database(s) and the number of reads was associated with the quantity of prey DNA in the predators, while for metabarcoding prey detection is constrained by the initial chosen barcodes. However, one may prefer metabarcoding because it remains less expensive than Lazaro for processing a large set of samples as they can be multiplexed in a single library. In addition, it is easier to enrich a reference database by elucidating barcode sequences, which usually are fragments of a gene, than elucidating an organellar genome.

Data Availability

Library sequencing datasets were deposited at GenBank and their Sequence Read Archive (SRA) access codes are in Supporting Information 1. Supporting data and materials are available in the *GigaDB* database [43].

Additional Files

Supporting Information 1 (Word file): Figures S1 to S5.

Supporting Information 2 (Excel file): Tables S1 to S6.

List of abbreviations

Abbreviations used in the text are defined in the text at first use.

Consent for publication

Not applicable.

Competing interests

The authors declare that they have no competing interests.

Funding

This work was funded by the grant USDA-NIFA 2016-67030-24950.

Author's contributions

Design of study: DPP, DAA, RMP, MRB
Collection and preparation of samples: SKAB, RMP, DPP
Data analyses (bioinformatic, qPCR, statistical): RCT, DPP, DAA
Writing of the manuscript: DPP, DAA, RCT, RMP

Acknowledgements

We would like to thank Jian Chen, Micky Eubanks, Hannah Gray, Fangneng Huang and Michael Strand for the donation of identified specimens to use as positive controls for MCA in qPCR analysis.

References

[1] Taberlet P, Coissac E, Pompanon F, Brochmann C, Willerslev E. Towards next-generation biodiversity assessment using DNA metabarcoding. Mol Ecol. 2012;21(8):2045-50.

[2] Clare EL. Molecular detection of trophic interactions: emerging trends, distinct advantages, significant considerations and conservation applications. Evol Appl. 2014;7(9):1144-57.

[3] Paula DP. Next-generation sequencing and its impacts on entomological research in ecology and evolution. Neotrop Entomol. 2021;50:679-96.

[4] Schloss PD, Westcott SL, Ryabin T, et al. Introducing mothur: open-source, platform-independent, community-supported software for describing and comparing microbial communities. Appl Environ Microbiol. 2009;75:7537-41.

[5] Caporaso JG, Kuczynski J, Stombaugh J, et al. QIIME allows analysis of high-throughput community sequencing data. Nat Methods 2010;7:335-6.

[6] Boyer F, Mercier C, Bonin A, Le Bras Y, Taberlet P, Coissac E. Obitools: a unix-inspired software package for DNA metabarcoding. Mol Ecol Resour. 2016;16:176-82.

[7] Callahan BJ, McMurdie PJ, Rosen MJ, Han AW, Johnson AJA, Holmes SP. DADA2: high-resolution sample inference from Illumina amplicon data. Nat Methods 2016;13:581-3.

[8] Anslan S, Bahram M, Hiiesalu I, Tedersoo L. PipeCraft: flexible open-source toolkit for bioinformatics analysis of custom high-throughput amplicon sequencing data. Mol Ecol Resour. 2017;17(6):e234.

[9] Taberlet P, Bonin A, Zinger L, Coissac E. Environmental DNA: for biodiversity research and monitoring. 1st ed. Oxford University Press; 2018.

[10] Deagle BE, Jarman SN, Coissac E, Pompanon F, Taberlet P. DNA metabarcoding and the COI marker: not a perfect match. Biol Lett. 2014;10:20140562.

[11] Clarke LJ, Soubrier J, Weyrich LS, Cooper A. Environmental metabarcodes for insects: *in silico* PCR reveals potential for taxonomic bias. Mol Ecol Resour. 2014;14:1160-70.

[12] Elbrecht V, Leese F. Can DNA-based ecosystem assessments quantify species abundance? Testing primer bias and biomass-sequence relationships with an innovative metabarcoding protocol. PLoS One 2015;10:e0130324.

[13] Elbrecht V, Leese F. Validation and development of COI metabarcoding primers for freshwater macroinvertebrate bioassessment. Front Environ Sci. 2017;5:11.

[14] Haas BJ, Gevers D, Earl AM, et al. Chimeric 16S rRNA sequence formation and detection in Sanger and 454-pyrosequencing PCR amplicons. Genome Res. 2011;21:494.

[15] Zhou X, Li Y, Liu S, et al. Ultra-deep sequencing enables high-fidelity recovery of biodiversity for bulk arthropod samples without PCR amplification. GigaScience 2013;2:4.

[16] Gillett CP, Crampton-Platt A, Timmermans MJTN, et al. Bulk *de novo* mitogenome assembly from pooled total DNA elucidates the phylogeny of weevils (Coleoptera: Curculionoidea). Mol Biol Evol. 2014;31:2223-37.

[17] Tang M, Tan M, Meng G, et al. Multiple, sequencing of pooled mitochondrial genomes - a crucial step toward biodiversity analysis using mito-metagenomics. Nucleic Acids Res. 2014;42(22):e166.

[18] Andujar C, Arribas P, Ruzicka F, et al. Phylogenetic community ecology of soil biodiversity using mitochondrial metagenomics. Mol Ecol. 2015;24:3603-17.

[19] Crampton-Platt AL, Timmermans MJTN, Gimmel ML, et al. Soup to tree: the phylogeny of beetles inferred by mitochondrial metagenomics of a Bornean rainforest sample. Mol Biol Evol. 2015;32(9):2302-16.

[20] Gomez-Rodrıguez C, Crampton-Platt A, Timmermans MJ, et al. Validating the power of mitochondrial metagenomics for community ecology and phylogenetics of complex assemblages. Meth Ecol Evol. 2015;6(8):883-94.

[21] Liu S, Wang X, Xie L, et al. Mitochondrial capture enriches mito-DNA 100 fold, enabling PCR-free mitogenomics biodiversity analysis. Mol Ecol Resour. 2016;16:470-9.

[22] Linard B, Crampton-Platt A, Timmermans MJTN, Vogler AP (2015) Metagenome skimming of insect specimen pools: potential for comparative genomics. Gen Biol Evol. 2015;7(6):1474-89.

[23] Shokralla S, Gibson J, King I, et al. Environmental DNA barcode sequence capture: targeted, PCR-free sequence capture for biodiversity analysis from bulk environmental samples. 2016. https://www.biorxiv.org/content/10.1101/087437v1.

[24] Sarmashghi S, Bohmann K, Gilbert MTP, et al. Skmer: assembly-free and alignment-free sample identification using genome skims. Genome Biol. 2019;20(1):1-20.

[25] Ji Y., Huotari T, Roslin T, et al. SPIKEPIPE: A metagenomic pipeline for the accurate quantification of eukaryotic species occurrences and intraspecific abundance change using DNA barcodes or mitogenomes. Mol Ecol Resour. 2020;20(1):256-67.

[26] Srivathsan A, Sha JCM, Vogler AP, Meier R. Comparing the effectiveness of metagenomics and metabarcoding for diet analysis of a leaf feeding monkey (*Pygathrix nemaeus*). Mol Ecol Resour. 2015;15:250-61.

[27] Srivathsan A, Ang A, Vogler AP, Meier R. Fecal metagenomics for the simultaneous assessment of diet, parasites, and population genetics of an understudied primate. Front Zool. 2016;13:17.

[28] Paula DP, Linard B, Andow DA, Sujii ER, Pires CSS, Vogler AP. Detection and decay rates of prey and prey symbionts in the gut of a predator through metagenomics. Mol Ecol Resour. 2015;15:880-92.

[29] Paula DP, Linard B, Platt AC, Srivathsan A, Timmermans M, Sujii E, Pires C, Machado L, Andow DA, Vogler A. Uncovering trophic interactions in arthropod predators through DNA shotgun-sequencing of gut contents. PLoS ONE 2016; 11:e0161841.

[30] Aquino AM, Aguiar-Menezes EL, Queiroz JM. Recomendações para coleta de artrópodes terrestres por armadilhas de queda (“pitfall-traps”). Circular Técnica. 16. Embrapa. Rio de Janeiro. 2006; 8p.

[31] Sutherland WJ. Ecological census techniques: a handbook. 2nd ed. Cambridge: Cambridge University; 1996.

[32] Greenstone MH, Weber DC, Coudron TA, Payton ME, Hu JS. Removing external DNA contamination from arthropod predators destined for molecular gut-content analysis. Mol Ecol Resour. 2012;12(3):464-9.

[33] Zaidi RH, Jaal Z, Hawkes NJ, Hemingway J, Symondson WO. Can multiple-copy sequences of prey DNA be detected amongst the gut contents of invertebrate predators? Mol Ecol. 1999;8(12):2081-7.

[34] Elbrecht V, Taberlet P, Dejean T, et al. Testing the potential of a ribosomal 16S marker for DNA metabarcoding of insects. PeerJ 2016;4:e1966.

[35] Sousa LL, Silva SM, Xavier R. DNA metabarcoding in diet studies: unveiling ecological aspects in aquatic and terrestrial ecosystems. Environmental DNA 2019;1(3):199-14.

[36] O’Donnell JL, Kelly RP, Lowell NC, Port JA. Indexed PCR primers induce template-specific bias in large-scale DNA sequencing studies. PLoS ONE 2016;11(3):e0148698.

[37] Juen A, Traugott M. Amplification facilitators and multiplex PCR: Tools to overcome PCR-inhibition in DNA-gut-content analysis of soil-living invertebrates. Soil Biol Biochem. 2006;38(7):1872-9.

[38] Ficetola GF, Coissac E, Zundel S, et al. An *in silico* approach for the evaluation of DNA barcodes. BMC Genomics 2010;11:e434.

[39] Stoesser G, Moseley MA, Sleep J, et al. The EMBL Nucleotide Sequence Database. Nucleic Acids Res. 1998;26:8-15.

[40] De Barba M, Miquel C, Boyer F, Mercier C, Rioux D, Coissac E, Taberlet P. DNA metabarcoding multiplexing and validation of data accuracy for diet assessment: application to omnivorous diet. Mol Ecol Resour. 2014;14:306-23.

[41] Quéméré E, Hibert F, Miquel C, et al. A DNA metabarcoding study of a primate dietary diversity and plasticity across its entire fragmented range. PLoS ONE. 2013;8(3):e58971.

[42] Paula DP, Timbó RV, Togawa RC, Vogler AP, Andow DA Quantitative prey species detection in predator guts across multiple trophic levels by DNA shotgun sequencing. bioRxiv. 2021. https://doi.org/10.1101/2021.04.01.438119.

[43] Paula DP, Barros SKA, Pitta RM, Barreto M, Togawa RC, Andow DA. Supporting data for "Metabarcoding versus mapping unassembled shotgun reads for identification of prey consumed by arthropod epigeal predators". GigaScience Database. 2022; http://doi.org/10.5524/100970.

[44] Andrews D. (2010) FastQC: a quality control tool for high throughput sequence data. http://www.bioinformatics.babraham.ac.uk/projects/fastqc

[45] Aronesty E. (2011) ea-utils: Command-line tools for processing biological sequencing data. https://github.com/ExpressionAnalysis/ea-utils.

[46] Martin M. Cutadapt removes adapter sequences from high-throughput sequencing reads. EMBnet.journal 2011;17(1):10-2.

[47] Shen W, Le S, Li Y, Hu F. SeqKit: a cross-platform and ultrafast toolkit for FASTA/Q file manipulation. PLoS ONE 2016;11(10):e0163962.

[48] Ririe KM, Rasmussen RP, Wittwer CT. Product differentiation by analysis of DNA melting curves during the polymerase chain reaction. Anal Biochem 1997;245:154-60.

[49] Zhang T, Fang HH. 16S rDNA clone library screening of environmental sample using melting curve analysis. Journal of the Chinese Institute of Engineers 2005;28:1153-5.

[50] Winder L, Phillips C, Richards N, Ochoa-Corona F, Hardwick S, Vink CJ, Goldson S. Evaluation of DNA melting analysis as a tool for species identification. Methods Ecol Evol. 2011; 2:312-20.

[51] Perera OP, Allen KC, Jain D, Purcell M, Little NS, Luttrell RG. Rapid Identification of *Helicoverpa armigera* and *Helicoverpa zea* (Lepidoptera: Noctuidae) Using Ribosomal RNA Internal Transcribed Spacer 1. J Insect Sci. 2015;15(1):155.

[52] Paula DP, Andow DA Melting curve analysis for detection and identification of ghost parasitoids in host carcasses a month after host death. Methods Ecol Evol. 2021;12(9):1552-61.

[53] Kearse M, Moir R, Wilson A, et al. Geneious Basic: an integrated and extendable desktop software platform for the organization and analysis of sequence data. Bioinformatics 2012;28(12):1647-9.

[54] Ye J, Coulouris G, Zaretskaya I, Cutcutache I, Rozen S, Madden T. Primer-BLAST: A tool to design target-specific primers for polymerase chain reaction. BMC Bioinformatics 2012;13:134.

[55] R Core Team. R: A language and environment for statistical computing. R Foundation for Statisical Computing, Vienna, Austria, 2019.

[56] Altman DG, Bland JM. Diagnostic tests. 1: Sensitivity and specificity. BMJ. 1994;308(6943):1552.

[57] Fletcher RH, Fletcher SW, Fletcher GS. Clinical epidemiology: the essentials. 4th ed. Lippincott Williams & Wilkins; 2005.

[58] Ruijter JM, Ramakers C, Hoogaars WMH, Karlen Y, Bakker O, van den Hoff MJB, Moorman AFM. Amplification efficiency: linking baseline and bias in the analysis of quantitative PCR data. Nucleic Acids Res. 2009;37:e45.

[59] Richardson RT, Bengtsson-Palme J, Johnson RM. Evaluating and optimizing the performance of software commonly used for the taxonomic classification of DNA metabarcoding sequence data. Mol Ecol Resour. 2017;17:760-9.

[60] Meusnier I, Singer GA, Landry JF, Hickey DA, Hebert PD, Hajibabaei M. A universal DNA mini-barcode for biodiversity analysis. BMC Genom. 2008;9:214.

[61] Leray M, Yang JY, Meyer CP, Mills SC, Agudelo N, Ranwez V, et al. A new versatile primer set targeting a short fragment of the mitochondrial COI region for metabarcoding metazoan diversity: application for characterizing coral reef fish gut contents. Front Zool. 2013;10(1):34.

[62] Gibson J, Shokralla S, Porter TM, King I, van Konynenburg S, Janzen DH, Hallwachs W, Hajibabaei M. Simultaneous assessment of the macrobiome and microbiome in a bulk sample of tropical arthropods through DNA metasystematics. PNAS 2014;111(22):8007-12.

[63] Nichols RV, Vollmers C, Newsom LA, Wang Y, Heintzman PD, Leighton M, Green RE, Shapiro B. Minimizing polymerase biases in metabarcoding. Mol Ecol Resour. 2018;18:927-39.

[64] Nilsson RH, Tedersoo L, Lindahl BD, et al. Towards standardization of the description and publication of next-generation sequencing datasets of fungal communities. New Phytol. 2011;191:314-8.

[65] Tedersoo L, Ramirez KS, Nilsson RH, Kaljuvee A, Kõljalg U, Abarenkov K. Standardizing metadata and taxonomic identification in metabarcoding studies. GigaScience 2015;4(1):s13742-015-0074-5.

[66] Piñol J, San Andrés V, Clare EL, Mir G, Symondson WO. A pragmatic approach to the analysis of diets of generalist predators: The use of next-generation sequencing with no blocking probes. Mol Ecol Resour. 2014;14(1):18-26.

[67] Robasky K, Lewis NE, Church GM. The role of replicates for error mitigation in next-generation sequencing. Nat Rev Genet. 2014;15(1):56-62.

[68] Munch K, Boomsma W, Huelsenbeck JP, Willerslev E, Nielsen R. Statistical assignment of DNA sequences using Bayesian phylogenetics. Syst Biol. 2008;57(5):750-7.

[69] Alberdi A, Aizpurua O, Gilbert MT, Bohmann K. Scrutinizing key steps for reliable metabarcoding of environmental samples. Methods Ecol Evol. 2018;9(1):134-47.

[70] Folmer O, Black M, Hoeh W, Lutz R, Vrijenhoek R. DNA primers for amplification of mitochondrial cytochrome c oxidase subunit I from diverse metazoan invertebrates. Mol Mar Biol Biotechnol. 1994;3(5):294-9.

[71] Leray M, Yang JY, Meyer CP et al. A new versatile primer set targeting a short fragment of the mitochondrial COI region for metabarcoding metazoan diversity: application for characterizing coral reef fish gut contents. Front Zool. 2013;10:34.

[72] Meusnier I, Singer GAC, Landry JF, Hickey DA, Hebert PDN, Hajibabaei M: A universal DNA mini-barcode for biodiversity analysis. BMC Genomics 2008;9:214.

[73] Sarmashghi S, Bohmann K, Gilbert MTP, Bafna V, Mirarab S. Skmer: assembly-free and alignment-free sample identification using genome skims. Genome Biol. 2019;20(1):1-20.

[74] Deagle BE, Thomas AC, McInnes JC, Clarke LJ, Vesterinen EJ, et al. Counting with DNA in metabarcoding studies: how should we convert sequence reads to dietary data? Mol Ecol. 2019;28:391-40.

[75] Piñol J, Mir G, Gomez-Polo P, Agustí N. Universal and blocking primer mismatches limit the use of high throughput DNA sequencing for the quantitative metabarcoding of arthropods. Mol Ecol Resour. 2015;15:1-12.

[76] Piñol J, Senar MA, Symondson WO. The choice of universal primers and the characteristics of the species mixture determines when DNA metabarcoding can be quantitative. Mol Ecol.2018;28, 407-19.

[77] Thomas AC, Deagle BE, Eveson JP, Harsch CH, Trites AW. Quantitative DNA metabarcoding: improved estimates of species proportional biomass using correction factors derived from control material. Mol Ecol Resour. 2016;16(3):714-26.

[78] Bista I, Carvalho GR, Tang M, et al. Performance of amplicon and shotgun sequencing for accurate biomass estimation in invertebrate community samples. Mol Ecol Resour. 2017;18:1020-34.

[79] Lamb PD, Hunter E, Pinnegar JK, Creer S, Davies RG, Taylor MI. How quantitative is metabarcoding: A meta-analytical approach. Mol Ecol. 2019;28(2):420-30.**Table 1.** Number of reads detected for the control mock community by mapping unassembled shotgun reads (Lazaro) and metabarcoding (16S barcode) using the harlequin *Harmonia axyridis* (48 h after adult emergence with no food) as predator and six prey species, consumed at once in an interval of one hour. Gut contents of the predators were analyzed after six hours after feeding on the last prey item. The threshold used for Lazaro was 100% identity in a minimum overlap of 130 bp and for metabarcoding was 98% identity for an amplicon between 180-230 bp. Mb: metabarcoding; L: Lazaro.

	Predator	Prey

Predator sex	Time (h) after feeding	***Harmonia axyridis***		***Acyrthosiphon pisum***		***Aphis craccivora***		***Aphis glycines***		***Aphis gossypii***		***Myzus persicae***		***Cycloneda munda***	
		**Mb**	**L**	**Mb**	**L**	**Mb**	**L**	**Mb**	**L**	**Mb**	**L**	**Mb**	**L**	**Mb**	**L**
Female	-	114,033	18,984	0	0	0	0	0	0	0	0	0	0	0	0
	6 h	128,773±11,714.9	40,498±9,556.9	0	16	0	26	0	2	0	2	0		0	6
Male	-	130,314	35,340	0	0	0	0	0	0	0	0	0	0	0	0
	6 h	451,570.75±371,433.7	43,452±9,443.5	0	10	0	4	0	0	0	0	0	10	0	0

Table 2. Species detected as prey of epigeal arthropod predators by metabarcoding or Lazaro or both in at least one of 27 libraries before verification by Melting Curve Analysis (MCA) in qPCR. Species with the name in bold are the ones with detection confirmed by MCA in at least one library. All these species are likely to occur in the sampling area/period.

**Order**	**Species (Family)**	**Detection method(s)**	**# reads**	**# libraries**	**Predator**
Annelida	*Phascolosoma esculenta* (Phascolosomatidae)	Metabarcoding	21,938	13	*Dorymyrmex brunneus*, *Tetracha* sp.
Coleoptera	***Anthonomus grandis* (Curculionidae)**	Both	946,283	27	*Pheidole flavens*, *Dorymyrmex brunneus*, *Solenopsis substituta*, *Tetracha* sp., *Euborellia annulipes*
	Eriopis connexa (Coccinellidae)	Both	2,706	5	*Dorymyrmex brunneus*, *Solenopsis substituta*, *Tetracha* sp., *Euborellia annulipes*
	***Harmonia axyridis* (Coccinellidae)**	Both	12,800,160	27	*Pheidole flavens*, *Dorymyrmex brunneus*, *Solenopsis substituta*, *Tetracha* sp., *Euborellia annulipes*
	Selenophorus alternans (Carabidae)	Both	1,639,746	9	*Pheidole flavens*, *Dorymyrmex brunneus*, *Solenopsis substituta*, *Tetracha* sp.
	Tetracha brasiliensis (Carabidae)	Lazaro	2	1	*Solenopsis substituta*
Dermaptera	***Doru luteipes* (Forficulidae)**	Both	1,808	7	*Pheidole flavens*, *Dorymyrmex brunneus*, *Euborellia annulipes*
	***Euborellia annulipes* (Anisolabididae)**	Both	41,492	12	*Pheidole flavens*, *Dorymyrmex brunneus*, *Solenopsis substituta*, *Tetracha* sp.
Diptera	*Strongygaster triangulifera* (Tachinidae)	Both	62	2	*Tetracha* sp., *Euborellia annulipes*
Hemiptera	***Chinavia impicticornes* (Pentatomidae)**	Both	24,621	10	*Dorymyrmex brunneus*, *Solenopsis substituta*, *Tetracha* sp., *Euborellia annulipes*
	***Euschistus heros* (Pentatomidae)**	Both	267,209	5	*Dorymyrmex brunneus*, *Tetracha* sp.
	***Mahanarva spectabilis* (Cercopidae)**	Lazaro	12	2	*Solenopsis substituta*, *Tetracha* sp.
	Neomegalotomus parvus	Metabarcoding	365	1	*Tetracha* sp.
	Myzus persicae (Aphididae)	Metabarcoding	1,696	2	*Dorymyrmex brunneus*

	Planicephalus flavicosta (Cicadellidae)	Metabarcoding	367	1	*Dorymyrmex brunneus*
Hymenoptera	*Atta sextans* (Formicidae)	Both	2,291	6	*Dorymyrmex brunneus*
	Brachymyrmex patagonicus (Formicidae)	Lazaro	24	11	*Pheidole flavens*, *Dorymyrmex brunneus*, *Tetracha* sp., *Euborellia annulipes*
	***Cardiocondyla obscurior* (Formicidae)**	Both	5,576	3	*Pheidole flavens*, *Solenopsis substituta*
	***Dorymyrmex brunneus* (Formicidae)**	Both	3,829	15	*Pheidole flavens*, *Solenopsis substituta*, *Tetracha* sp., *Euborellia annulipes*
	***Pheidole flavens* (Formicidae)**	Both	244,820	15	*Dorymyrmex brunneus*, *Solenopsis substituta*, *Tetracha* sp., *Euborellia annulipes*
	***Pheidole obscurithorax* (Formicidae)**	Both	36	4	*Pheidole flavens*, *Dorymyrmex brunneus*
	***Pheidole oxyops* (Formicidae)**	Both	9,015,220	27	*Pheidole flavens*, *Dorymyrmex brunneus*, *Solenopsis substituta*, *Tetracha* sp., *Euborellia annulipes*
	***Pheidole tristis* (Formicidae)**	Both	2,795,078	27	*Pheidole flavens*, *Dorymyrmex brunneus*, *Solenopsis substituta*, *Tetracha* sp., *Euborellia annulipes*
	Solenopsis richteri (Formicidae)	Metabarcoding	75,556	1	*Solenopsis substituta*
	Solenopsis substituta (Formicidae)	Both	608	9	*Pheidole flavens*, *Dorymyrmex brunneus*
Isoptera	***Syntermes spinosus* (Termitidae)**	Metabarcoding	897	1	*Dorymyrmex brunneus*
Lepidoptera	***Chrysodeixis includens* (Noctuidae)**	Both	45	1	*Tetracha* sp.
	Glena unipennaria (Geometridae)	Both	280	1	*Tetracha* sp.
	***Spodoptera frugiperda* (Noctuidae)**	Metabarcoding	8,448	11	*Dorymyrmex brunneus*, *Tetracha* sp.
Orthoptera	*Gryllus argentinus* (Gryllidae)	Metabarcoding	32	1	*Dorymyrmex brunneus*

Table 3. Number of prey species identified by metabarcoding and Lazaro before verification and proportion verified by Melting Curve Analysis (MCA) in qPCR. Mb: metabarcoding; L: Lazaro.

Library	Predator Species	Original			Proportion verified by MCA		
		Mb	L	Both	Mb	L	Both
0.40	*Pheidole flavens*	6	6	5	0.40	0.33	0.40
0.67	*Pheidole flavens*	5	5	4	0.50	0.50	0.67
0.25	*Pheidole flavens*	6	6	5	0.20	0.25	0.25
0.40	*Pheidole flavens*	6	5	5	0.40	0.40	0.40
0.33	*Pheidole flavens*	5	6	4	0.33	0.33	0.33
0.60	*Pheidole flavens*	5	6	5	0.60	0.50	0.60
0.33	*Pheidole flavens*	4	6	4	0.33	0.25	0.33
0.00	*Pheidole flavens*	4	5	4	0.00	0.00	0.00
0.67	*Pheidole flavens*	5	7	4	0.67	0.50	0.67
0.50	*Pheidole flavens*	4	7	4	0.50	0.33	0.50
0.33	*Pheidole flavens*	4	6	4	0.33	0.20	0.33
0.67	*Pheidole flavens*	5	6	3	0.40	0.40	0.67
0.33	*Dolymyrmex brunneus*	9	6	6	0.29	0.25	0.33
0.33	*Dolymyrmex brunneus*	10	7	5	0.20	0.20	0.33
0.33	*Dolymyrmex brunneus*	8	4	4	0.20	0.33	0.33
0.33	*Dolymyrmex brunneus*	6	9	5	0.25	0.33	0.33
0.25	*Dolymyrmex brunneus*	10	5	5	0.25	0.25	0.25
0.33	*Dolymyrmex brunneus*	11	6	5	0.19	0.33	0.33
0.67	*Dolymyrmex brunneus*	12	8	6	0.33	0.50	0.67
0.00	*Dolymyrmex brunneus*	10	8	5	0.00	0.00	0.00
0.75	*Dolymyrmex brunneus*	10	6	5	0.43	0.60	0.75
0.00	*Dolymyrmex brunneus*	8	8	5	0.00	0.00	0.00
0.67	*Dolymyrmex brunneus*	8	6	5	0.40	0.50	0.67
0.33	*Dolymyrmex brunneus*	13	6	5	0.13	0.33	0.33
0.50	*Solenopsis substituta*	12	11	8	0.43	0.50	0.50
0.50	*Tetracha* sp.	16	15	10	0.45	0.55	0.50
0.33	*Euborellia annulipes*	10	7	6	0.33	0.33	0.33
	**Average**	7.85	6.78	5.04	0.32	0.33	0.40
	**Standard Error**	0.63	0.42	0.26	0.03	0.03	0.04

Table 4. False negative and false positive species detected by metabarcoding, Lazaro or both. In bold are the species that did not have a mitogenome deposited at the GenBank.

	**Metabarcoding**	**Lazaro**	**Both**
**False negatives**	*Pheidole obscurithorax*	***Syntermes spinosus***	*Cardiocondyla obscurior*<br>*Chrysodeixis includens*<br>*Mahanarva spectabilis*<br>*Solenopsis richteri*<br>*Spodoptera frugiperda*
**False positives**	*Euschistus heros*<br>***Gryllus argentinus***<br>*Myzus persicae*<br>***Phascolosoma esculenta***<br>***Planicephalus flavicosta***<br>*Solenopsis richteri*<br>*Spodoptera frugiperda*	*Brachymyrmex patagonicus*<br>*Pheidole obscurithorax*<br>*Solenopsis substituta*<br>*Tetracha sp.*	*Anthonomus grandis*<br>*Cardiocondyla obscurior*<br>*Doru luteipes*<br>*Dorymyrmex brunneus*<br>*Eriopis connexa*<br>*Glena unipennaria*<br>*Harmonia axyridis*<br>*Pheidole oxyops*<br>*Pheidole tristis*<br>*Selenophorus alternans*

Table 5. Coverage per individual predator and verified prey per individual in the field predator samples and false omission rate for each predator species.

Predator species	Number of samples	Individuals/ sample	Sample		Detected prey		False omission rate	
			Metabarcoding amplicons/ individual	Lazaro reads/ individual	Metabarcoding amplicons/ individual	Lazaro reads/ individual	Metabarcoding	Lazaro
Dorymyrmex brunneus	12	100	29,644	55,046	11,797	4.2	0.058	0.047
Euborellia annulipes	1	46	64,444	119,665	114	9.1	0.250	0.250
Pheidole flavens	12	200	14,822	27,523	4,867	10.2	0.044	0.058
Solenopsis substituta	1	273	10,859	20,163	317	1.3	0.286	0.167
Tetracha sp.	1	49	60,499	112,338	39,600	29.2	0.200	0.167

Table 6. Sensitivity, specificity, false discovery rate, false omission rate and accuracy for prey determinations in field collected predators by metabarcoding and Lazaro, with paired *t*-test and *p*-value.

	**Sensitivity**	**Specificity**	**False discovery rate**	**False omission rate**	**Accuracy**
Metabarcoding	0.806 (0.059)	0.577 (0.026)	0.683 (0.033)	0.073 (0.019)	0.622 (0.024)
Lazaro	0.814 (0.059)	0.618 (0.022)	0.666 (0.031)	0.068 (0.019)	0.663 (0.020)
t_{26}	0.2726	1.1514	-0.8689	-0.4596	1.3881
p-value	0.7874	0.2601	0.3929	0.6496	0.1769

Table 7. Pearson correlations between relative initial template concentration and *ln* number of read for true positives.

	r	***z*-score**	***p*-value**
**Metabarcoding**			
Harmonia axyridis	-0.306	-0.893	0.3718
Pheidole tristis	0.865	1.887	0.0592
Pheidole flavens	-0.270	-0.920	0.3577
Sp. frugiperda	0.305	0.419	0.6752
**Lazaro**			
Harmonia axyridis	0.605	1.985	0.0472
Pheidole tristis	0.962	2.027	0.0427

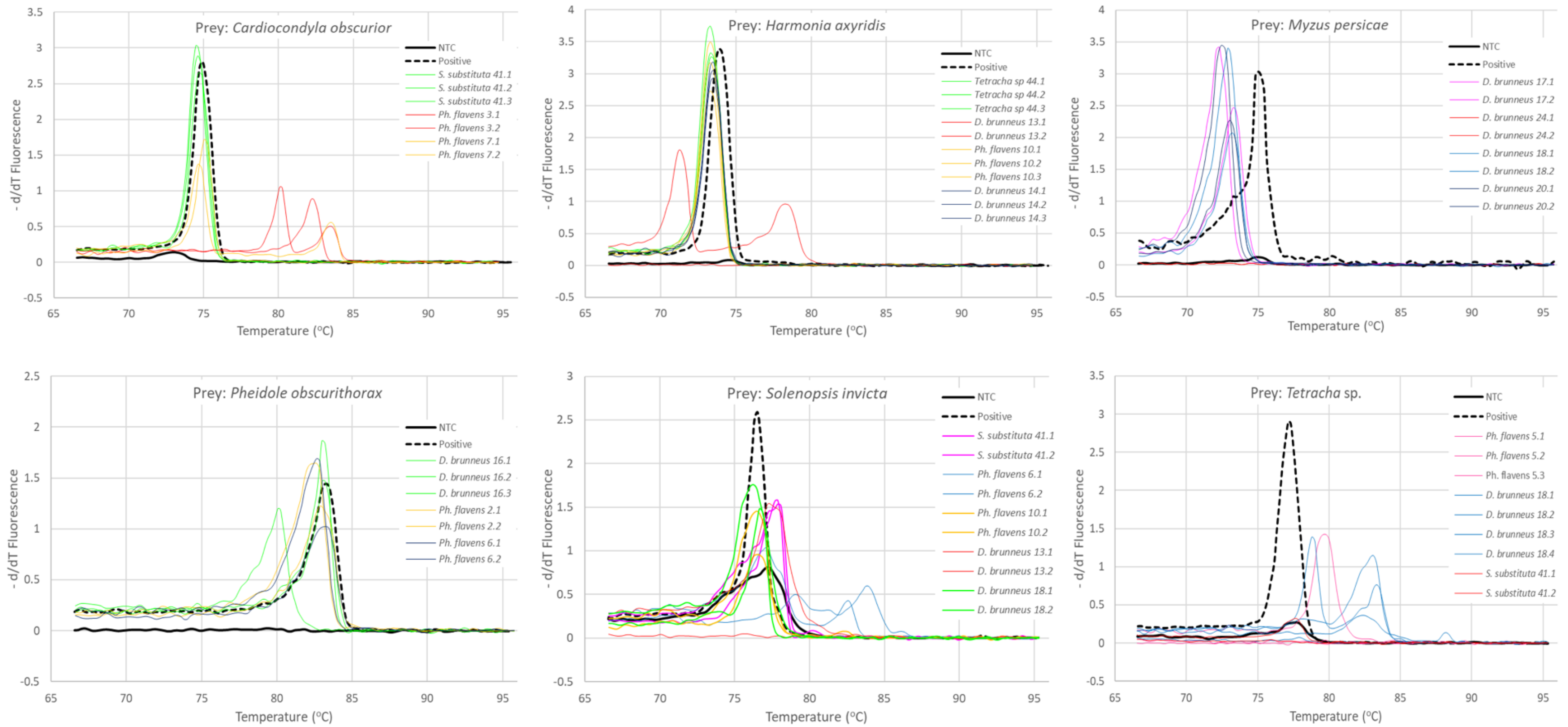

Fig. 1. Verification of prey detection by Melting Curve Analysis (MCA) in qPCR with positive controls and NTCs (no template controls). The graphs represent a melting curve for some prey detected by metabarcoding or mapping of unassembled shotgun reads (Lazaro) or both. Predator samples are informed at the side legend. Green, yellow and gray curves are positive identification of the indicated prey, and red, magenta and blue curves are negative identifications. Predator species, library identification and technical replicate number designate samples.

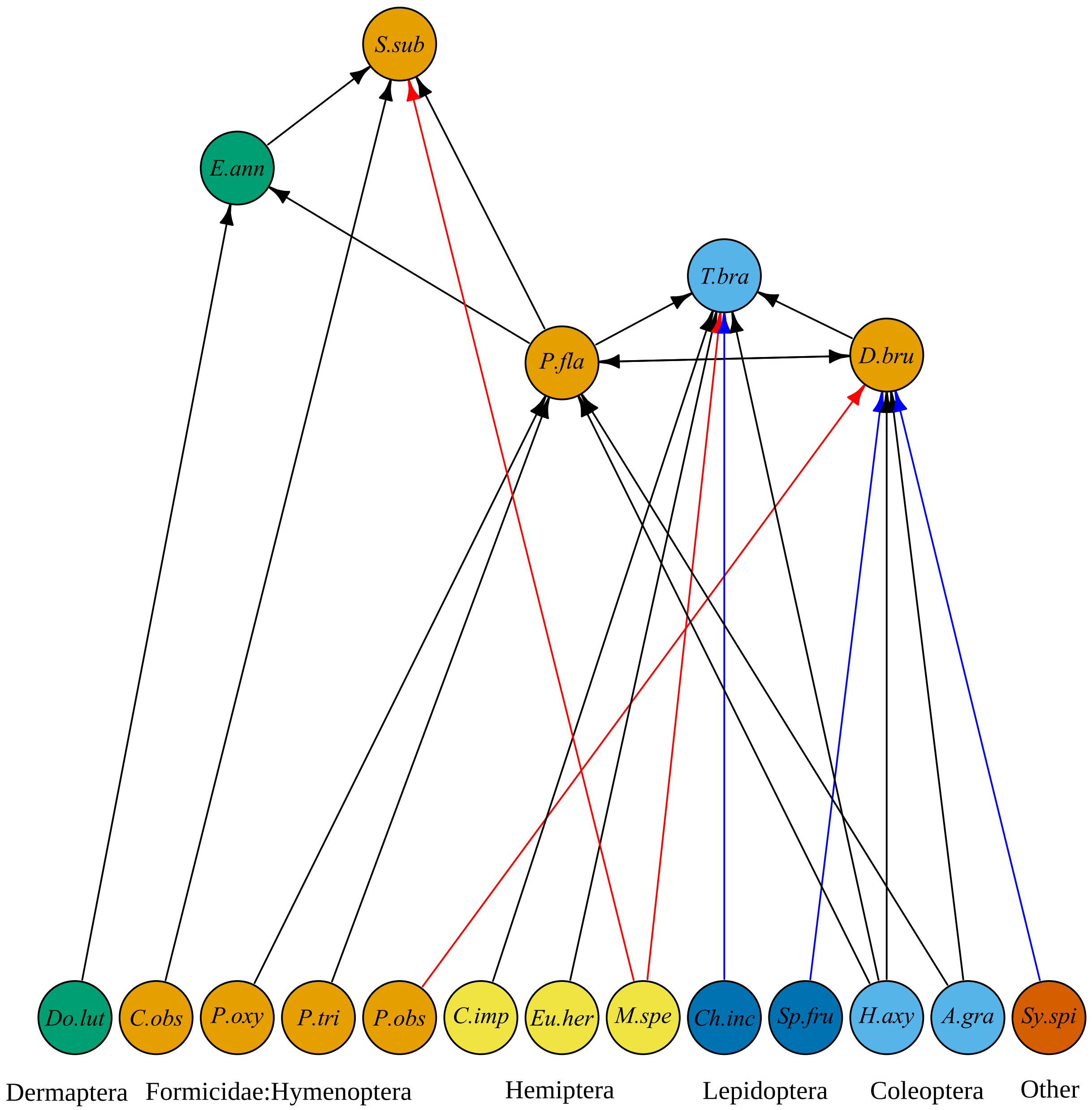

Fig. 2. Qualitative food web of the five epigeal predators (top of figure) detected by metabarcoding only (blue links), mapping of unassembled shotgun reads (Lazaro) only (red links), or both (black links) and confirmed by Melting Curve Analysis (MCA) in qPCR. Predation is indicated by the arrow direction. Height of species is the relative trophic level of the species. Predator species are *E.ann* = *Euborellia annulipes*; *S.sub* = *Solenopsis substituta*; *Ph.fla* = *Pheidole flavens*; *T.bra* = *Tetracha brasilensis*; *D.bru* = *Dorymyrmex brunneus*. Extra- or

intraguild prey are *Do.lut* = *Doru luteipes*; *C.obs* = *Cardiocondyla obscurior*; *Ph.oxy* = *Pheidole oxyops*; *Ph.tri* = *Pheidole tristis*; *Ph.obs* = *Pheidole obscurithorax*; *C.imp* = *Chinavia impicticornes*; *Eu.her* = *Euschistus heros*; *M.spe* = *Mahanarva spectabilis*; *Ch.inc* = *Chrysodeixis includens*; *Sp.fru* = *Spodoptera frugiperda*; *H.axy* = *Harmonia axyridis*; *A.gra* = *Anthonomus grandis*; *Sy.spi* = *Syntermes spinosus*.

Click here to access/download
Supplementary Material
Supporting information 1 GigaScience Dec 7 2021.docx

Supplementary Material 2

Click here to access/download

Supplementary Material

Supporting Information 2 Dec 7 2021.xlsx

Personal Cover
Click here to access/download;Personal Cover;Cover GIGA-D-21-00303 R1.docx

Dear Editor-in-Chief,

Thank you for considering our manuscript “Metabarcoding versus mapping unassembled shotgun reads for identification of prey consumed by arthropod epigeal predators” (GIGA-D-21-00303) for publication in GigaScience. We agreed with the reviewer’s suggestions.

We look forward to hearing from you and would be glad to respond to any further questions and comments.

Sincerely,

Débora
