# Supplementary material for: Metabarcoding versus mapping unassembled shotgun reads for identification of prey consumed by arthropod epigeal predators
Source: Gigascience. 2022 Mar 24;11:giac020. doi: 10.1093/gigascience/giac020 (PMC8952265; doi:10.1093/gigascience/giac020)
Supplement: giac020_Supplemental_Files [file giac020_supplemental_files.zip › Supporting information 1 GigaScience Dec 7 2021.docx]

**Supporting information 1**

**Metabarcoding versus mapping of unassembled shotgun reads for identification of prey consumed by agricultural epigeal predators**

Débora P. Paula, Suellen K. A. Barros, Rafael M. Pitta, Marliton R. Barreto, Roberto C. Togawa, David A. Andow

**Mitochondrial DNA elucidation**

Mitochondrial DNA of 29 taxa (Table S1 in Supporting Information 2) was extracted as previously described using 1 to 10 specimens, depending on the body size. In this case, whole bodies were used for total DNA extraction. An equimolar amount of DNA (1 μg/μl) of two distantly related species were pooled together and dried in a speed vacuum centrifuge. They were sent to the Roy J. Carver Biotechnology Center from the University of Illinois (USA), prepared with Hyper Libraries (Kapa Biosystems) (insert size 550 bp), and sequenced by Illumina HiSeq4000 (150 pb, paired-end) in a separate lane. The mitochondrial genome assemblies and annotations were performed based on Timbó *et al*. (2017) and deposited at GenBank (Table S1 in Supporting Information 2).

**GenBank access codes for the Sequence Read Archive (SRA) for each library**

| SRX2882443 | Raw sequence reads of the DNA gaster content of *Dorymyrmex brunneus* collected in pasture during corn season in Brazil. |
| --- | --- |
| SRX2882442 | Raw sequence reads of the DNA gaster content of *Dorymyrmex brunneus* collected in iCLF during corn season in Brazil. |
| SRX2882441 | Raw sequence reads of the DNA gaster content of *Dorymyrmex brunneus* collected off crop season in forest in Brazil. |
| SRX2882440 | Raw sequence reads of the DNA gaster content of *Dorymyrmex brunneus* collected off season in tillage in Brazil. |
| SRX2882439 | Raw sequence reads of the DNA gaster content of *Dorymyrmex brunneus* collected off crop season in pasture in Brazil. |
| SRX2882438 | Raw sequence reads of the DNA gaster content of *Dorymyrmex brunneus* collected off crop season in iCLF in Brazil. |
| SRX2882437 | Raw sequence reads of the DNA gaster content of *Dorymyrmex brunneus* collected in forest during soybean season in Brazil. |
| SRX2882436 | Raw sequence reads of the DNA gaster content of *Dorymyrmex brunneus* collected in tillage during soybean season in Brazil. |
| SRX2882435 | Raw sequence reads of the DNA gaster content of *Dorymyrmex brunneus* collected in pasture during soybean season in Brazil. |
| SRX2882434 | Raw sequence reads of the DNA gaster content of *Dorymyrmex brunneus* collected in iCLF during soybean season in Brazil. |
| SRX2882433 | Raw sequence reads of the DNA gaster content of *Dorymyrmex brunneus* collected in forest during corn season in Brazil. |
| SRX2882432 | Raw sequence reads of the DNA gaster content of *Dorymyrmex brunneus* collected in tillage during corn season in Brazil. |
| SRX2882455 | Amplicons of the mitochondrial barcode Ins16S of the gaster content of *Dorymyrmex brunneus* collected in iCLF during corn season in Brazil. |
| SRX2882454 | Amplicons of the mitochondrial barcode Ins16S of the gaster content of *Dorymyrmex brunneus* collected off crop season in forest in Brazil. |
| SRX2882453 | Amplicons of the mitochondrial barcode Ins16S of the gaster content of *Dorymyrmex brunneus* collected in tillage during corn season in Brazil. |
| SRX2882452 | Amplicons of the mitochondrial barcode Ins16S of the gaster content of *Dorymyrmex brunneus* collected in forest during corn season in Brazil. |
| SRX2882451 | Amplicons of the mitochondrial barcode Ins16S of the gaster content of *Dorymyrmex brunneus* collected off crop season in iCLF in Brazil. |
| SRX2882450 | Amplicons of the mitochondrial barcode Ins16S of the gaster content of *Dorymyrmex brunneus* collected off crop season in pasture in Brazil. |
| SRX2882449 | Amplicons of the mitochondrial barcode Ins16S of the gaster content of *Dorymyrmex brunneus* collected off season in tillage in Brazil. |
| SRX2882448 | Amplicons of the mitochondrial barcode Ins16S of the gaster content of *Dorymyrmex brunneus* collected in pasture during corn season in Brazil. |
| SRX2882447 | Amplicons of the mitochondrial barcode Ins16S of the gaster content of *Dorymyrmex brunneus* collected in iCLF during soybean season in Brazil. |
| SRX2882446 | Amplicons of the mitochondrial barcode Ins16S of the gaster content of *Dorymyrmex brunneus* collected in pasture during soybean season in Brazil. |
| SRX2882445 | Amplicons of the mitochondrial barcode Ins16S of the gaster content of *Dorymyrmex brunneus* collected in tillage during soybean season in Brazil. |
| SRX2882444 | Amplicons of the mitochondrial barcode Ins16S of the gaster content of *Dorymyrmex brunneus* collected in forest during soybean season in Brazil. |
| SRX2880493 | Raw sequence reads of the DNA gaster content of *Pheidole flavens* collected in tillage during corn season in Brazil. |
| SRX2880492 | Raw sequence reads of the DNA gaster content of *Pheidole flavens* collected in forest during corn season in Brazil. |
| SRX2880491 | Raw sequence reads of the DNA gaster content of *Pheidole flavens* collected in tillage during soybean season in Brazil. |
| SRX2880490 | Raw sequence reads of the DNA gaster content of *Pheidole flavens* collected in forest during soybean season in Brazil. |
| SRX2880489 | Raw sequence reads of the DNA gaster content of *Pheidole flavens* collected in iCLF during soybean season in Brazil. |
| SRX2880488 | Raw sequence reads of the DNA gaster content of *Pheidole flavens* collected in pasture during soybean season in Brazil. |
| SRX2880487 | Raw sequence reads of the DNA gaster content of *Pheidole flavens* collected off season in tillage in Brazil. |
| SRX2880486 | Raw sequence reads of the DNA gaster content of *Pheidole flavens* collected off crop season in forest in Brazil. |
| SRX2880485 | Raw sequence reads of the DNA gaster content of *Pheidole flavens* collected off crop season in iCLF in Brazil. |
| SRX2880484 | Raw sequence reads of the DNA gaster content of *Pheidole flavens* collected off crop season in pasture in Brazil. |
| SRX2880483 | Raw sequence reads of the DNA gaster content of *Pheidole flavens* collected in iCLF during corn season in Brazil. |
| SRX2880482 | Raw sequence reads of the DNA gaster content of *Pheidole flavens* collected in pasture during corn season in Brazil. |
| SRX2882467 | Amplicons of the mitochondrial barcode Ins16S of the gaster content of *Pheidole flavens* collected off season in tillage in Brazil. |
| SRX2882466 | Amplicons of the mitochondrial barcode Ins16S of the gaster content of *Pheidole flavens* collected off crop season in forest in Brazil. |
| SRX2882465 | Amplicons of the mitochondrial barcode Ins16S of the gaster content of *Pheidole flavens* collected off crop season in iCLF in Brazil. |
| SRX2882464 | Amplicons of the mitochondrial barcode Ins16S of the gaster content of *Pheidole flavens* collected off crop season in pasture in Brazil. |
| SRX2882463 | Amplicons of the mitochondrial barcode Ins16S of the gaster content of *Pheidole flavens* collected in tillage during soybean season in Brazil. |
| SRX2882462 | Amplicons of the mitochondrial barcode Ins16S of the gaster content of *Pheidole flavens* collected in forest during soybean season in Brazil. |
| SRX2882461 | Amplicons of the mitochondrial barcode Ins16S of the gaster content of *Pheidole flavens* collected in iCLF during soybean season in Brazil. |
| SRX2882460 | Amplicons of the mitochondrial barcode Ins16S of the gaster content of *Pheidole flavens* collected in pasture during soybean season in Brazil. |
| SRX2882459 | Amplicons of the mitochondrial barcode Ins16S of the gaster content of *Pheidole flavens* collected in tillage during corn season in Brazil. |
| SRX2882458 | Amplicons of the mitochondrial barcode Ins16S of the gaster content of *Pheidole flavens* collected in forest during corn season in Brazil. |
| SRX2882457 | Amplicons of the mitochondrial barcode Ins16S of the gaster content of *Pheidole flavens* collected in iCLF during corn season in Brazil. |
| SRX2882456 | Amplicons of the mitochondrial barcode Ins16S of the gaster content of *Pheidole flavens* collected in pasture during corn season in Brazil. |
| SRX2888397 | Raw sequence reads of the DNA gaster content of *Solenopsis substituta* collected in Brazil. |
| SRX2881298 | Amplicons of the mitochondrial barcode Ins16S of the gaster content of *Solenopsis substituta* collected during crop season in Brazil (2015). |
| SRX2888401 | Raw sequence reads of the DNA gaster content of *Euborellia annulipes* collected in Brazil. |
| SRX2882468 | Amplicons of the mitochondrial barcode Ins16S of the gaster content of *Euborellia annulipes* collected during crop season in Brazil (2015). |
| SRX2888396 | Raw sequence reads of the DNA gaster content of Tetracha sp.1 collected in Brazil. |
| SRX2882469 | Amplicons of the mitochondrial barcode Ins16S of the gaster content of *Tetracha* sp.1 collected during crop season in Brazil (2015). |

**Lazaro bioinformatics analysis**

We used the bioinformatics workflow, Lazaro, which was designed to recover the taxonomic identity of degraded environmental DNA, from the dead prey (Paula et al. 2021). Briefly, it maps reads to a reference database, identifies the mismatches (SNPs) between the query read and the reference to eliminate false mismatches and make accurate estimates of identity for a given overlap length, filters for the best matches, eliminates singletons, and filters for reads that map to coding regions. We used published and unpublished data from six experiments with known prey fed to predators and evaluated 25 thresholds, which were all combinations of overlap lengths of 100, 110, 120, 130, 140 bp, and percent identity of 96, 97, 98, 99, and 100%, to determine the optimal threshold for eliminating false positives and retaining true positives. Suitable thresholds met at least 12 of 18 criteria (three criteria in each of the six experiments), and the best threshold met the most criteria. The three criteria are: a) eliminated at least 1/3 of false positive reads while retaining at least ¾ of the true positive prey reads; b) eliminated at least 1/3 of false positive reads while retaining at least ½ of the true positive predator reads; or c) eliminated half of the false positive species while retaining 80% of the true positive prey or eliminated 2/3 of the false positive species while retaining ¾ of the true positive prey. The best threshold was 130 bp overlap with 100% identity, which met 15 of the 18 criteria. We checked the best threshold against the second best threshold (140 bp overlap with 99% identity) for removal of false positive reads and false positive species identifications. The best threshold eliminated more false positive reads and false positive species, so it was adopted for analysis of the Lazaro samples in this work.

**Cross reactivity test of species-specific primers for MCA**

To confirm that Melting Curve Analysis (MCA) could provide species-specific identifications, we examined the cross-reactivity of the primers (Table S3 in Supporting Information 2) with related species. All primers were designed to be species-specific and theoretically should have reacted only with the intended species. Amplicons with peaks that were at least 2^o^C apart were considered different.

Two species of carabids.

Cross-reactivity is shown in Figure S1. The primer pairs were species-specific, amplifying the known positive control samples, and not amplifying the other species.


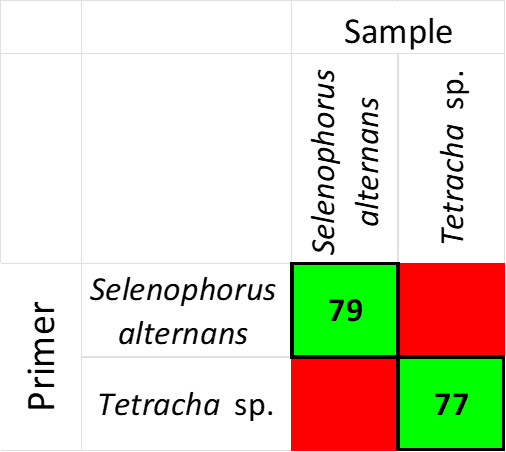


**Fig. S1.** Cross-reactivity of primers designed to detect two species of Carabidae against known positive control samples. Bright green indicates positive amplification of the positive control sample, and red indicates no amplification. Numbers are the peak of the melt temperature (^o^C).

Two congeneric species of pentatomids.

Cross-reactivity is shown in Figure S2. The *Chinavia ubica* primer reacted only with the *C. ubica* sample, while the *C. impicticornis* primer reacted with both species. Therefore, the *C. ubica* primer could be used to confirm the presence of *C. ubica* in samples, but the *C. impicticornis* primer could not. To confirm *C. impicticornis*, we tested putative positive samples with both *Chinavia* primers. Presence of *C. impicticornis* was confirmed by positive amplification by the *C. impicticornis* primers and no amplification by the *C. ubica* primers. Positive amplification by both primers would indicate presence of *C. ubica*, no amplification by both would indicate no presence of either, and no amplification by *C. impicticornis* primers and positive amplification by *C. ubica* primers would indicate presence of another species that may be related to the *Chinavia* species tested.


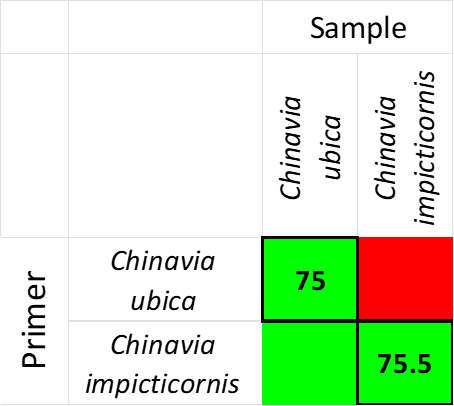


**Fig. S2.** Cross-reactivity of primers designed to detect *Chinavia* spp. against known positive control samples. Bright green indicates positive amplification of the positive control sample (inside box with black outline) or an amplicon indistinguishable from the positive control, and red indicates no amplification. Numbers are the peak of the melt temperature (^o^C).

Three species of Lepidoptera.

Although the three species of Lepidoptera in our samples were not closely related, the primers we used initially were not specific enough to allow species confirmation, so we designed additional pairs (Fig S3). We tested the species-specific qPCR primers developed for *Spodoptera frugiperda* by van de Vossenberg and van der Straten (2014), but in our case, these primers did not amplify known samples of *S. frugiperda*. Our new primers distinguished *S. frugiperda* and *C. includens*, but the *Glena unipennaria* primers reacted with all three species. Therefore, putative *G. unipennaria* samples were positive if amplified by the *G. unipennaria* primers at 80^o^C, the *S. frugiperda* primers at 77^o^C and no amplification by the *Chrysodeixis* primers. Presence of *S. frugiperda* was confirmed by positive amplification with *S. frugiperda* primers at 74^o^C. Presence of *C. includens* was confirmed by positive amplification by *C. includens* primers.


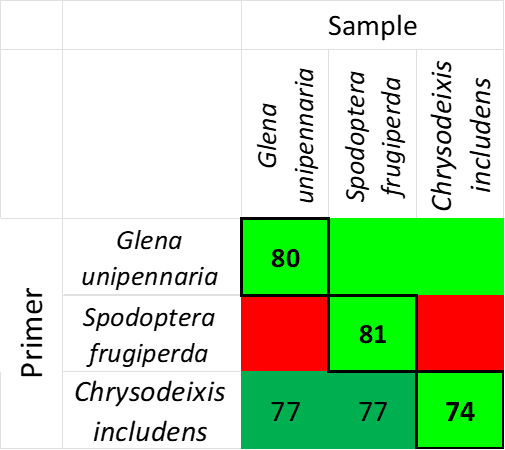


**Fig. S3.** Cross-reactivity of primers designed to detect lepidopteran species against known positive control samples. Bright green indicates positive amplification of the positive control sample (inside box with black outline) or an amplicon indistinguishable from the positive control, and red indicates no amplification. Dark green indicates an amplicon that is different from the positive control. Numbers are the peak of the melt temperature (^o^C).

Ten species of ants.

We evaluated two sets of primers to confirm the ant species. The first set did not distinguish most of the *Pheidole* and *Solenopsis* species (Fig. S4A) so additional primers were evaluated (Fig. S4B). However, the first set could confirm *Brachymyrmex patagonicus* (75^o^C), *Cardiocondyla obscurior* (76^o^C), *Dorymyrmex brunneus (*80 ^o^C) and *Pheidole flavens* (72^o^C). Confirmation of *D. brunneus* also required a negative determination for *Pheidole obscurithorax*, which could be done with any of the set 1 *Pheidole* primers or the set 2 *Pheidole tristis* primer.

*Pheidole obscurithorax*. Evaluation of putative *P. obscurithorax* in samples first involved amplification with the *P. obscurithorax* (set 1) primers. A negative determination was definitive, while samples with a positive determination required additional evaluation. These were further tested with the *D. brunneus* primers. Positive amplification indicated that the sample had *P. obscurithorax*. No amplification indicated that the sample did not have *P. obscurithorax*, but may have had another ant.

*Pheidole oxyops*. Evaluation of putative *P. oxyops* in samples first involved amplification with the *P. oxyops* (set 1) primers. A negative determination was definitive, while samples with a positive determination required additional evaluation. These were further tested with the *P. oxyops* (set 2) and *P. tristis* (set 2) primers. If both were positive, there was an undetermined ant species, but not *P. oxyops*. If the *P. oxyops* (set 2) primers amplified, but the *P. tristis* (set 2) primers did not amplify, then the presence of *P. oxyops* was confirmed in the sample. If the *P. oxyops* (set 2) primers did not amplify, the sample did not have *P. oxyops*.

*Pheidole tristis*. Evaluation of putative *P. tristis* in samples first involved amplification with the *P. tristis* (set 1) primers. A negative determination was definitive, while samples with a positive determination required additional evaluation. These samples were further evaluated with the *P. tristis* (set 2) and *Dorymyrmex brunneus* primers (Fig. S45A). If both had positive amplification, the sample had *P. obscurithorax*. If *P. tristis* (set 2) primers had positive amplification and the *D. brunneus* primers did not, the sample had *P. tristis*. If both were negative, the sample had *P. flavens*. In cases where *D. brunneus* was the predator, the determination of *P. tristis* could not be done.

*Solenopsis invicta*. Evaluation of putative *S. invicta* in samples first involved amplification with the *S. invicta* (set 1) primers. A negative determination was definitive, while samples with a positive determination required additional evaluation. None of the samples were amplified with the set 1 primers. If any samples had been amplified, we would have used the *S. richteri* (set 2) and *B. patagonicus* primers. Confirmation of *S. invicta* would have been indicated by positive amplification by both primers (*B. patagonicus* at 80.5^o^C).

*Solenopsis richteri*. Evaluation of putative *S. richteri* in samples first involved amplification with the *S. richteri* (set 1) primers. A negative determination was definitive, while samples with a positive determination required additional evaluation. These positive samples were further evaluated with the *S. richteri* (set 2) and *B. patagonicus* primers. Confirmation of *S. richteri* was indicated by positive amplification with *S. richteri* (set 2) primers and no amplification by *B. patagonicus* primers.

*Solenopsis substituta*. Evaluation of putative *S. substituta* samples first involved amplification with the *S. substituta* (set 1) primers. A negative determination was definitive, while samples with a positive determination required additional evaluation. These samples were further evaluated with the *S. substituta* (set 2) and *Brachymyrmex patagonicus* primers. Confirmation was indicated by amplification by both primers (*B. patagonicus* at 80.5^o^C).

**
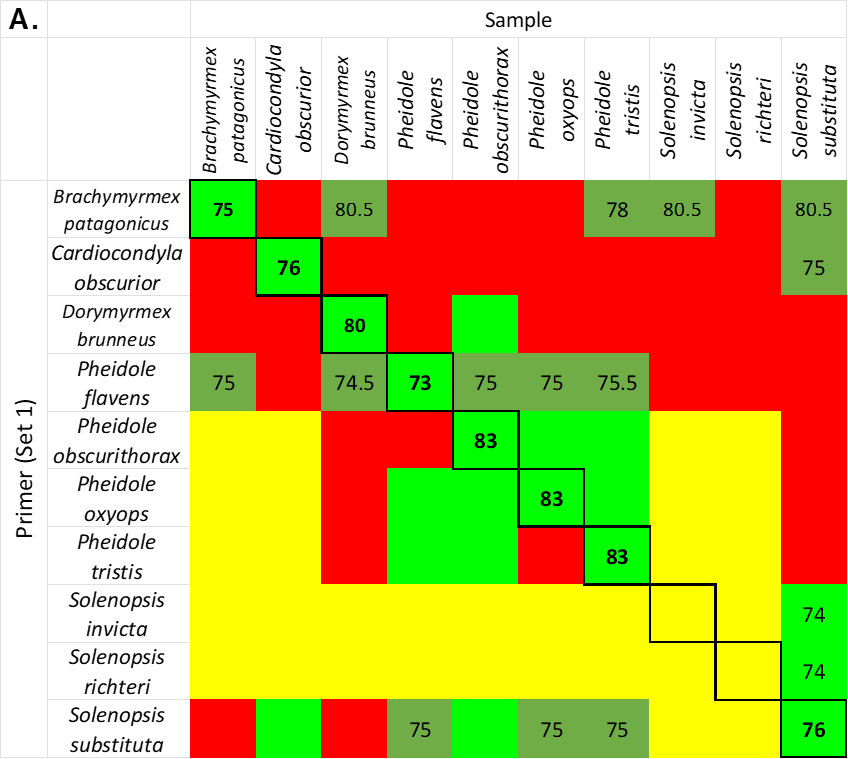
**

**
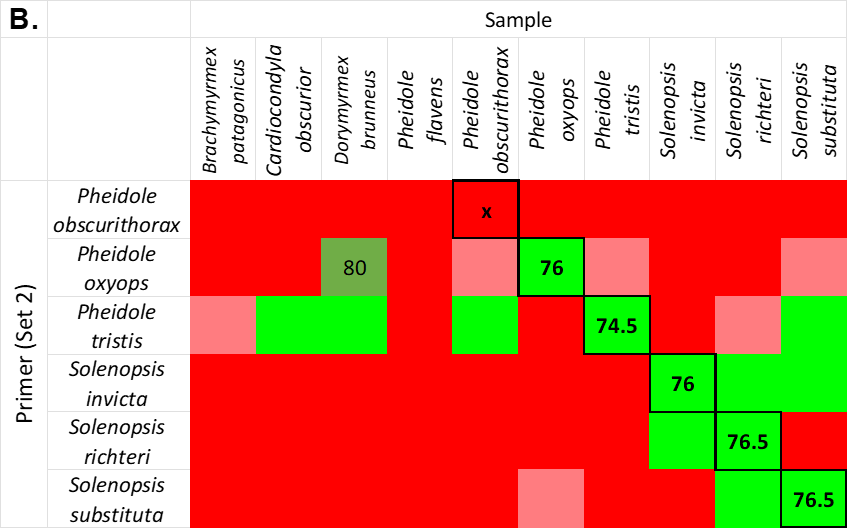
**

**Fig. S4.** Cross-reactivity of primers designed to detect ant species against known positive control samples. Bright green indicates positive amplification of the positive control sample (inside box with black outline) or an amplicon indistinguishable from the positive control, and red indicates no amplification. Dark green indicates an amplicon that is different from the positive control. Light red indicates variable responses and yellow indicates untested. Numbers are the peak melt temperature (^o^C).


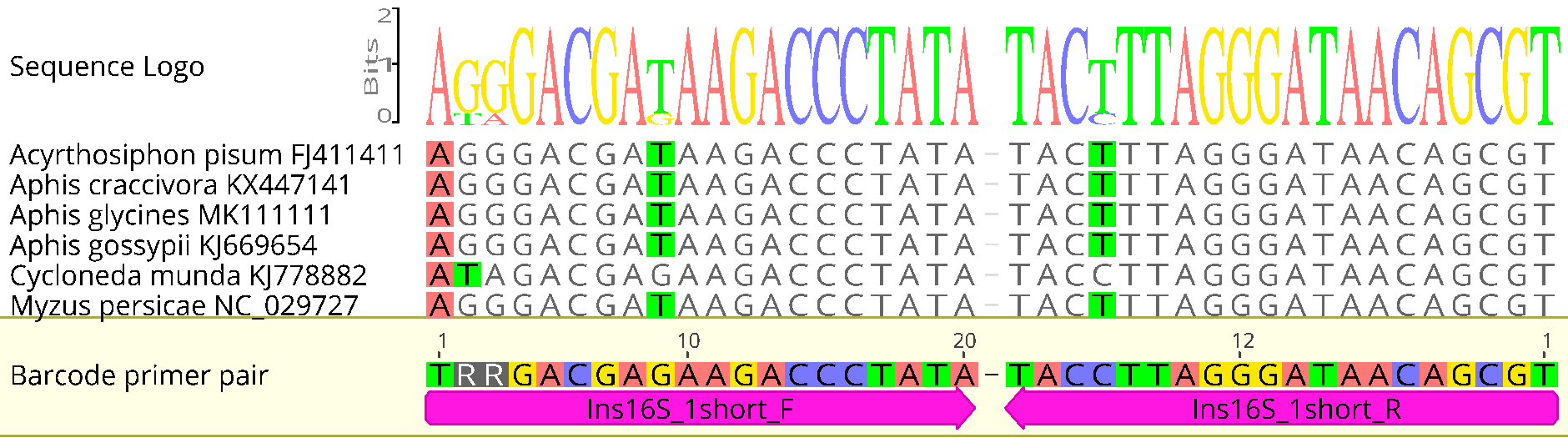


**A)**


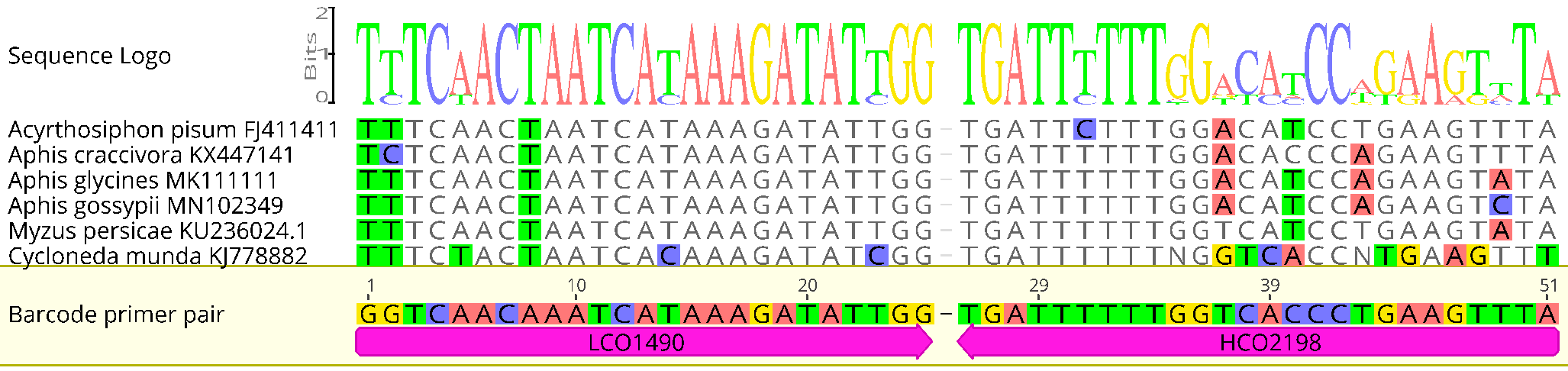


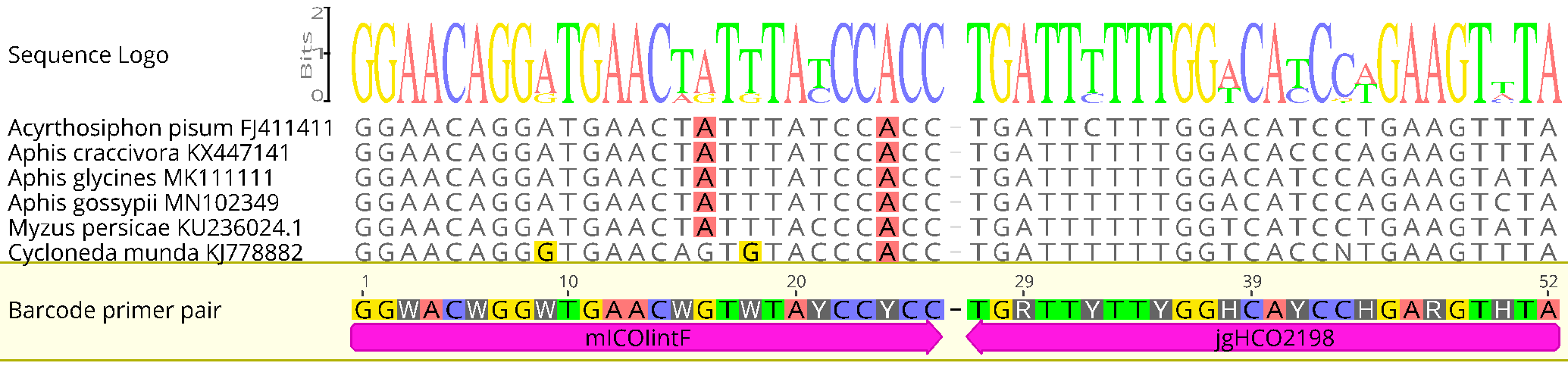


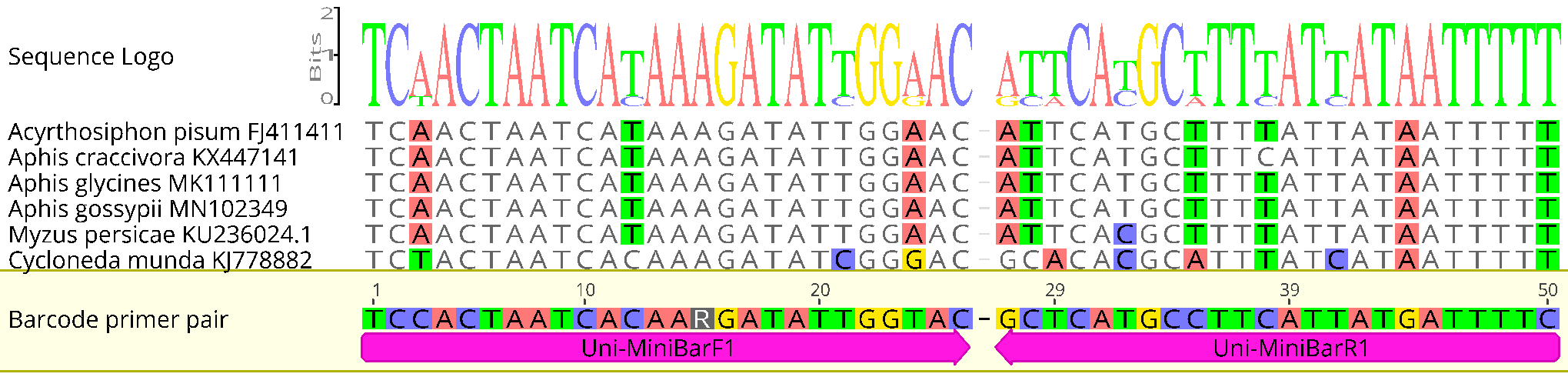


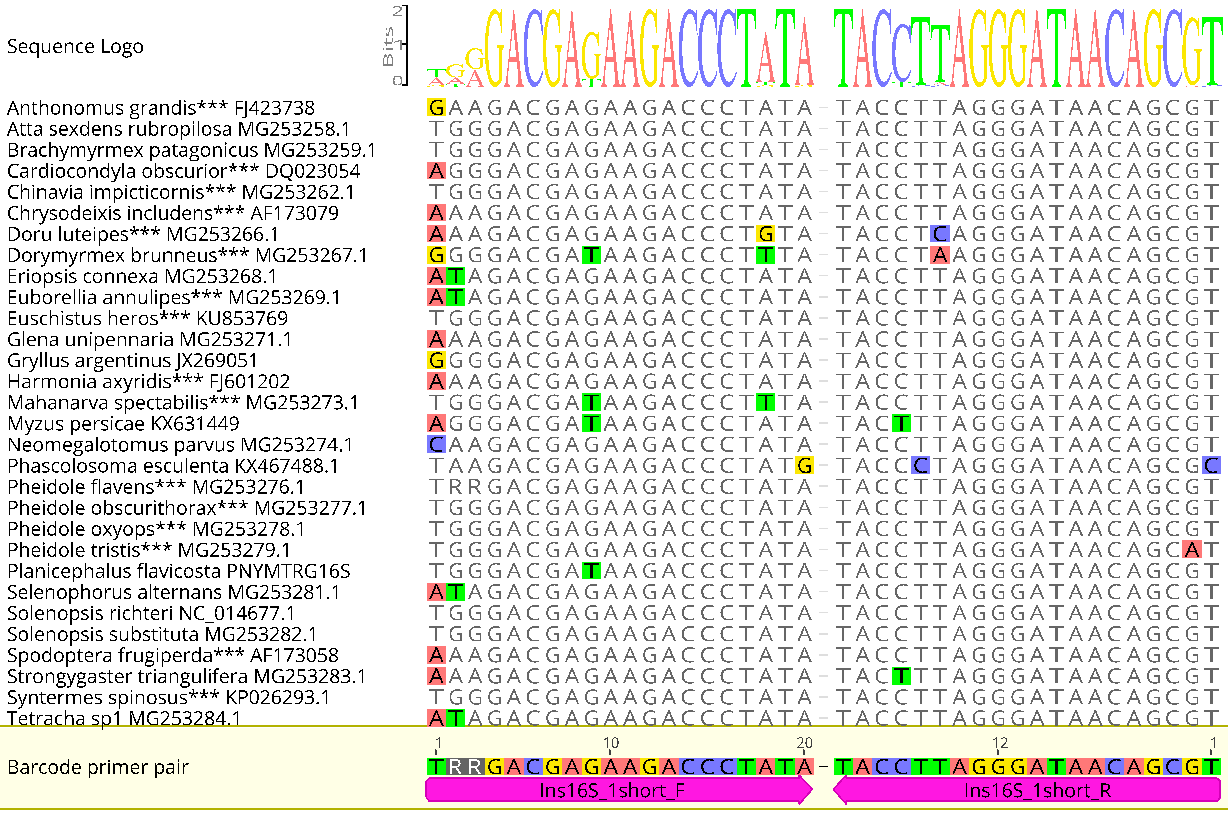

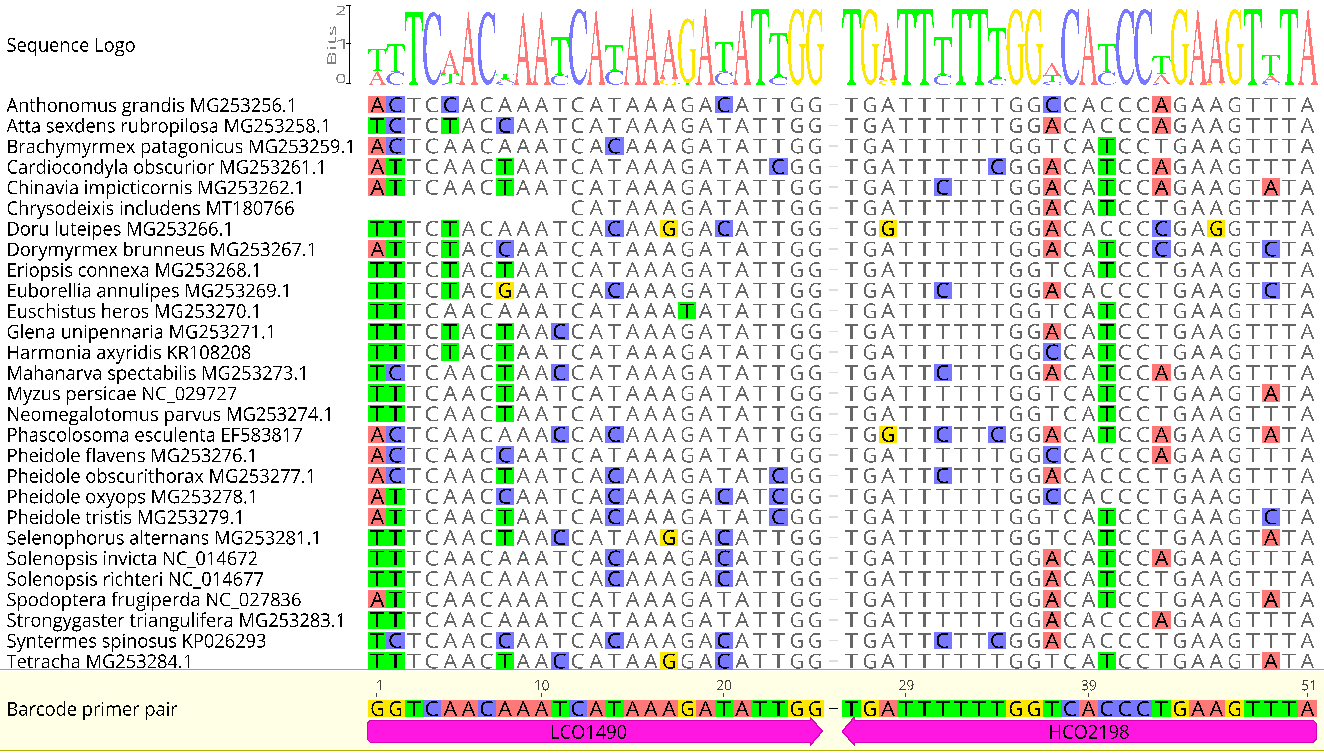


**B)**


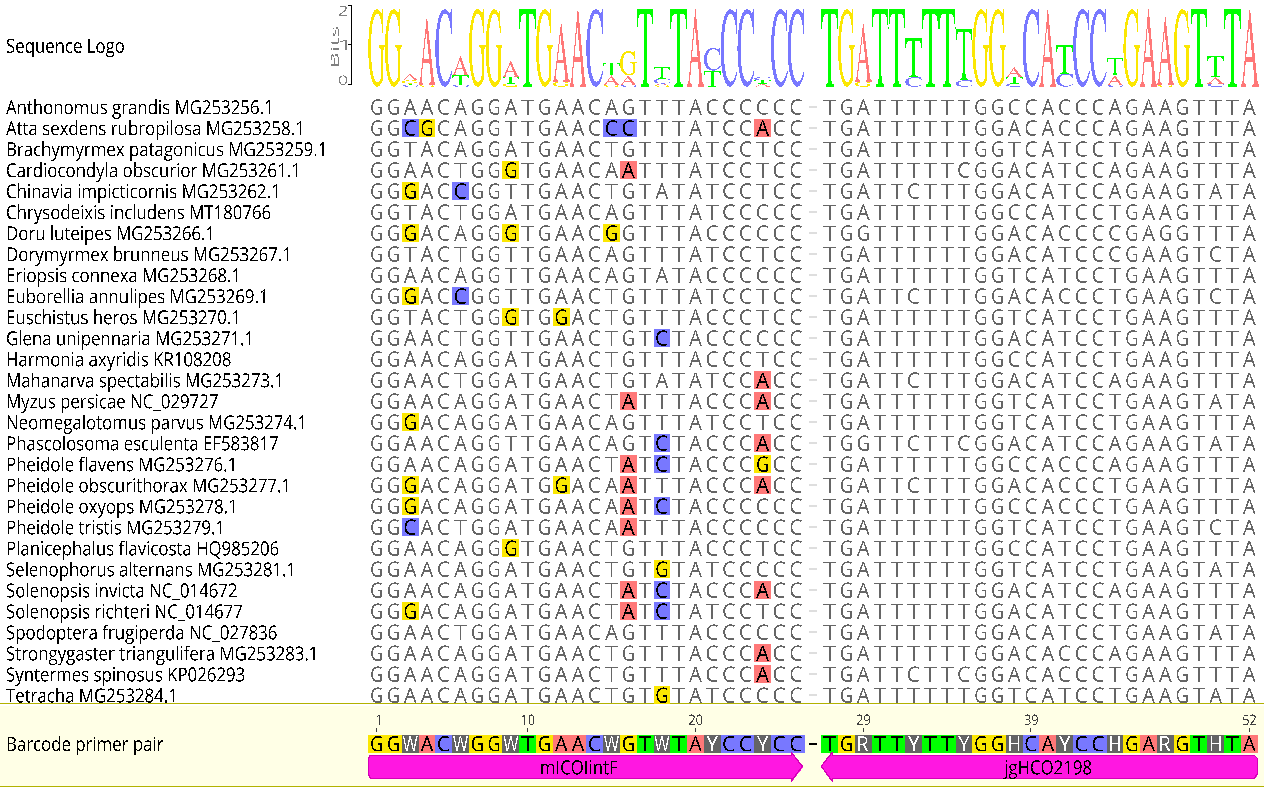

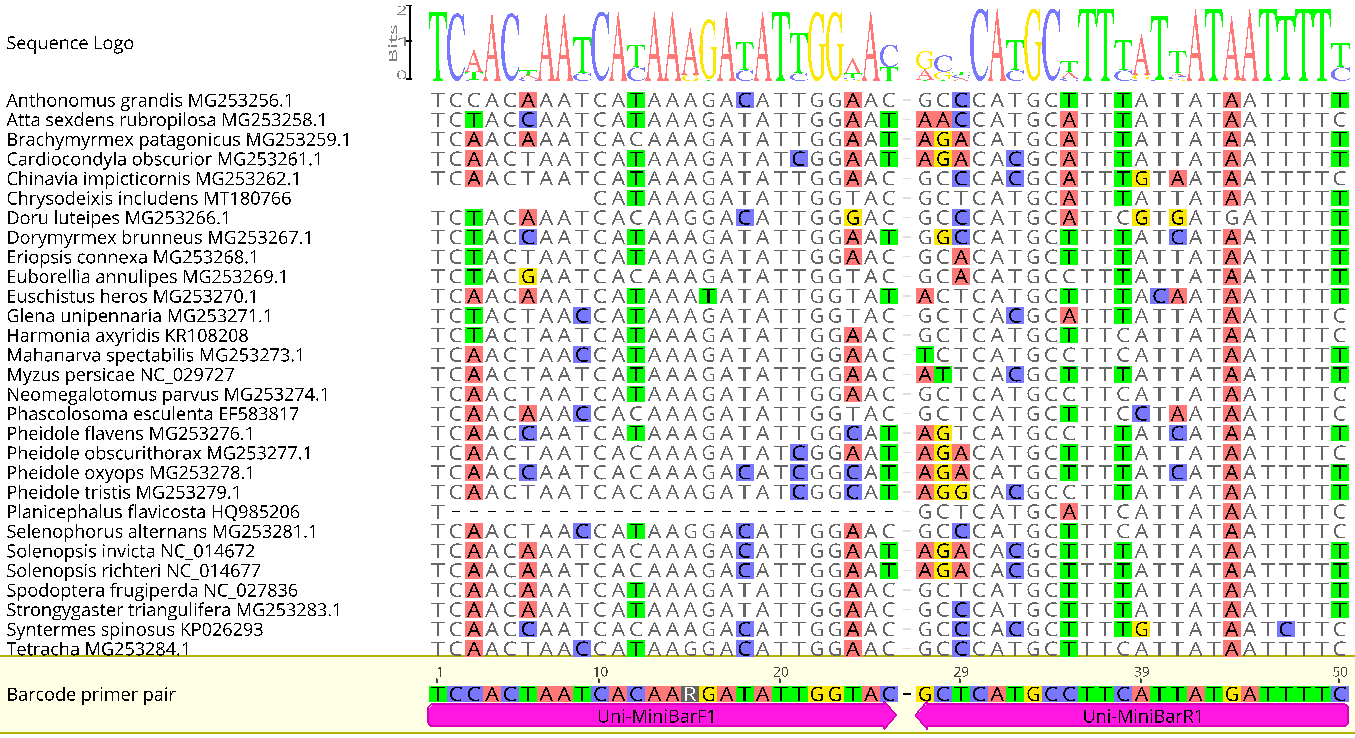


**Figure S5.** Illustration of the annealing of the primer pairs Ins16S_1short (Clarke et al. 2014), LCO1490, which we used, and HCO2198 (Folmer et al. 1994), mICOIintF and jgHCO2198 (Leray et al. 2013), and UniMini-Bar (Meusnier et al. 2008) in: A) the six prey species in the control mock community; and B) all the putative prey species detected by metabarcoding (Table 2), before MCA validation. The alignment disagreements (mismatches) between the prey and primer sequences are highlighted. W, Y, R and H are degenerate IUPAC nucleotide codes that represent: W= A or T; Y= C or T; R= A or G; H= A or C or T. The symbol ‘-’ represents the hidden nucleotide sequence between the forward and reverse primers; and the symbol ‘***’ in the 16S alignment in B indicates the true positive prey species, which were validated by MCA. The GenBank accession number of the sequences is provided after the scientific name. In B, the sequences of some of the detected species are missing (or partially represented) because we did not find their complete sequences within the predicted primer amplicon. The sequence logo above each alignment is proportional to the degree of nucleotide conservation at each position/locus.


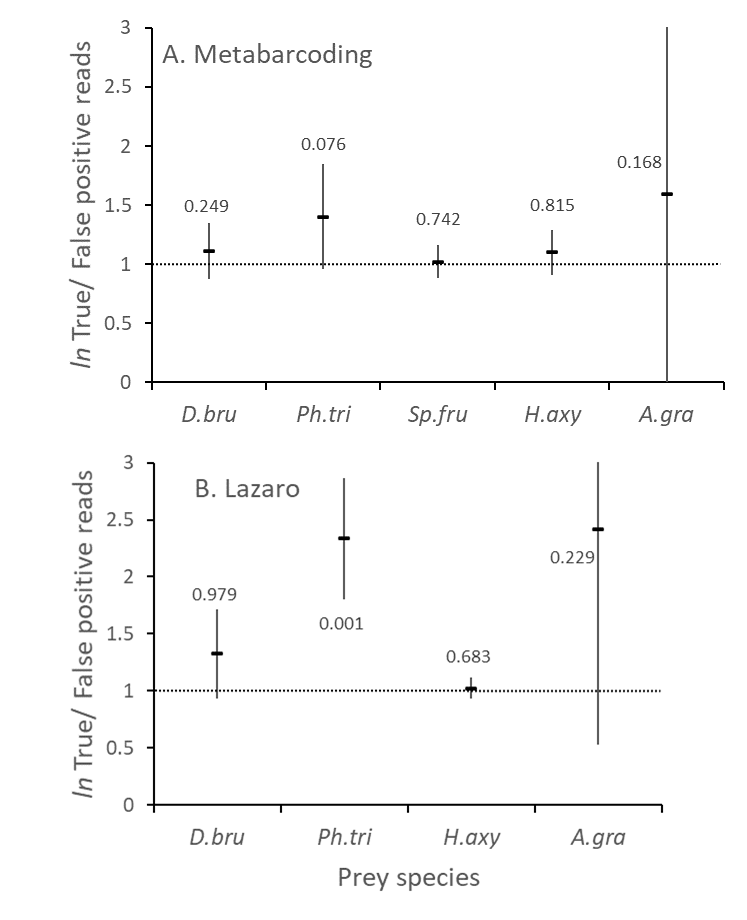


**Figure S6.** Ratio of true to false positive reads for prey species detected by A) metabarcoding and B) Lazaro, with *p*-value for Welch's *t*-test of the hypothesis that the ratio=1 and estimated 95% CIs.

--

**BLAST ® » blastn suite » results for RID-UW5YSB39016**

**Query: *Myzus persicae* metabarcode Ins16S_1 amplicon**

**Query ID: lcl|Query_62615 Length: 199**

**Database: Standard nr, nucleotide collection (nr/nt)**

**Default algorithm parameters**

**>*Macrosiphoniella kuwayamai* 16S ribosomal RNA gene, partial sequence; mitochondrial**

Taxonomy: Eukaryota; Metazoa; Ecdysozoa; Arthropoda; Hexapoda; Insecta; Pterygota; Neoptera; Paraneoptera; Hemiptera; Sternorrhyncha; Aphidomorpha; Aphidoidea; Aphididae; Macrosiphini

Sequence ID: KX631448.1 Length: 507

Range 1: 190 to 388

Score:346 bits(383), Expect:8e-91,

Identities:196/199(98%), Gaps:0/199(0%), Strand: Plus/Plus


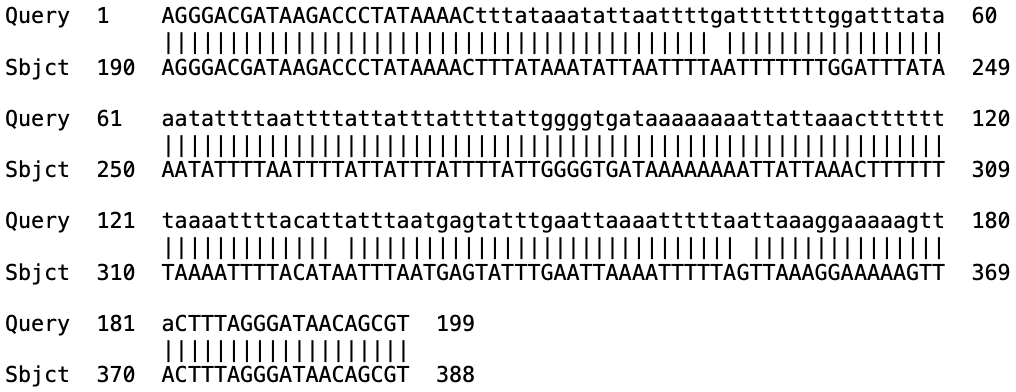


**>*Aulacorthum ibotum* voucher 030605SH04 12S ribosomal RNA and 16S ribosomal RNA genes, partial sequence; and tRNA-Val gene, complete sequence; mitochondrial**

Taxonomy: Eukaryota; Metazoa; Ecdysozoa; Arthropoda; Hexapoda; Insecta; Pterygota; Neoptera; Paraneoptera; Hemiptera; Sternorrhyncha; Aphidomorpha; Aphidoidea; Aphididae; Macrosiphini

Sequence ID: HM117795.1 Length: 1552

Range 1: 1259 to 1457

Score:346 bits(383), Expect:8e-91,

Identities:196/199(98%), Gaps:0/199(0%), Strand: Plus/Plus


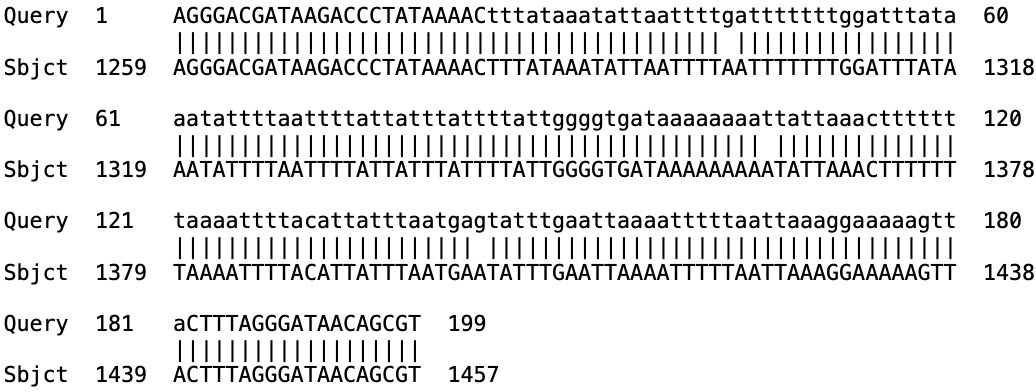


**>*Acyrthosiphon kondoi* 12S small subunit ribosomal RNA gene, partial sequence; tRNA-Val (trnV) gene, complete sequence; and 16S large subunit ribosomal RNA gene, partial sequence; mitochondrial**

Taxonomy: Eukaryota; Metazoa; Ecdysozoa; Arthropoda; Hexapoda; Insecta; Pterygota; Neoptera; Paraneoptera; Hemiptera; Sternorrhyncha; Aphidomorpha; Aphidoidea; Aphididae; Macrosiphini

Sequence ID: FJ982404.1 Length: 1582

Range 1: 1289 to 1487

Score:346 bits(383), Expect:8e-91,

Identities:196/199(98%), Gaps:0/199(0%), Strand: Plus/Plus


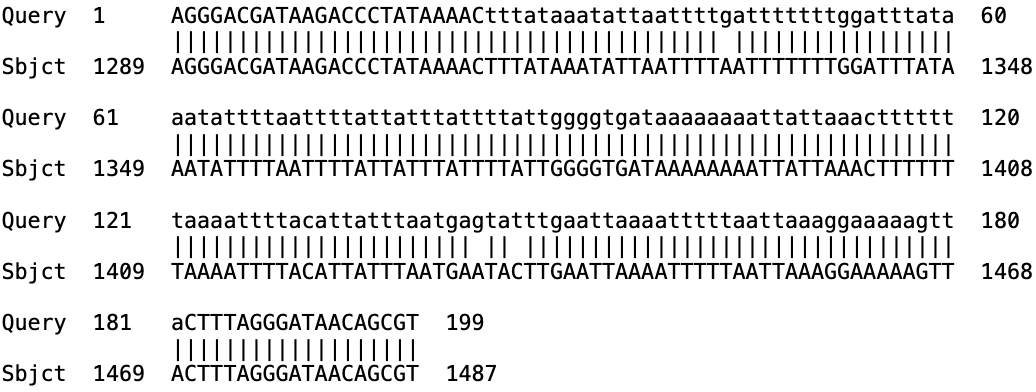


**>*Illinoia liriodendri* 16S ribosomal RNA gene, partial sequence; mitochondrial**

Taxonomy: Eukaryota; Metazoa; Ecdysozoa; Arthropoda; Hexapoda; Insecta; Pterygota; Neoptera; Paraneoptera; Hemiptera; Sternorrhyncha; Aphidomorpha; Aphidoidea; Aphididae; Macrosiphini

Sequence ID: KX631455.1 Length: 507

Range 1: 190 to 388

Score:342 bits(378), Expect:1e-89,

Identities:195/199(98%), Gaps:0/199(0%), Strand: Plus/Plus


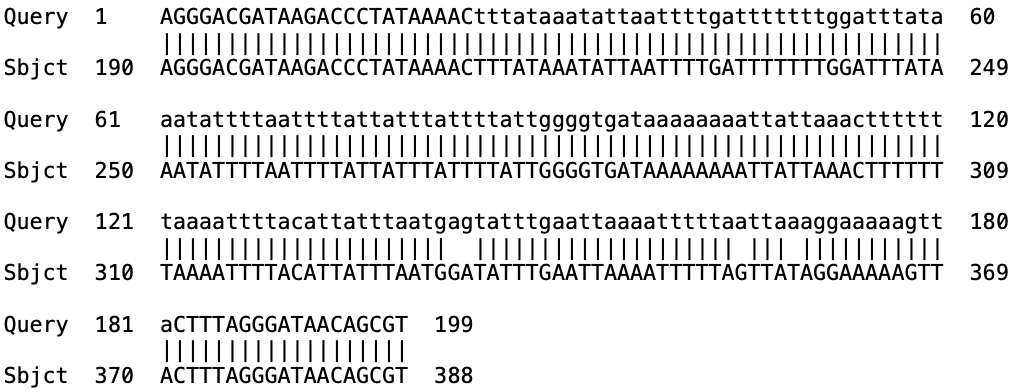


**>*Longicaudinus corydalisicola* 16S ribosomal RNA gene, partial sequence; mitochondrial**

Taxonomy: Eukaryota; Metazoa; Ecdysozoa; Arthropoda; Hexapoda; Insecta; Pterygota; Neoptera; Paraneoptera; Hemiptera; Sternorrhyncha; Aphidomorpha; Aphidoidea; Aphididae; Macrosiphini

Sequence ID: KX631450.1 Length: 507

Range 1: 190 to 388

Score:342 bits(378), Expect:1e-89,

Identities:195/199(98%), Gaps:0/199(0%), Strand: Plus/Plus


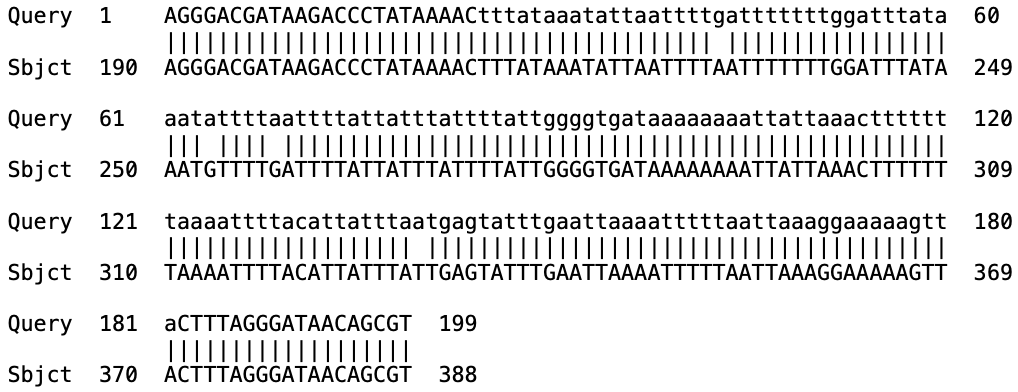


**>*Nasonovia ribisnigri* 16S ribosomal RNA gene, partial sequence; mitochondrial**

Taxonomy: Eukaryota; Metazoa; Ecdysozoa; Arthropoda; Hexapoda; Insecta; Pterygota; Neoptera; Paraneoptera; Hemiptera; Sternorrhyncha; Aphidomorpha; Aphidoidea; Aphididae; Macrosiphini

Sequence ID: KX631447.1 Length: 507

Range 1: 190 to 388

Score:342 bits(378), Expect:1e-89,

Identities:195/199(98%), Gaps:0/199(0%), Strand: Plus/Plus


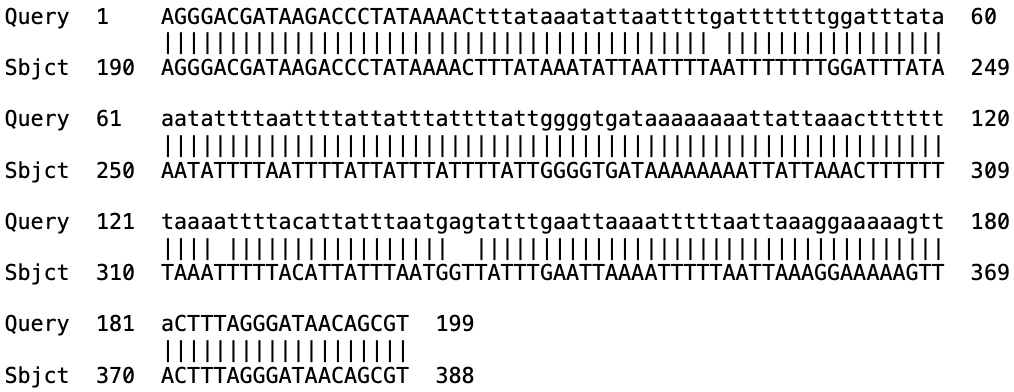


**>*Uroleucon nigrotibium* 16S ribosomal RNA gene, partial sequence; mitochondrial**

Taxonomy: Eukaryota; Metazoa; Ecdysozoa; Arthropoda; Hexapoda; Insecta; Pterygota; Neoptera; Paraneoptera; Hemiptera; Sternorrhyncha; Aphidomorpha; Aphidoidea; Aphididae; Macrosiphini

Sequence ID: KX631441.1 Length: 507

Range 1: 190 to 388

Score:342 bits(378), Expect:1e-89,

Identities:195/199(98%), Gaps:0/199(0%), Strand: Plus/Plus


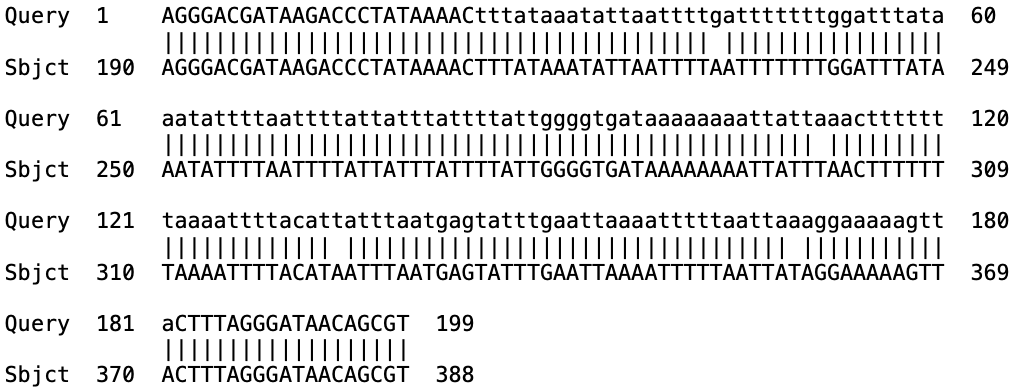


**>*Amphicercidus japonicus* voucher 030603HJ01 12S ribosomal RNA gene, partial sequence; tRNA-Val gene, complete sequence; and 16S ribosomal RNA gene, partial sequence; mitochondrial**

Taxonomy: Eukaryota; Metazoa; Ecdysozoa; Arthropoda; Hexapoda; Insecta; Pterygota; Neoptera; Paraneoptera; Hemiptera; Sternorrhyncha; Aphidomorpha; Aphidoidea; Aphididae; Macrosiphini

Sequence ID: HM117797.1 Length: 1546

Range 1: 1253 to 1451

Score:342 bits(378), Expect:1e-89,

Identities:195/199(98%), Gaps:0/199(0%), Strand: Plus/Plus


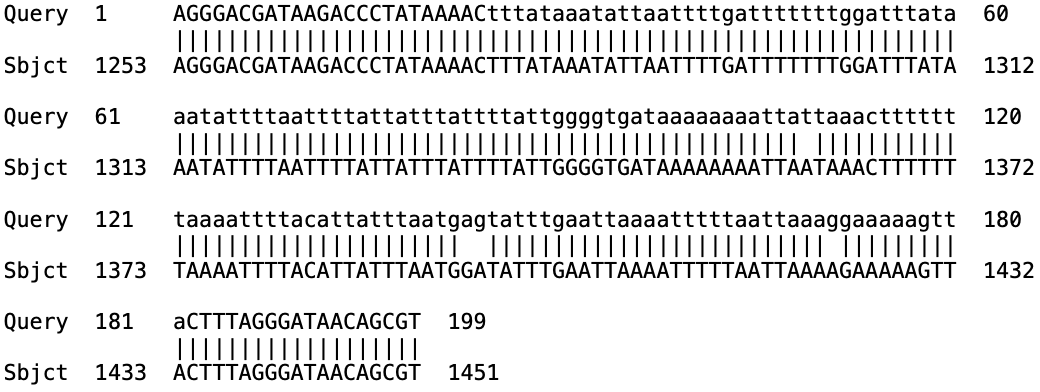


**>*Megoura brevipilosa* isolate 030523SH33 12S small subunit ribosomal RNA gene, partial sequence; tRNA-Val gene, complete sequence; and 16S large subunit ribosomal RNA gene, partial sequence; mitochondrial**

Taxonomy: Eukaryota; Metazoa; Ecdysozoa; Arthropoda; Hexapoda; Insecta; Pterygota; Neoptera; Paraneoptera; Hemiptera; Sternorrhyncha; Aphidomorpha; Aphidoidea; Aphididae; Macrosiphini

Sequence ID: EU071316.1 Length: 1586

Range 1: 1286 to 1484

Score:342 bits(378), Expect:1e-89,

Identities:195/199(98%), Gaps:0/199(0%), Strand: Plus/Plus


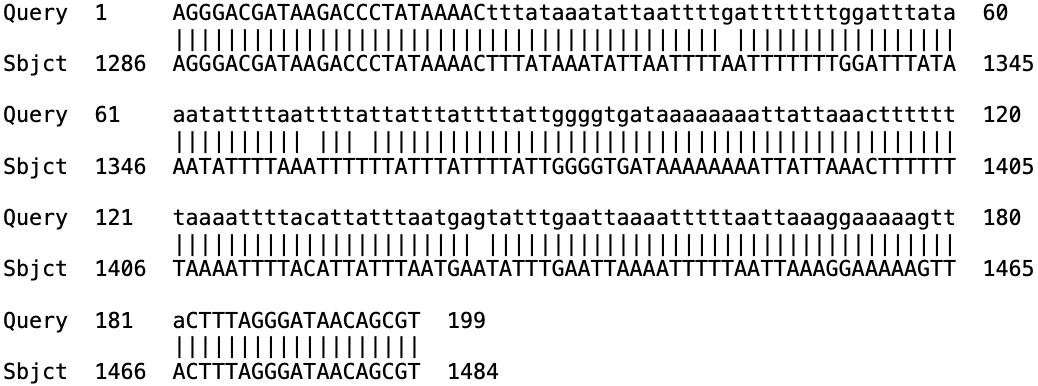


**>*Aulacorthum nipponicum* 12S small subunit ribosomal RNA gene, partial sequence; tRNA-Val (trnV) gene, complete sequence; and 16S large subunit ribosomal RNA gene, partial sequence; mitochondrial**

Taxonomy: Eukaryota; Metazoa; Ecdysozoa; Arthropoda; Hexapoda; Insecta; Pterygota; Neoptera; Paraneoptera; Hemiptera; Sternorrhyncha; Aphidomorpha; Aphidoidea; Aphididae; Macrosiphini

Sequence ID: FJ982410.1 Length: 1581

Range 1: 1287 to 1485

Score:342 bits(378), Expect:1e-89,

Identities:195/199(98%), Gaps:0/199(0%), Strand: Plus/Plus


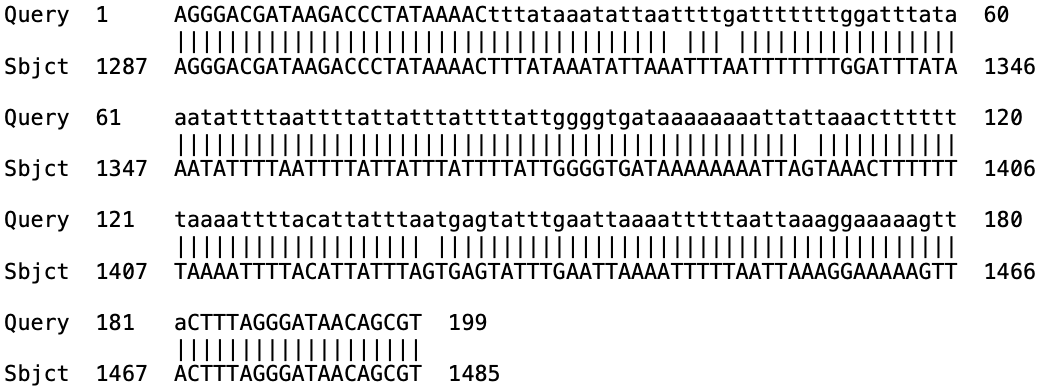


**>*Aulacorthum magnoliae* 12S small subunit ribosomal RNA gene, partial sequence; tRNA-Val (trnV) gene, complete sequence; and 16S large subunit ribosomal RNA gene, partial sequence; mitochondrial**

Taxonomy: Eukaryota; Metazoa; Ecdysozoa; Arthropoda; Hexapoda; Insecta; Pterygota; Neoptera; Paraneoptera; Hemiptera; Sternorrhyncha; Aphidomorpha; Aphidoidea; Aphididae; Macrosiphini

Sequence ID: FJ982408.1 Length: 1581

Range 1: 1288 to 1486

Score:346 bits(383), Expect:8e-91,

Identities:196/199(98%), Gaps:0/199(0%), Strand: Plus/Plus


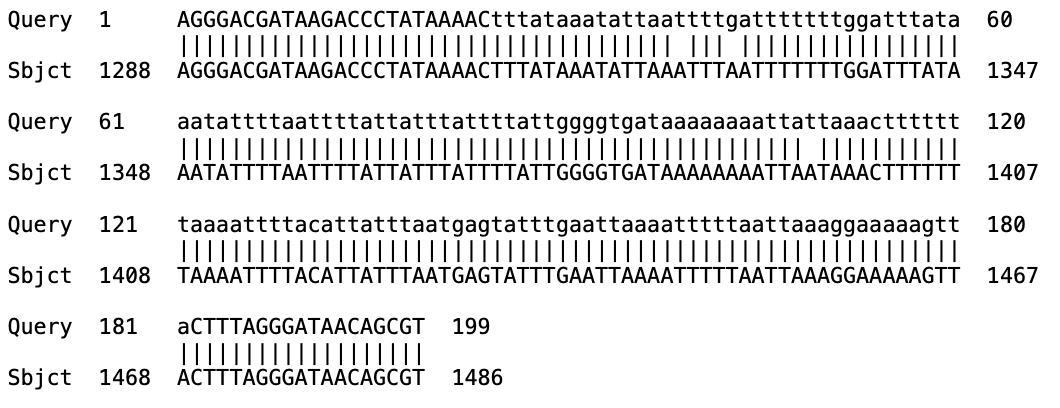


**References**

Clarke LJ, Soubrier J, Weyrich LS, Cooper A. Environmental metabarcodes for insects: *in silico* PCR reveals potential for taxonomic bias. Mol Ecol Resour. 2014;14:1160-1170.

Folmer O, Black M, Hoeh W, Lutz R, Vrijenhoek R. DNA primers for amplification of mitochondrial cytochrome c oxidase subunit I from diverse metazoan invertebrates. Mol Mar Biol Biotechnol. 1994;3(5):294-9.

Leray M, Yang JY, Meyer CP et al. A new versatile primer set targeting a short fragment of the mitochondrial COI region for metabarcoding metazoan diversity: application for characterizing coral reef fish gut contents. Front Zool. 2013;10:34.

Meusnier I, Singer GAC, Landry JF, Hickey DA, Hebert PDN, Hajibabaei M: A universal DNA mini-barcode for biodiversity analysis. BMC Genomics 2008;9:214.

Paula DP, Timbó RV, Togawa RC, Vogler AP, Andow DA Quantitative prey species detection in predator guts across multiple trophic levels by DNA shotgun sequencing. 2021. <https://doi.org/10.1101/2021.04.01.438119>.

Timbó RV, Togawa RC, Costa MMC, Paula DP Mitogenome sequence accuracy using different elucidation methods. PLoS ONE 2017;12(6):e0179971.

Van de Vossenberg BT, Van der Straten MJ. Development and validation of real-time PCR tests for the identification of four *Spodoptera* species: *Spodoptera eridania*, *Spodoptera frugiperda*, *Spodoptera littoralis*, and *Spodoptera litura* (Lepidoptera: Noctuidae). J Econ Entomol. 2014;107(4):1643-54.
