# Supplementary material for: Metabarcoding versus mapping unassembled shotgun reads for identification of prey consumed by arthropod epigeal predators
Source: Gigascience. 2022 Mar 24;11:giac020. doi: 10.1093/gigascience/giac020 (PMC8952265; doi:10.1093/gigascience/giac020)
Supplement: giac020_Response_to_Reviewer_Comments_Revision_1 [file giac020_response_to_reviewer_comments_revision_1.pdf]

Author's Response To Reviewer Comments

Close

Dear Editor-in-Chief,
Thank you for considering our manuscript "Metabarcoding versus mapping unassembled shotgun reads for identification of prey consumed by arthropod epigeal predators" (GIGA-D-21-00303) for publication in GigaScience. Please find below our point-by-point response to the reviewer comments. We agreed with their suggestions.
We look forward to hearing from you and would be glad to respond to any further questions and comments.
Sincerely,
Debora
--

Reviewer reports:

Reviewer #1: Dear Dr. Paula
The manuscript has been improved considerably from the initial version. The introduction in now an enjoyable read with enough background on competing methodologies. The rest of the manuscript now offers sufficient detail to objectively assess the study and its results. The responses to reviewer queries were throughout and satisfactory. The supplementary materials are informative as well.
I still feel the methods comparison is somewhat unfair to metabarcoding, performance of which can be improved in several ways: e.g. processing individuals separately, while still sequencing them in a multiplexed single library; improving or adding primers; using blocking primers or optimising PCR conditions (40 cycles will definitely mask rare prey species due to the exponential nature of amplification). Most of these aspects are of course discussed, and attention is drawn to them by the a priori hypotheses not being confirmed.
>>>Response:
a) The individuals were processed equally for both methods as the total DNA extracted was split among the 3 molecular methods (metabarcoding, Lazaro and MCA-qPCR) to avoid any bias regarding sample processing. In fact, we suppressed the tagging step to avoid tag jumping source of errors (false positives), ultimately enabling metabarcoding to perform more accurately than usual.
b) The argument that using additional primers would improve detection of true positive prey could also be used to suggest an increased detection of false positive prey. Although there are a number of publications that demonstrate that the use of multiple primers increase the diversity of prey detection, they often don't present a concomitant analysis of the effect on false prey detection. Moreover, there is no guarantee that the use of multiple primers will always improve metabarcoding performance. To illustrate this point we added additional illustrations in the Fig. S5 (Supporting information 1) to show the potential mismatches of the 16S barcode primer pair that we used and of 3 commonly used COI barcode primer pairs (LCO1490 and HCO2198, amplicon length ca. 710 bp, Folmer et al. 1994; mICOIintF and jgHCO2198, amplicon length ca. 313 bp, Leray et al. 2013; UniMiniBar, amplicon length 100-150 bp, Meusnier et al. 2008) with the sequences of the prey species used in the control mock community assay and detected in the field gut content samples. As it can be seen, the COI primer pairs LCO1490/HCO2198 and UniMiniBar had many more mismatches than our 16S primer pair, and therefore would likely have detected fewer prey species than the 16S primer pair. The COI primer pair mICOIintF and jgHCO2198 has high degeneracy, and could have performed equivalently or a little better than the 16S primer pair, but the high degenerance comes with the price of lower primer amplification efficiency. In addition, the forward primer (mICOIintF) seems to form a mismatch in the critical last three bases of the 3'-end with all 6 species used in the control mock community assay and in 8 out of 29 species (27%) detected in the field samples. Regarding research to improve a metabarcode primer pair, this has not been not easy, and several studies have been published on the search for better metabarcodes, but due to many factors (e.g.,taxonomic coverage; sample nature complexity) there have been no breakthroughs. So, we used one of the barcode primer pairs available nowadays to make the comparison between the two detection methods.
c) The use of blocking primers is not recommended for gut content analysis of generalist predators because the blocking primers block the detection of species closely related to the predator. In

biodiversity surveys, it might be useful to use blocking primers, yet one could still face problems (Pinol et al. 2014, doi: 10.1111/1755-0998.12156). The PCR conditions used in this study were optimised previous to the sample analysis, including the number of cycles to be used.

Nevertheless, the article is helpful to anyone aiming to either further develop methods for molecular analysis of trophic interactions or to choose an existing protocol for ecological studies. The melting curve analysis elevates the study to a level required by the journal.
Thus, I only have few very small comments (line numbers refer to the word document).

Line 84: I know this was suggested by reviewer 1. but a "unanimous consensus" is a tautology
>>>Response: Modified accordingly.

Line 98: should be: one adult aptera
>>>Response: Modified accordingly.

Line 338: should be: Neither metabarcoding or Lazaro produced any false positives
>>>Response: Modified accordingly.

Line 478: I would say higher detection of false positives instead of lower in this sentence to be consistent with the hypothesis and the previous two sentences. "this did not convert into a higher detection of true positive or a lower detection of false positive prey detections compared to Lazaro."
>>>Response: Modified accordingly.

The reference list needs some touches to match journal guidelines. E.g. doi format, italisation of journal names.
>>>Response: References were revised and meet the journal guidelines .

Yours, Dr. Tuomas Kankaanpää, Ecology and Genetics unit, University of Oulu.

Reviewer #2: The effort taken by authors to address the comments from the previous round of review is significant and admirable. The authors have clearly taken great care and effort to strengthen the manuscript, which has evidently led to some excellent additions. The experimental controls and more detail around the overall QC are very welcome additions. The additional information regarding the number of individuals per sample and sequencing depth are very valuable too. The differentiation in nomenclature between "DDSS" and "Lazaro" is a good idea (and the new name is great)!
Regarding the response to the comment about data cleanup, appreciably "post-bioinformatic" is quite vague and disregards any processes incorporated into the bioinformatics. The additional information and controls presented in the revised manuscript are an excellent addition. Many metabarcoding studies apply quality thresholds (sometimes arbitrary, e.g., remove taxa with <100 reads, sometimes more uniform and robust, e.g., remove any reads that comprise less than 1 % of the sample read count) to remove false positives that arise through contamination/error. Whilst the scale of these errors might be reduced in Lazaro (i.e., no PCR amplification of false positives), there are still many opportunities through which they may arise (e.g., environmental/lab contaminants, sequencing errors) so it would be interesting to know how these might best be tackled going forward. Given the novelty of these techniques, it is appreciably difficult to determine best practice, so it is great to see that this is now more overtly incorporated.

In the response to reviewers, it is mentioned that controls will not identify contamination that occurs after DNA extraction, but that is not always the case. A distinction between negative controls (e.g., extractions without input tissue or PCRs without input DNA) and blanks (e.g., unused combinations of used sample tags). Equally, running samples in triplicate (as is done for some aspects of this manuscript) can facilitate identification of post-extraction contamination. Of course, these are just suggestions for further applications and would be an unfair expectation of every such study given the financial upscaling associated. Whilst the authors are correct in suggesting that this type of contamination may occur infrequently and thus contamination may not be detected by these controls, the statement that this means the controls give false confidence in the lack of contamination should be revisited. Surely not screening for this contamination at all is much worse in this regard than checking and not discovering any contamination.
>>>Response: We agree that not checking at all for contamination is a bad idea, and the reviewer has made some suggestions that could be examined in the future. Our concern was with infrequent contamination of a few samples after DNA extraction. As there are many steps between DNA extraction

and sequencing, the best approach for detecting contamination is still not clear. The idea of using unused combinations of the sample tags as blanks would check if contaminants were entering via the tags or if many samples were being contaminated during the PCR step. The idea of maintaining the triplicate PCR products separate, would help detect contamination during the PCR step, however it would increase the likelihood of missing the rare DNA. Contamination could occur at many more steps of the process, so this problem merits serious investigation that goes beyond the scope of the present study (as recognized by the reviewer).

Regarding cannibalism, is there no potential to identify instances of cannibalism in future studies by looking for intraspecific genetic variation? Obviously this may be beyond the scope of this study, but it would certainly be a great benefit over metabarcoding if it could be achieved. Even if only by incorporation of phylogenetic distances between individual predators in the analysis, there seems to be great potential for lateral streams of data using this approach which appear to be otherwise untapped.
>>>Response: In another study, we are precisely testing this hypothesis if it is possible to distinguish different specimens by intraspecific genetic variation. But as the reviewer said, this is beyond the scope of this study.

Some specific comments:
Lines 19-20: Here it is stated that metabarcoding did not detect any prey and that Lazaro detected 43-71 %. Without stating the possible reasons for the poor detection of prey by metabarcoding, this seems to be an unfair representation, since it seems to come down to primer choice. Positive results from ecoPCR for amplification of the target species is mentioned later as a justification for it not being a primer problem, but importantly ecoPCR is a very simple simulation of PCR, neglecting critical aspects of primer success such as 3' proximity and adjacency of mismatches. Ultimately, it seems that primer mismatch or preferential amplification of predator DNA were likely causes of this issue, which is of course an indictment of metabarcoding more generally anyway (i.e., primer mismatches are unpredictable and can hamper taxonomic coverage sporadically), but it could obviously have been circumvented by alternative primer choice, or by using multiple primer pairs. Ultimately though, to test whether this primer mismatch issue is the cause of this issue, one could simply diagnostically test tissue extracts from these taxa with the primers in question.
>>>Response: We added a statement in line 20 to express that one possibility why metabarcoding did not detect any prey from the mock community could be related to our primer choice and or preferential predator DNA amplification. However, ecoPCR has a feature to avoid adjacent mismatches in the 3' proximity, as informed in the example 2 of the software documentation (https://pythonhosted.org/OBITools/scripts/ecoPCR.html): "a perfect match can be enforced by adding a '#' after the considered nucleotide. Example 2:
> ecoPCR -d mydatabase -e 2 -l 80 -L 120 -D 50 -r 7742 \
TTAGATACCCCACTATG#C# TAGAACAGGCTCCTCTA#G# > mysequences.ecopcr". We used this # feature to avoid adjacent mismatches in the 3' proximity. Additionally, one should also consider that the use of multiple barcode primers can simultaneously increase the rate of false positive detection, so if the use of another primer could have resulted in true prey detection, it could also have resulted in increased false prey detection. In addition, we only used one reference database for Lazaro's analysis as well, when in fact we could have used multiple reference databases to improve prey detection by Lazaro. We added these to the main text.

Line 27: It is stated that Lazaro and metabarcoding are "similar in these respects" and then two percentages are given, but what do these refer to? Detection of true positives, false positives and false negatives, and taxonomic resolution are all mentioned, so it is not clear which these percentages pertain to.
>>>Response: Modified accordingly.

Lines 28: The statement that metabarcoding detected 350X more true prey reads is surely uninformative because those reads are amplified, so what does this tell us? It is then stated that this doesn't convert into a higher number of true positive prey detections, which is to be expected. It is probably a valuable statement for the manuscript, but it doesn't feel important enough for the abstract when there are so many great findings already there.
>>>Response: Modified accordingly.

Line 34: I feel that the "may" and "can" should be flipped. Whilst there is some debate around quantification of metabarcoding reads, there seems to be a general consensus that even the best attempts require additional experimental data (i.e., it cannot be quantitative in isolation). Lazaro,

however, shows promise for quantification, but without more studies and data from alternative applications, this seems speculative still (thus "may" could be better).
>>>Response: Modified accordingly.

Line 57: "do not assemble"
>>>Response: Modified accordingly.

Line 57: "prior" may be more appropriate than "previously"
>>>Response: Modified accordingly.

Line 80: This is the first main text mention of Lazaro; it would be valuable to introduce it. It is introduced on line 88; perhaps these could be switched. It might be nice to include briefly the justification for the name too.
>>>Response: Modified accordingly.

Lines 96-103: Was it visually confirmed that all seven prey were consumed by the predators?
>>>Response: Yes, it was visually confirmed. Now we mention that in the main text.

Lines 286-307: It's unconventional to present hypotheses at the end of the methods. It would seem more logical to include them at the end of the introduction. This is not, however, to say that this should not be done unless the editorial team stipulate otherwise. It just might be worth considering if it makes logical sense to present the hypotheses before the methods (i.e., indicative of a hypothesis-led study) or as they are (i.e., indicative of an exploratory study).
>>>Response: We appreciate the suggestion, but we prefer to keep the hypotheses as they are.

Lines 432-435: Could it not be said that these differences between the methods support the idea of using both in tandem? The manuscript is very much focused on the competition between the techniques, but some exploration, however brief, of their compatibility might be interesting.
>>>Response: That’s a good idea. Thank you. We added the idea of using both methods in tandem. We had already stated that the methods were complementary and used them together to construct the food web.

Line 457: redundant "are" before "may"
>>>Response: Modified accordingly.

Lines 472-473: Again, the mock community results seem somewhat problematic for this statement given the primer selection problem.
>>>Response: Answered in the first response to this reviewer, and also in the response regarding lines 19-20.

Line 498: Is this metagenomic work by Srivathsan et al. not methodologically different from Lazaro? My understanding is that Lazaro is a distinct method, hence the new name.
>>>Response: Reviewer is correct. Modified accordingly.

Close
