# Supplementary material for: Metabarcoding versus mapping unassembled shotgun reads for identification of prey consumed by arthropod epigeal predators
Source: Gigascience. 2022 Mar 24;11:giac020. doi: 10.1093/gigascience/giac020 (PMC8952265; doi:10.1093/gigascience/giac020)
Supplement: giac020_Reviewer_1_Report_Original_Submission — Tuomas KankaanpÃ¤Ã¤, Ph.D. -- 10/22/2021 Reviewed [file giac020_reviewer_1_report_original_submission.pdf]

Reviewer Report

Title: Metabarcoding versus mapping unassembled shotgun reads for identification of prey consumed by arthropod epigeal predators

Version: Original Submission **Date:** 10/22/2021

Reviewer name: Tuomas KankaanpÃ¤Ã¤, Ph.D.

Reviewer Comments to Author:

Dear Dr. Paula
The manuscript has been improved considerably from the initial version. The introduction in now an enjoyable read with enough background on competing methodologies. The rest of the manuscript now offers sufficient detail to objectively assess the study and its results. The responses to reviewer queries were throughout and satisfactory. The supplementary materials are informative as well.
I still feel the methods comparison is somewhat unfair to metabarcoding, performance of which can be improved in several ways: e.g. processing individuals separately, while still sequencing them in a multiplexed single library; improving or adding primers; using blocking primers or optimising PCR conditions (40 cycles will definitely mask rare prey species due to the exponential nature of amplification). Most of these aspects are of course discussed, and attention is drawn to them by the a priori hypotheses not being confirmed.
Nevertheless, the article is helpful to anyone aiming to either further develop methods for molecular analysis of trophic interactions or to choose an existing protocol for ecological studies. The melting curve analysis elevates the study to a level required by the journal.
Thus, I only have few very small comments (line numbers refer to the word document).
Line 84: I know this was suggested by reviewer 1. but a "unanimous consensus" is a tautology
Line 98: should be: one adult aptera
Line 338: should be: Neither metabarcoding or Lazaro produced any false positives
Line 478: I would say higher detection of false positives instead of lower in this sentence to be consistent with the hypothesis and the previous two sentences. "this did not convert into a higher detection of true positive or a lower detection of false positive prey detections compared to Lazaro."
The reference list needs some touches to match journal guidelines. E.g. doi format, italisation of journal names.
Yours, Dr. Tuomas KankaanpÃ¤Ã¤, Ecology and Genetics unit, University of Oulu

Methods

Are the methods appropriate to the aims of the study, are they well described, and are necessary controls included? Choose an item.

Conclusions

Are the conclusions adequately supported by the data shown? Choose an item.

Reporting Standards

Does the manuscript adhere to the journal's guidelines on [minimum standards of reporting?]() Choose an item.

Choose an item.

Statistics

Are you able to assess all statistics in the manuscript, including the appropriateness of statistical tests used? Choose an item.

Quality of Written English

Please indicate the quality of language in the manuscript: Choose an item.

Declaration of Competing Interests

Please complete a declaration of competing interests, considering the following questions:

- Have you in the past five years received reimbursements, fees, funding, or salary from an organisation that may in any way gain or lose financially from the publication of this manuscript, either now or in the future?
- Do you hold any stocks or shares in an organisation that may in any way gain or lose financially from the publication of this manuscript, either now or in the future?
- Do you hold or are you currently applying for any patents relating to the content of the manuscript?
- Have you received reimbursements, fees, funding, or salary from an organization that holds or has applied for patents relating to the content of the manuscript?
- Do you have any other financial competing interests?
- Do you have any non-financial competing interests in relation to this paper?

If you can answer no to all of the above, write 'I declare that I have no competing interests' below. If your reply is yes to any, please give details below.

I declare that I have no competing interests.

I agree to the open peer review policy of the journal. I understand that my name will be included on my report to the authors and, if the manuscript is accepted for publication, my named report including any attachments I upload will be posted on the website along with the authors' responses. I agree for my report to be made available under an Open Access Creative Commons CC-BY license (http://creativecommons.org/licenses/by/4.0/). I understand that any comments which I do not wish to be included in my named report can be included as confidential comments to the editors, which will not be published.

Choose an item.

To further support our reviewers, we have joined with Publons, where you can gain additional credit to further highlight your hard work (see: https://publons.com/journal/530/gigascience). On publication of this paper, your review will be automatically added to Publons, you can then choose whether or not to claim your Publons credit. I understand this statement.

Yes Choose an item.
