# Supplementary material for: Metabarcoding versus mapping unassembled shotgun reads for identification of prey consumed by arthropod epigeal predators
Source: Gigascience. 2022 Mar 24;11:giac020. doi: 10.1093/gigascience/giac020 (PMC8952265; doi:10.1093/gigascience/giac020)
Supplement: giac020_Reviewer_2_Report_Original_Submission — Jordan Patrick Cuff, BSc MRes PhD -- 10/25/2021 Reviewed [file giac020_reviewer_2_report_original_submission.pdf]

Reviewer Report

Title: Metabarcoding versus mapping unassembled shotgun reads for identification of prey consumed by arthropod epigeal predators

Version: Original Submission **Date:** 10/25/2021

Reviewer name: Jordan Patrick Cuff, BSc MRes PhD

Reviewer Comments to Author:

The effort taken by authors to address the comments from the previous round of review is significant and admirable. The authors have clearly taken great care and effort to strengthen the manuscript, which has evidently led to some excellent additions. The experimental controls and more detail around the overall QC are very welcome additions. The additional information regarding the number of individuals per sample and sequencing depth are very valuable too. The differentiation in nomenclature between "DDSS" and "Lazaro" is a good idea (and the new name is great)!
Regarding the response to the comment about data cleanup, appreciably "post-bioinformatic" is quite vague and disregards any processes incorporated into the bioinformatics. The additional information and controls presented in the revised manuscript are an excellent addition. Many metabarcoding studies apply quality thresholds (sometimes arbitrary, e.g., remove taxa with <100 reads, sometimes more uniform and robust, e.g., remove any reads that comprise less than 1 % of the sample read count) to remove false positives that arise through contamination/error. Whilst the scale of these errors might be reduced in Lazaro (i.e., no PCR amplification of false positives), there are still many opportunities through which they may arise (e.g., environmental/lab contaminants, sequencing errors) so it would be interesting to know how these might best be tackled going forward. Given the novelty of these techniques, it is appreciably difficult to determine best practice, so it is great to see that this is now more overtly incorporated.
In the response to reviewers, it is mentioned that controls will not identify contamination that occurs after DNA extraction, but that is not always the case. A distinction between negative controls (e.g., extractions without input tissue or PCRs without input DNA) and blanks (e.g., unused combinations of used sample tags). Equally, running samples in triplicate (as is done for some aspects of this manuscript) can facilitate identification of post-extraction contamination. Of course, these are just suggestions for further applications and would be an unfair expectation of every such study given the financial upscaling associated. Whilst the authors are correct in suggesting that this type of contamination may occur infrequently and thus contamination may not be detected by these controls, the statement that this means the controls give false confidence in the lack of contamination should be revisited. Surely not screening for this contamination at all is much worse in this regard than checking and not discovering any contamination.
Regarding cannibalism, is there no potential to identify instances of cannibalism in future studies by looking for intraspecific genetic variation? Obviously this may be beyond the scope of this study, but it would certainly be a great benefit over metabarcoding if it could be achieved. Even if only by incorporation of phylogenetic distances between individual predators in the analysis, there seems to be

great potential for lateral streams of data using this approach which appear to be otherwise untapped.
Some specific comments:

Lines 19-20: Here it is stated that metabarcoding did not detect any prey and that Lazaro detected 43-71 %. Without stating the possible reasons for the poor detection of prey by metabarcoding, this seems to be an unfair representation, since it seems to come down to primer choice. Positive results from ecoPCR for amplification of the target species is mentioned later as a justification for it not being a primer problem, but importantly ecoPCR is a very simple simulation of PCR, neglecting critical aspects of primer success such as 3' proximity and adjacency of mismatches. Ultimately, it seems that primer mismatch or preferential amplification of predator DNA were likely causes of this issue, which is of course an indictment of metabarcoding more generally anyway (i.e., primer mismatches are unpredictable and can hamper taxonomic coverage sporadically), but it could obviously have been circumvented by alternative primer choice, or by using multiple primer pairs. Ultimately though, to test whether this primer mismatch issue is the cause of this issue, one could simply diagnostically test tissue extracts from these taxa with the primers in question.

Line 27: It is stated that Lazaro and metabarcoding are "similar in these respects" and then two percentages are given, but what do these refer to? Detection of true positives, false positives and false negatives, and taxonomic resolution are all mentioned, so it is not clear which these percentages pertain to.

Lines 28: The statement that metabarcoding detected 350X more true prey reads is surely uninformative because those reads are amplified, so what does this tell us? It is then stated that this doesn't convert into a higher number of true positive prey detections, which is to be expected. It is probably a valuable statement for the manuscript, but it doesn't feel important enough for the abstract when there are so many great findings already there.

Line 34: I feel that the "may" and "can" should be flipped. Whilst there is some debate around quantification of metabarcoding reads, there seems to be a general consensus that even the best attempts require additional experimental data (i.e., it cannot be quantitative in isolation). Lazaro, however, shows promise for quantification, but without more studies and data from alternative applications, this seems speculative still (thus "may" could be better).

Line 57: "do not assemble"

Line 57: "prior" may be more appropriate than "previously"

Line 80: This is the first main text mention of Lazaro; it would be valuable to introduce it. It is introduced on line 88; perhaps these could be switched. It might be nice to include briefly the justification for the name too

Lines 96-103: Was it visually confirmed that all seven prey were consumed by the predators?

Lines 286-307: It's unconventional to present hypotheses at the end of the methods. It would seem more logical to include them at the end of the introduction. This is not, however, to say that this should not be done unless the editorial team stipulate otherwise. It just might be worth considering if it makes logical sense to present the hypotheses before the methods (i.e., indicative of a hypothesis-led study) or as they are (i.e., indicative of an exploratory study).

Lines 432-435: Could it not be said that these differences between the methods support the idea of using both in tandem? The manuscript is very much focused on the competition between the techniques, but some exploration, however brief, of their compatibility might be interesting.

Line 457: redundant "are" before "may"
Lines 472-473: Again, the mock community results seem somewhat problematic for this statement given the primer selection problem.
Line 498: Is this metagenomic work by Srivathsan et al. not methodologically different from Lazaro? My understanding is that Lazaro is a distinct method, hence the new name.

Methods

Are the methods appropriate to the aims of the study, are they well described, and are necessary controls included? Choose an item.

Conclusions

Are the conclusions adequately supported by the data shown? Choose an item.

Reporting Standards

Does the manuscript adhere to the journal's guidelines on minimum standards of reporting? Choose an item.

Choose an item.

Statistics

Are you able to assess all statistics in the manuscript, including the appropriateness of statistical tests used? Choose an item.

Quality of Written English

Please indicate the quality of language in the manuscript: Choose an item.

Declaration of Competing Interests

Please complete a declaration of competing interests, considering the following questions:

- Have you in the past five years received reimbursements, fees, funding, or salary from an organisation that may in any way gain or lose financially from the publication of this manuscript, either now or in the future?
- Do you hold any stocks or shares in an organisation that may in any way gain or lose financially from the publication of this manuscript, either now or in the future?
- Do you hold or are you currently applying for any patents relating to the content of the manuscript?
- Have you received reimbursements, fees, funding, or salary from an organization that holds or has applied for patents relating to the content of the manuscript?

- Do you have any other financial competing interests?
- Do you have any non-financial competing interests in relation to this paper?

If you can answer no to all of the above, write 'I declare that I have no competing interests' below. If your reply is yes to any, please give details below.

I declare that I have no competing interests.

I agree to the open peer review policy of the journal. I understand that my name will be included on my report to the authors and, if the manuscript is accepted for publication, my named report including any attachments I upload will be posted on the website along with the authors' responses. I agree for my report to be made available under an Open Access Creative Commons CC-BY license (http://creativecommons.org/licenses/by/4.0/). I understand that any comments which I do not wish to be included in my named report can be included as confidential comments to the editors, which will not be published.

Choose an item.

To further support our reviewers, we have joined with Publons, where you can gain additional credit to further highlight your hard work (see: https://publons.com/journal/530/gigascience). On publication of this paper, your review will be automatically added to Publons, you can then choose whether or not to claim your Publons credit. I understand this statement.

Yes Choose an item.
